# Immunoproteomic Analysis of *Trichinella britovi* Proteins Recognized by IgG Antibodies from Meat Juice of Carnivores Naturally Infected with *T. britovi*

**DOI:** 10.3390/pathogens11101155

**Published:** 2022-10-06

**Authors:** Aleksandra Cybulska

**Affiliations:** Witold Stefański Institute of Parasitology, Polish Academy of Sciences, Twarda 51/55, 00818 Warsaw, Poland; cybulska.aleksandra@twarda.pan.pl; Tel.: +48-22-697-8974

**Keywords:** *Trichinella britovi*, wildlife, antibodies, meat juice immunology

## Abstract

Infection with *Trichinella* nematodes elicits non-specific and specific immune responses; these depend on the dose of infection, the nematode, and the host species. Few studies have examined the presence of specific antibodies against *Trichinella* spp. in the meat juice of wild animals. The aims of the study were to determine the prevalence of antibodies against *Trichinella* spp. in meat juice and to identify the specific proteins reacting with the meat juice from free-living carnivores naturally infected with the parasite. Meat juice samples were taken from foxes, badgers, raccoon dogs, and martens and tested with indirect ELISA. Antibodies against *Trichinella* spp. were detected in 10% of foxes and 46% of raccoon dogs. The ELISA results were confirmed by immunoblot, which revealed different protein patterns in meat juice from red foxes, raccoon dogs, and badgers. The most frequently observed bands were sent for further analysis by liquid chromatography coupled with tandem mass spectrometry (LC-MS/MS) for the detection of *Trichinella britovi* immunogenic proteins. The results confirm the presence of proteins such as serine protease and heat shock proteins associated with *Trichinella* infection. These findings provide that meat juice is a useful matrix for proteomic analysis.

## 1. Introduction

The parasitic nematodes of the genus *Trichinella* have a global reach, where they circulate in both domestic and sylvatic cycles [1]. In Poland, and Europe in general, the most common *Trichinella* species observed in the sylvatic cycle is *Trichinella britovi*, which is found in animals such as red foxes, wolves, lynxes, martens, badgers, golden jackals, raccoons, and raccoon dogs [2,3,4,5,6,7,8,9,10,11,12,13]. Their life cycle comprises three major stages, viz. muscle larvae, adult worms, and new-born larvae, which are observed in a single host. 

All developmental stages of *Trichinella* elicit an immune response, including IgG antibodies, which can be used for the serological detection of *Trichinella* spp. infection [14]. Infection with nematodes of the genus *Trichinella* causes both non-specific and specific immune responses, which can vary depending on the dose of infection, and the species of *Trichinella* and host. The presence of anti-*Trichinella* antibodies and specific proteins or antigens may be observed in serum or plasma and meat juice. Both serum and plasma are easy to collect and are widely used as sources of proteins in a highly soluble form [15]; indeed, there are thousands of proteins that are released by cells and tissues [16]. Serum is a complex biological matrix, and the general health status of the specimen and the presence of parasitic, bacterial, or viral infections are reflected in changes in its proteomic pattern [16]. However, in many cases, it is not possible to collect serum samples from wild animals. In such cases, meat juice, consisting of a mixture of intra- and extracellular fluid, blood, and lymph, might provide an alternative matrix that can be easily obtained for post-mortem analysis from carcasses of free-living animals [17]. It has been demonstrated that meat juice samples may be successfully used as a matrix in immunological surveillance of parasitic (e.g., *Toxoplasma gondii*, *Neospora caninum*) [18,19,20], bacterial (e.g., *Salmonella*, *Brucella*) [21,22], and viral (e.g., Japanese encephalitis virus, hepatitis E virus) [23] infections in animals. Moreover, meat juice samples have been also used as a matrix for immunological monitoring of the occurrence of anti-*Trichinella* IgG antibodies in domestic and wild animals [24,25,26,27,28,29]. When performing ELISA, the dilution factor has to be 10 times higher for the serum than for the meat juice sample; however, both sample types can use the same cut-off value to categorize immunopositive from immunonegative animals [21].

Although *T. spiralis* has typically been regarded as the most significant pathogen related to public health, there is a need for further studies on the antigenic profile associated with *T. britovi* infection [30], which occurs more frequently among wild carnivores [1,12,31,32]. In natural environments, carnivorous animals play an important role in the transmission of *Trichinella* nematodes, including *T. britovi*. Carcasses of infected animals may become a source of invasion for other animals, especially for wild boars, for which unexamined meat is known to be a source of *Trichinella* infection in humans [33,34,35].

Although in the domestic cycle the immune response during *Trichinella* infection has been widely studied in pigs, under controlled conditions with a known infection dose and species of *Trichinella* [14,30,36,37,38,39], the immunoprevalence of anti-*Trichinella* antibodies has been also examined in many animals species connected to the sylvatic cycle [13,24,26,40,41,42,43,44,45,46] as the main reservoir of *Trichinella* nematodes in the environment. Infections in wildlife occur in a natural way—the dose of infection, the timing, and whether they occur once or repeatedly remain unknown. Therefore, it is important to gain a deeper understanding of immune response during *Trichinella* infection in free-living animals. 

As the present literature contains little or no data concerning the presence of specific proteins/antigens related to *Trichinella* infection in the meat juice of infected free-living animals, the aims of the present study were twofold: (1) to determine the prevalence of antibodies against *Trichinella* spp. in meat juice; (2) to examine the protein profile using meat juice collected from free-living animals.

## 2. Results

### 2.1. ELISA and Immunoblot Results

**Figure 1:**
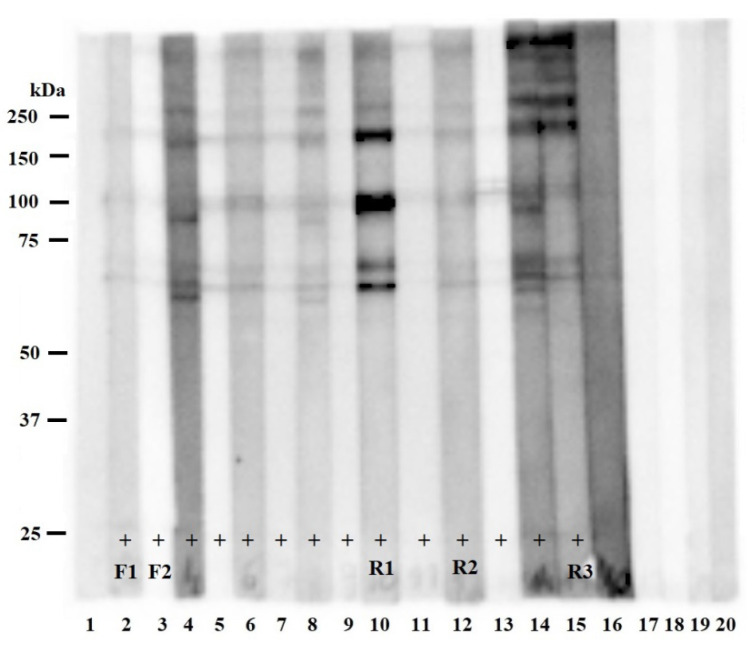
The immunoblot analysis of *T. britovi* ML C antigens incubated with meat juice samples taken from different animals. Lanes 1–3—meat juice samples taken from foxes; lanes 4–15—meat juice samples taken from raccoon dogs; lane 16—meat juice sample taken from a badger; lanes 17–20—meat juice samples taken from badger, marten, fox, and raccoon dog (respectively), which were used as a negative control for immunoblot. Lines marked with + were recognized as positive in ELISA and immunoblot.

The ELISA testing found 14 of the 88 examined meat juice samples to be positive for the presence of *Trichinella* antibodies. Antibodies against *Trichinella* spp. were detected in two meat juice samples from foxes (10%) and 12 meat juice samples from raccoon dogs (46%). Additionally, one fox sample had a borderline result in ELISA. The positive and borderline results from ELISA were confirmed by immunoblot, which revealed a *Trichinella*-specific protein pattern recognized by meat juice obtained from red foxes (*n* = 2), raccoon dogs (*n* = 12), and a badger (*n* = 1). The characteristic bands typically migrated with a molecular weight (MW) between 50 and 250 kDa (Figure 1). 

The presence of *T. britovi* larvae was confirmed in all animals recognized as immunopositive by both methods: ELISA and immunoblot (Figure 1, lanes 2–15), with different intensity of infection: animal 2: 1.61 larvae per gram (LPG); animal 3: 0.29 LPG, animal 4: 37.05 LPG, animal 5: 165.67 LPG, animal 6: 17.93 LPG, animal 7: 31.13 LPG, animal 8: 2.27 LPG, animal 9: 7.03 LPG, animal 10: 14.21 LPG, animal 11: 331.26 LPG, animal 12: 19.04 LPG, animal 13: 180,39 LPG, animal 14: 3.08 LPG, and animal 15: 4.85 LPG (the number of animals corresponds with the number of lanes in Figure 1). The results concerning the intensity of infection in raccoon dogs were partially published by Cybulska et al. (2019) [12]. Moreover, the result was borderline by ELISA, but negative by immunoblot in the case of one fox (Figure 1, lane 1), which was also *T. britovi*-positive in digestion. The intensity of infection was around one LPG. Immunoblot confirmed also the presence of anti-*Trichinella* antibodies in a meat juice sample from one badger that was not positive in ELISA (Figure 1, lane 16). Interestingly, this badger was infected with *T. britovi*, with a low intensity of infection (0.13 LPG).

Lanes 17–20 (Figure 1) represent meat juice samples used as a negative control for immunoblot. These samples were taken from badger, marten, fox, and raccoon dog (respectively), which were negative in digestion and in ELISA.

### 2.2. Immunoblot Analysis

**Figure 2:**
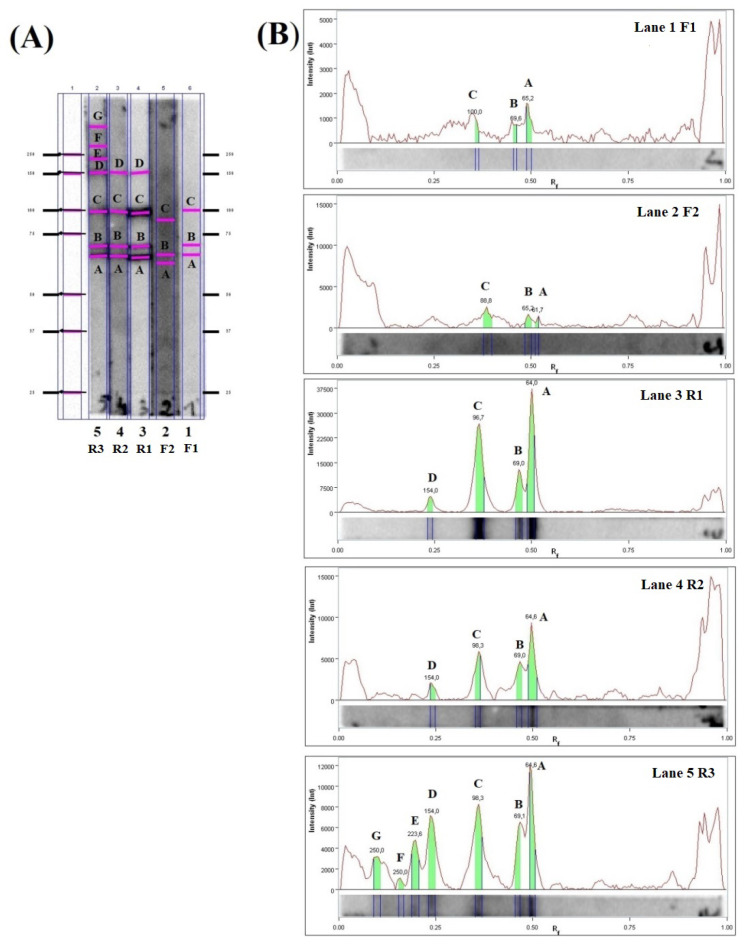
(**A**) The immunoblot analysis of *T. britovi* ML C antigens incubated with selected meat juice samples. (**B**) Signal intensity and relative migration values for *T. britovi* ML C antigens incubated with examined meat juice samples. Lanes 1–2—meat juice samples from foxes (F1, F2, respectively); Lanes 3–5—meat juice samples from raccoon dogs (R1, R2, R3, respectively). No. of bands—A, B, C, D, E, F, G.

Two meat juice samples from red foxes (F1, F2), and three from raccoon dogs (R1, R2, R3) were classified for further analysis to detect the specific proteins in the characteristic bands (Figure 2). The immunoreactive bands matched the corresponding protein bands on silver-stained gels and were selected for further LC-MS/MS analysis.

### 2.3. LC-MS/MS Analysis

Three gel pieces were obtained for both examined meat juice samples from red foxes (bands: A, B, C), four gel pieces for the two meat juice samples from raccoon dogs (bands: A, B, C, D), and seven gel pieces for the single meat juice sample from another raccoon dog (bands: A, B, C, D, E, F, G) (Figure 2). LC-MS/MS analysis revealed the presence of 279 proteins in the bands recognized by meat juice from fox F1; 209 proteins in the bands recognized by meat juice from fox F2; 357 proteins in the bands recognized by meat juice from raccoon dog R1; 339 proteins in the bands recognized by meat juice from raccoon dog R2; and 393 proteins in the bands recognized by meat juice from raccoon dog R3. More detailed information is presented in Supplementary Material Table S1 (foxes) and Table S2 (raccoon dogs). A comparative analysis of the proteins identified from the five examined meat juice samples indicates that all samples share 117 proteins, including heat shock proteins or paramyosin, which are known immunomodulators (Table 1). In addition, samples from foxes share 136 proteins, and those from raccoon dogs share 238 proteins. Several proteins were identified from multiple bands; these may correspond to protein isoforms or post-translational modifications (Tables S1 and S2). 

### 2.4. Gene Ontology (GO) Analysis

**Figure 3:**
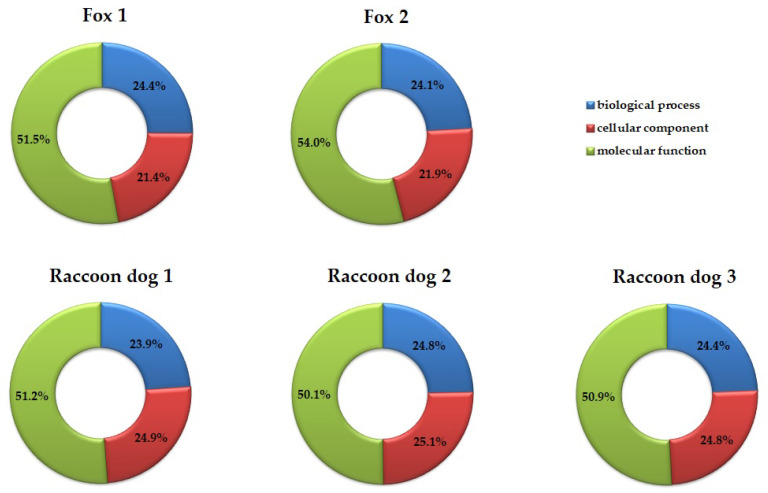
The percentage share of the number of proteins derived from three categories: biological process, cellular component, and molecular function.

Gene Ontology (GO) analysis was used to identify proteins reacting with antibodies present in examined meat juice samples, and these proteins were categorized according to their molecular function, cellular component, and biological process. The percentage share of the number of identified proteins by categories is shown in Figure 3. The data indicates that 222 proteins are associated with biological processes for F1, 159 for F2, 284 for R1, 282 for R2, and 312 for R3. Regarding biological processes, the highest number of proteins are involved in translation and protein folding processes. Briefly, 220 proteins were associated with cellular components for F1, 145 for F2, 295 for R1, 186 for R2, and 317 for R3. The identified proteins are mainly associated with organelles (nucleus, ribosome) and cell parts (cytoplasm, components of membrane). Molecular functions were also found: 469 proteins for F1, 357 for F2, 608 for R1, 571 for R2, and 651 for R3. The predominant types are related to binding (e.g., ion binding, ATP binding, RNA binding) and catalytic activity (e.g., ATP hydrolysis activity, GTPase activity). 

## 3. Discussion

Trichinellosis is a disease with a global range. Unsurprisingly, there is a great interest in identifying peptide or protein biomarkers that could be used as markers to identify early *Trichinella* infection in humans and domestic animals (diagnostic properties) or as vaccines against *Trichinella* infection, e.g., in pigs (immunoprotective properties). Although recent studies have focused on the immunoreactive proteins in host serum and plasma during *Trichinella* infection, they have mainly been based on 2DE-electrophoresis examination coupled with LC-MS/MS, or 2DE electrophoresis followed by MALDI-TOF mass spectrometry of human, mouse, or pig samples [30,36,37,38,39,47,48,49,50,51,52,53]. In contrast, the present paper employs standard 1DE electrophoresis coupled with LC-MS/MS analysis to obtain preliminary findings; it is the first proteomic analysis of proteins recognized by meat juice samples collected from wildlife. These findings provide new insight into the immune response to *Trichinella* infection in wild animals. Free-living animals constitute a natural reservoir for various parasites, including *Trichinella* nematodes, and unlike laboratory-based studies, the infection dose and the number of potential re-infections are unknown. Therefore, a thorough understanding of the protein profiles present in free-living animals is needed to provide an accurate picture of the course of a parasite infection.

The entire life cycle of the *Trichinella* nematodes takes place in a single host, following the ingestion of meat containing infectious *Trichinella* larvae. *Trichinella* displays three main antigenic stages: muscle larvae (ML), adult worms (Ad), and newborn larvae (NBL) [14]. The muscle larvae are released in the stomach, from where they migrate to the epithelial cells of the small intestine, where they molt and transform into adult worms. After coupling, the females start to deliver NBLs, which move mainly through the blood circulation to reach the striated muscle, where they finally develop into MLs [1]. All developmental stages of *Trichinella* stimulate an immune response and produce antigens which can be used for the serological detection of *Trichinella* spp. infection. Additionally, some research suggests that the *Trichinella* antigens produced by the three developmental stages are stage-specific [14] and they stimulate or suppress the host immune response to varying degrees. 

Most studies have used serum/plasma samples to determine the protein profile characteristic of *Trichinella* infection [14,30,36,37,38,39,47,48,49,51,52,53]. However, while serum or plasma are not always available for analysis, meat juice samples provide an adequate, alternative matrix that can be easily obtained for post-mortem analysis, especially from free-living animals. The use of meat juice also offers the added benefit of studying protein profiles at the exact location where *Trichinella* larvae localize, thus providing potential new insight into parasite infection, especially when investigating stage-specific antigens for muscle larvae. 

In the present studies, different proteins recognized by meat juice samples were observed not only between two of the examined animal species but also between those of the same species (Figure 2A, Table 1). The obtained results may suggest that the examined animals were infected with different doses of the parasite with a different duration of infection. However, in the present paper, examined meat juice samples were taken from animals infected with *T. britovi* in the late stage of infection, with larvae present in the muscle. It needs to be underlined that the intensity of infection in examined animals differs between specimens. Additionally, in immunoblot, the characteristic bands typically migrated between 50 and 250 kDa, with some differences between animals (Figure 1). In another study, performed with sera of raccoon dogs infected with *T. spiralis* or *T. nativa* the bands were observed at around 100 kDa, with a group of bands between 76 and 40 kDa [40]. Contrary to this, research conducted with the use of the serum of foxes infected with *Trichinella nativa*, revealed that most bands migrate between 40 and 60 kDa [41].

Although most trichinellosis in humans is caused by *T. spiralis*, some cases of *T. britovi* infection have recently been observed [54,55,56,57]. Furthermore, several papers have described the analysis of crude and excretory-secretory proteins characteristic of *T. britovi* [14,30,36,38,39,48]. These studies, conducted using human or *T. britovi*-infected pig serum, identified some proteins characteristic of *T. britovi* infection, including those that play a role in the migration of NBL in host tissue or those with immunomodulatory potential. 

The aim of the present study was to determine the pattern of proteins reacting with IgG antibodies in meat juice samples collected from free-living animals infected with *T. britovi*. It was found that antibodies present in meat juice samples react with proteins that are associated with *T. britovi* infection; in addition, the majority of them appear to be involved in metabolic processes and host–parasite interaction (e.g., the mechanisms of the parasite invasion of host tissue, larval migration, larval molting, immunomodulation), such as heat shock proteins, a serine protease, paramyosin, a poly-cysteine tailed protein, and deoxyribonuclease-2-alpha [14,30,36,38,39]. The identified proteins are compared with the literature data on proteins specific to *T. britovi* infection in Table 2. 

LC-MS/MS analysis allowed to reveal the presence of a wide range of proteins known to take part in tissue invasion, larval migration, larval molting, immune modulation, and metabolic processes. For example, the subunits of 26S protease have been found to be involved in cell cycle and transcription regulation, apoptosis, and the oxidative stress response [38]. Deoxyribonuclease-2-alpha plays a role in the invasion of host tissue and in evading host defense [58,59]. In addition, intermediate filament proteins were observed in all examined samples. These proteins are known as structural elements of the cellular cytoskeleton and are responsible for worm growth [60]. As well as the filament proteins, myosin-4 and paramyosin also play a role in parasite growth. The first is responsible for cellular component organization and actin filament depolymerization, thus facilitating parasite growth and development [14], while the second influences muscle length and stability. It has been observed that paramyosin from helminths serves not only as a structural protein but also as an immunomodulatory agent [61]. 

In addition, peroxiredoxin-2 protects nematodes from hazardous reactive oxygen species (ROS) during invasion [62], and serine protease 30 plays a crucial role in the host tissues during cell invasions and larval molting [14]. Interestingly, all examined samples were found to contain hypothetical protein T03_17187; this protein has been found to share more than 90% identity with the multi-cystatin-like domain protein (CLP), which is a promising immunoreactive protein [48,63,64].

These proteins are known to be involved in the biological processes of the parasite, such as intracellular protein transport, stress response, glycolytic processes, tricarboxylic acid cycle, protein folding, and translation; they also form parts of important cellular components, such as the cytosol, mitochondrion, proteasome complex, small ribosomal subunit, ribosome, nucleus, cytoplasm, and integral membrane component. They are also involved in significant molecular functions, mainly as catalytic activity (ATP hydrolysis activity, GTPase activity, transferase activity, hydrolase activity, kinase activity) and binding activity to ions, ATP, and RNA. However, it has to be emphasized that differences in the protein profile were observed not only between two examined animal species but also between specimens of the same species (Table S3). This is not surprising, as it has been proposed that the host immune response may vary depending on the dose and phase of infection, parasite, and host species [65,66,67]. Interestingly, the percentage share of the number of proteins derived by three categories, viz. biological process, cellular component, and molecular function, remained comparable between the five examined meat juice samples (Figure 3).

The present study addresses the previous lack of data regarding the presence of *T. britovi* antibodies in meat juice from free-living animals. *T. britovi* is known to be widespread among wild animal species and can be transmitted to humans. Hence, there is a pressing need to identify *T. britovi*-specific antigens which may be used to diagnose infection in humans or support vaccine development [64]. Indeed, searching for markers or proteins with vaccine potential among free-living animals infected with the parasite is in line with the One Health Concept. Nevertheless, there is a need to extend the study using more advanced proteomics techniques such as 2DE electrophoresis coupled with LC-MS/MS analysis.

## 4. Materials and Methods

### 4.1. Collection of Material and Research Scheme

The study was conducted on 20 red foxes (*Vulpes vulpes*), 26 raccoon dogs (*Nyctereutes procyonoides*), 24 martens (*Martes* spp.), and 18 badgers (*Meles meles*). The animals were acquired between 2013 and 2016 through hunting activities within Project Life+ (NAT/PL/428). Meat juice samples were collected on the occasion of the digestion method for the presence of *Trichinella* spp. larvae according to EC Regulation No. 2075/2005 [68], and the *Trichinella* species was determined using multiplex PCR described by Zarlenga et al. 1999 [69], with some modification by Cybulska et al. 2019 [12]. Carcasses were transported to the Witold Stefański Institute of Parasitology, Polish Academy of Sciences (Warsaw, Poland). Muscle samples were taken during dissection and then stored at −20 °C for further analysis. The day before digestion, muscle samples (diaphragms and limb muscles) were thawed at room temperature (RT) and meat juice samples were collected individually into sterile tubes. Next, the obtained samples were stored at −20 °C for further tests: ELISA and immunoblotting.

### 4.2. ELISA

The presence of antibodies against *Trichinella* from the meat juice samples was detected using a commercial ELISA kit (ID Screen *Trichinella* Indirect Multi-species, IDvet, Grabels, France), according to the manufacturer’s instructions. The meat juice sample dilution was 1:2, according to the protocol. The optical density (O.D.) was measured at a wavelength of 450 nm using an EL*800 ELISA automated plate reader (BioTek, Winooski, VT, USA). The cutoff was calculated based on the sample-to-positive (S/P) percentage according to the formula S/P = [optical density (OD) sample − OD negative control (NC)/OD positive control (PC) − OD NC] × 100. Samples with S/P ≥ 30% were considered as positive; with 25% ≤ S/P% ≤ 30% were considered as borderline; and with S/P% ≤ 25% were considered as negative. 

### 4.3. Preparation of Crude Antigens from Muscle Larvae of T. britovi 

The Balb/C mice were orally infected with 500 *T. britovi* (ISS002) muscle larvae (ML), then maintained for at least three months, and then euthanized. Next, to prepare muscle larvae crude antigens (ML C antigens), the MLs were isolated using digestion. The recovered MLs were subsequently purified several times with water through succeeding steps of sedimentation in cylinders. After the final sedimentation, the MLs were collected into 1.5 mL tubes. The larval pellet was extensively washed three times in phosphate-buffered saline (PBS). After that, the larval pellet was washed three times and mixed with lysis buffer as described previously by Bień et al. 2013 [50]. The mixture was homogenized in a Potter-Elvehjem Tissue Homogenizer and disintegrated by sonication (OMNI International Tissue Homogenizer). Next, the solution was centrifuged at 14,000 RPM at 4 °C for 10 min.

### 4.4. SDS–Polyacrylamide Gel Electrophoresis (SDS–PAGE) and Immunoblot

The ML C antigens (approximately 10 μg per well) were run on a 12% SDS-PAGE gel with 4% stacking gel at 180 V constant voltage for 50 min. After electrophoresis, the proteins were transferred from the gel to a nitrocellulose sheet (Bio-Rad, Hercules, CA, USA) at 95V for 55 min using Mini-Protean Tetra Cell (Bio-Rad, Hercules, CA, USA). Nitrocellulose strips (NCS) were blocked with Pierce Protein-Free T20 Blocking Buffer (Thermo Fisher Scientific, Waltham, MA, USA) for one hour at RT, and then washed three times in PBS–Tween buffer (pH = 7.2). Following this, the NCSs were incubated with shaking for one hour at RT with meat juice samples diluted 1:20 in Pierce Protein-Free T20 Blocking Buffer (Thermo Fisher Scientific, Waltham, MA, USA). Afterward, the NCSs were washed and then incubated with shaking for one hour at RT with secondary antibody, i.e., horse radish-peroxidase-conjugated Anti-dog IgG (Sigma-Aldrich, Saint Louis, MO, USA), diluted by 1:25,000. Finally, after washing, the NCSs were developed using SIGMAFAST™ 3,3′-diaminobenzidine (Sigma-Aldrich, Saint Louis, MO, USA) and visualized with the Chemi Doc MP Imaging System (Bio-Rad, Hercules, CA, USA).

### 4.5. Proteomic Analyses—Mass Spectrometry (MS) and Protein Identification

Bands of interest were manually excised from the gel and analyzed by liquid chromatography coupled to a mass spectrometer (LC-MS/MS) in the Laboratory of Mass Spectrometry, Institute of Biochemistry and Biophysics, Polish Academy of Sciences (Warsaw, Poland). The obtained peptide masses and fragmentation spectra were matched to the NCBIProt database, with a Nematoda filter using the Mascot search engine. All proteins identified in the Mascot search were compared withthe UniProtKB database (https://www.uniprot.org/; accessed on 29 August 2022) and QuickGO (http://www.ebi.ac.uk/QuickGO/; accessed on 29 August 2022) and classified in gene ontology (GO) in accordance with their molecular functions, biological processes, and cellular component information.

## 5. Conclusions

The findings show that meat juice is a useful material for studying antibodies from animals naturally infected with *T. britovi*. The use of proteomic analysis reveals the presence of proteins known to take part in tissue invasion, larval migration, larval molting, immune modulation, and metabolic processes. Additionally, the mentioned proteins are immunogenic, and they are recognized by host antibodies. The results indicate also that the IgG-response may differ between host species in wildlife. A comprehensive understanding of immune response in free-living animals may provide an accurate picture of parasite infection.

## Figures and Tables

**Table 1:** Alphabetical list of 117 proteins common for five examined meat juice samples.

Protein	Access to Genbank	Protein	Access to Genbank
1,5-anhydro-D-fructose reductase	KRY43899.1	Lysosomal aspartic protease, partial	KRY49432.1
2,3-bisphosphoglycerate-independent phosphoglycerate mutase	KRY48325.1	Malate dehydrogenase, cytoplasmic	KRY57329.1
26S protease regulatory subunit 4, partial	KRY52921.1	Mannose-6-phosphate isomerase, partial	KRY55073.1
26S proteasome non-ATPase regulatory subunit 6, partial	KRY50659.1	Mediator of RNA polymerase II transcription subunit 23	KRY54660.1
32 kDa beta-galactoside-binding lectin	KRY59236.1	Medium-chain specific acyl-CoA dehydrogenase, mitochondrial, partial	KRY52707.1
3-ketoacyl-CoA thiolase, mitochondrial	KRY51064.1	Mitochondrial succinyl-CoA ligase subunit beta-like protein	KRY60682.1
4-hydroxybutyrate coenzyme A transferase, partial	KRY56869.1	Mitochondrial-processing peptidase subunit alpha	KRY50357.1
60 kDa heat shock protein, mitochondrial	KRY51676.1	Mitochondrial-processing peptidase subunit beta	KRY53080.1
60S acidic ribosomal protein P0	KRY54528.1	Mitochondrial isocitrate dehydrogenase, partial	KRY60163.1
60S ribosomal protein L4	KRY61057.1	Myophilin	KRY53866.1
Actin-related protein 2/3 complex subunit 2	KRY56438.1	NADP-dependent malic enzyme, mitochondrial, partial	KRY59664.1
Adducin-related protein 1	KRY47021.1	Obg-like ATPase 1	KRY54762.1
Adenylosuccinate lyase	KRY47589.1	Paramyosin, partial	KRY49321.1
Adenylosuccinate synthetase, partial	KRY50054.1	Peroxiredoxin-2, partial	KRY49990.1
ADP-ribose pyrophosphatase, mitochondrial	KRY48334.1	Phosphate carrier protein, mitochondrial, partial	KRY57035.1
Alpha-L-fucosidase	KRY54685.1	Phosphoglucomutase-1	KRY58984.1
Aspartate aminotransferase, mitochondrial, partial	KRY49869.1	Phosphoinositide 3-kinase regulatory subunit 4, partial	KRY53638.1
Aspartate--tRNA ligase, cytoplasmic	KRY49144.1	Polyubiquitin	KRY46475.1
ATP synthase subunit alpha, mitochondrial, partial	KRY49394.1	Polyubiquitin-like protein, partial	KRY24237.1
Calpain clp-1	KRY60775.1	Polyubiquitin-B, partial	KRY44570.1
Calreticulin, partial	KRY53845.1	Polyubiquitin-C	KRY55776.1
cAMP-dependent protein kinase regulatory subunit	KRY58719.1	Proliferation-associated protein 2G4, partial	KRY53054.1
Chymotrypsin-like elastase family member 2A, partial	KRY59723.1	Propionyl-CoA carboxylase alpha chain, mitochondrial	KRY51819.1
Cleavage and polyadenylation specificity factor subunit 2	KRY53654.1	Propionyl-CoA carboxylase beta chain, mitochondrial, partial	KRY52668.1
Cytochrome b-c1 complex subunit 2, mitochondrial, partial	KRY49815.1	Protein arginine N-methyltransferase 1, partial	KRY51927.1
Cytosol aminopeptidase	KRY54953.1	Protein ERGIC-53, partial	KRY52865.1
Deoxyribonuclease-2-alpha	KRY53116.1	putative [pyruvate dehydrogenase (acetyl-transferring)] kinase, mitochondrial, partial	KRY57184.1
Dihydrolipoyllysine-residue acetyltransferase component of pyruvate dehydrogenase complex, mitochondrial	KRY46167.1	putative aconitate hydratase, mitochondrial	KRY56796.1
Dihydropyrimidinase-related protein 3	KRY48683.1	putative aminopeptidase W07G4.4	KRY54333.1
Diphthine--ammonia ligase, partial	KRY47980.1	putative arginine kinase F46H5.3	KRY58392.1
DnaJ-like protein dnj-20	KRY45252.1	putative oxidoreductase-like protein	KRY46863.1
Dynein heavy chain, cytoplasmic	KRY59544.1	putative phosphoglycerate kinase	KRY46311.1
Elongation factor G, mitochondrial	KRY55228.1	putative pyruvate dehydrogenase E1 component subunit alpha, mitochondrial	KRY58181.1
Endoplasmic reticulum resident protein 44	KRY50742.1	Rab GDP dissociation inhibitor alpha	KRY54650.1
Endoplasmin, partial	KRY54651.1	secretion antigen precursor	CAD86782.1
Eukaryotic initiation factor 4A, partial	KRY53637.1	Serine hydroxymethyltransferase	KRY56265.1
Eukaryotic initiation factor 4A-III	KRY49326.1	Serine protease 30	KRY58838.1
Far upstream element-binding protein 2	KRY57083.1	Snake venom 5’-nucleotidase	KRY49427.1
Fructose-bisphosphate aldolase 1, partial	KRY52953.1	Solute carrier family 2, facilitated glucose transporter member 3	KRY47169.1
Glucose-6-phosphate isomerase	KRY50483.1	Splicing factor 3A subunit 3	KRY56136.1
Glyceraldehyde-3-phosphate dehydrogenase 1	KRY59635.1	Stress-induced-phosphoprotein 1	KRY50814.1
Glycogen phosphorylase, partial	KRY47089.1	Succinate dehydrogenase [ubiquinone] flavoprotein subunit, mitochondrial	KRY54543.1
Glycogenin-1	KRY61196.1	Succinate-semialdehyde dehydrogenase, mitochondrial	KRY54971.1
Heat shock cognate 71 kDa protein, partial	KRY58599.1	Sulfide:quinone oxidoreductase, mitochondrial	KRY46292.1
Heat shock protein 83, partial	KRY48983.1	T-complex protein 1 subunit alpha	KRY49588.1
Histone-lysine N-methyltransferase EHMT2, partial	KRY52481.1	T-complex protein 1 subunit beta	KRY52365.1
hypothetical protein T03_13827	KRY56487.1	T-complex protein 1 subunit epsilon	KRY50385.1
hypothetical protein T03_17187	KRY50177.1	T-complex protein 1 subunit eta	KRY48081.1
hypothetical protein T03_539	KRY55455.1	T-complex protein 1 subunit gamma	KRY47292.1
hypothetical protein T03_7459	KRY60639.1	T-complex protein 1 subunit theta	KRY50910.1
hypothetical protein T03_8666, partial	KRY58917.1	Transketolase	KRY48205.1
hypothetical protein T03_8694	KRY46541.1	Transmembrane protease serine 5	KRY50806.1
hypothetical protein T03_9489	KRY46491.1	Transmembrane protease serine 9	KRY58843.1
Ig-like and fibronectin type-III domain-containing protein C25G4.10	KRY58616.1	Trifunctional enzyme subunit beta, mitochondrial	KRY48050.1
Intermediate filament protein B, partial	KRY59373.1	Tubulin alpha-3 chain	KRY50534.1
Intermediate filament protein ifa-1	KRY45949.1	Type I inositol 1,4,5-trisphosphate 5-phosphatase, partial	KRY50866.1
Kynurenine--oxoglutarate transaminase 3	KRY54139.1	Ubiquitin carboxyl-terminal hydrolase 14, partial	KRY58645.1
L-2-hydroxyglutarate dehydrogenase, mitochondrial	KRY56323.1	Uncharacterized protein T03_9851	KRY58607.1
Leukocyte elastase inhibitor C, partial	KRY55578.1		

**Table 2:** A comparison of the proteins obtained in the present study with the literature data on proteins specific to *T. britovi* infection. The literature data given in the table were obtained from studies relating to antigens extracted from the ML stage.

Protein	Animal	GenBank Accession Number	Reference
Hypothetical protein T03_17187	fox	KRY50178.1	this study
	raccoon dog		this study
	human		[48]
	pig		[14]
Deoxyribonuclease-2-alpha	fox	KRY53318.1	this study
	raccoon dog		this study
	human	KRX47308.1	[48]
	pig		[14]
Transmembrane protease serine 9	fox	KRY59262.1	this study
	raccoon dog		this study
	human	KRY20911.1	[48]
	human	KRY21930.1	[48]
	pig	KRY58843.1	[38]
Intermediate filament protein B, partial	fox	KRY59373.1	this study
	raccoon dog		this study
	pig	KRY11608.1	[38]
	pig		[14]
Intermediate filament protein ifa-1	fox	KRY45949.1	this study
	raccoon dog		this study
	pig	KRY09282.1	[38]
	pig		[14]
Serine protease 30	fox	KRY58841.1	this study
	raccoon dog		this study
	pig	KRX47705.1	[38]
	pig		[14]
Hypothetical protein T03_8694	fox	KRY46541.1	this study
	raccoon dog		this study
	pig		[38]
Cuticlin-1	fox	KRY58751.1	this study
	raccoon dog		this study
Cuticlin-1, partial	pig	KRY01407.1	[14]
	pig	KRY57444.1	[38]
Paramyosin, partial	fox	KRY49321.1	this study
	raccoon dog		this study
	pig	KRY49322.1	[38]
Myosin-4, partial	fox	KRY50587.1	this study
	raccoon dog		this study
	pig		[38]
26S protease regulatory subunit 10B	fox	KRY59566.1	this study
	raccoon dog		this study
	pig		[38]
Adenylosuccinate lyase	fox	KRY47589.1	this study
	raccoon dog		this study
	pig		[38]
32 kDa beta-galactoside-binding lectin	fox	KRY59236.1	this study
	raccoon dog		this study
	pig		[38]
Peroxiredoxin-2, partial	fox	KRY49990.1	this study
	raccoon dog		this study
	pig	KRX46812.1	[14]
Poly-cysteine and histidine-tailed protein, partial	raccoon dog	KRY58369.1	this study
	pig	KRY11984.1	[14]
Calponin-like protein OV9M, partial	fox	KRY56610.1	this study
	raccoon dog		this study
	pig	KRX28313.1	[14]
Propionyl-CoA carboxylase alpha chain, mitochondrial	fox	KRY51819.1	this study
	raccoon dog		this study
	pig	KRZ50222.1	[14]
1,5-anhydro-D-fructose reductase	fox	KRY43899.1	this study
	raccoon dog		this study
	pig	KRY07641.1	[14]
Mitochondrial-processing peptidase subunit beta	fox	KRY53080.1	this study
	raccoon dog		this study
Mitochondrial-processing peptidase subunit beta, partial	pig	KRZ09733.1	[14]
putative histone-binding protein Caf1	fox	KRY53239.1	this study
	raccoon dog		this study
	pig	KRZ17128.1	[14]
Rab GDP dissociation inhibitor alpha	fox	KRY54650.1	this study
	raccoon dog		this study
	pig	KRY13378.1	[14]

## Data Availability

Not applicable.

## Supplementary Materials

The following supporting information can be downloaded at: https://www.mdpi.com/article/10.3390/pathogens11101155/s1, Table S1: Results of the LC-MS/MS analysis of selected gel fragments for two examined foxes: F1 and F2; Table S2: Results of the LC-MS/MS analysis of selected gel fragments for three examined raccoon dogs: R1, R2, and R3; Table S3: Gene Ontology (GO) database analysis outcomes for identified proteins from two foxes and three raccoon dogs. The proteins were categorized according to molecular function, cellular component, and biological process.
