# Supplementary material for: Immunoproteomic Analysis of Trichinella britovi Proteins Recognized by IgG Antibodies from Meat Juice of Carnivores Naturally Infected with T. britovi
Source: Pathogens. 2022 Oct 6;11(10):1155. doi: 10.3390/pathogens11101155 (PMC9612144; doi:10.3390/pathogens11101155)
Supplement: Supplementary file 1 [file pathogens-11-01155-s001.zip › Table S1.pdf]

Table S1. Results of the LC-MS/MS analysis of selected gel fragments for two examined foxes F1 and F2.

Protein name	Presence in band		Mass	Scores		Matches		Sequences		Coverage		Accession number
	F1	F2		F1	F2	F1	F2	F1	F2	F1	F2	
1,5-anhydro-D-fructose reductase	-	A	37989	-	360	-	11(11)	-	10(10)	-	25%	KRY43899.1
	B	B		70	64	1(1)	1(1)	1(1)	1(1)	2%	2%	
2,3-bisphosphoglycerate-independent phosphoglycerate mutase	-	A	59212	-	108	-	2(1)	-	2(1)	-	4%	KRY48325.1
	B	B		117	75	4(4)	1(1)	4(4)	1(1)	8%	1%	
	C	-		192	-	7(7)	-	7(7)	-	15%	-	
26S protease regulatory subunit 10B	-	B	55162	-	86	-	2(2)	-	2(2)	-	5%	KRY59566.1
26S protease regulatory subunit 4, partial	B	B	77301	194	222	3(3)	6(6)	3(3)	6(6)	7%	11%	KRY52921.1
26S protease regulatory subunit 6B	A	-	40507	90	-	2(2)	-	2(2)	-	5%	-	KRY48180.1
26S proteasome non-ATPase regulatory subunit 14	-	A	94857	-	97	-	3(3)	-	3(3)	-	4%	KRY61535.1
26S proteasome non-ATPase regulatory subunit 3, partial	C	-	59673	123	-	2(2)	-	2(2)	-	6%	-	KRY53037.1
26S proteasome non-ATPase regulatory subunit 4	B	-	42698	174	-	3(3)	-	2(2)	-	7%	-	KRY60873.1
26S proteasome non-ATPase regulatory subunit 6, partial	A	-	50950	132	-	5(5)	-	5(5)	-	11%	-	KRY50659.1
	-	B		-	76	-	1(1)	-	1(1)	-	2%	
32 kDa beta-galactoside-binding lectin	B	B	31910	160	149	4(4)	4(4)	4(4)	4(4)	16%	17%	KRY59236.1
	-	C		-	116	-	2(2)	-	2(2)	-	9%	
3-hydroxyisobutyryl-CoA hydrolase, mitochondrial, partial	-	A	40640	-	64	-	1(1)	-	1(1)	-	3%	KRY61485.1
3-ketoacyl-CoA thiolase, mitochondrial	A	A	42697	1071	225	29(28)	8(8)	17(16)	8(8)	57%	23%	KRY51064.1
	B	B		436	796	12(12)	23(23)	11(11)	14(14)	46%	40%	
	C	C		304	154	7(7)	3(3)	7(7)	3(3)	21%	8%	
40S ribosomal protein S18	B	-	26097	92	-	1(1)	-	1(1)	-	4%	-	KRY55489.1
	C	-		109	-	1(1)	-	1(1)	-	6%	-	
40S ribosomal protein S3	B	-	38314	59	-	1(1)	-	1(1)	-	3%	-	KRY60420.1
	C	-		103	-	1(1)	-	1(1)	-	2%	-	
40S ribosomal protein S5, partial	A	-	24818	68	-	1(1)	-	1(1)	-	4%	-	KRY49420.1
	C	-		60	-	1(1)	-	1(1)	-	4%	-	
40S ribosomal protein S8	B	-	72413	120	-	2(2)	-	2(2)	-	4%	-	KRY60196.1
40S ribosomal protein S9	C	-	26490	64	-	1(1)	-	1(1)	-	4%	-	KRY47012.1
4-hydroxybutyrate coenzyme A transferase, partial	A	A	53804	102	82	3(3)	1(1)	3(3)	1(1)	5%	2%	KRY56869.1
	B	B		393	66	12(12)	1(1)	12(12)	1(1)	24%	2%	
	C	-		104	-	2(2)	-	2(2)	-	4%	-	
60 kDa heat shock protein, mitochondrial	A	A	158357	364	63	10(10)	1(1)	10(10)	1(1)	8%	0%	KRY51676.1
	B	B		454	119	13(12)	4(4)	13(12)	4(4)	10%	3%	
	C	C		1224	284	26(26)	7(6)	19(19)	7(6)	18%	6%	
60 kDa SS-A/Ro ribonucleoprotein, partial	C	-	69458	142	-	3(3)	-	3(3)	-	5%	-	KRY47029.1
60S acidic ribosomal protein P0	B	-	32092	142	-	2(2)	-	2(2)	-	9%	-	KRY54528.1
	C	C		95	103	2(2)	1(1)	2(2)	1(1)	8%	5%	
60S ribosomal protein L17, partial	B	-	146568	69	-	1(1)	-	1(1)	-	0%	-	KRY50982.1
60S ribosomal protein L18a	B	-	22864	82	-	2(2)	-	2(2)	-	9%	-	KRY60168.1
60S ribosomal protein L21, partial	B	-	19610	57	-	1(1)	-	1(1)	-	9%	-	KRY48279.1

Protein name	Presence in band		Mass	Scores		Matches		Sequences		Coverage		Accession number
60S ribosomal protein L3	A	-	34644	90	-	4(4)	-	3(3)	-	9%	-	KRY46416.1
	B	-		70	-	2(2)	-	2(2)	-	5%	-	
	C	-		70	-	1(1)	-	1(1)	-	3%	-	
60S ribosomal protein L4	-	A	31108	-	76	-	1(1)	-	1(1)	-	6%	KRY61057.1
	C	-		68	-	2(1)	-	2(1)	-	5%	-	
60S ribosomal protein L4-B, partial	C	-	43559	68	-	2(1)	-	2(1)	-	4%	-	KRY61056.1
Acetyl-CoA acetyltransferase, mitochondrial	A	-	43775	59	-	1(0)	-	1(0)	-	2%	-	KRY60246.1
Actin-interacting protein 1	B	-	71319	122	-	2(2)	-	2(2)	-	4%	-	KRY51005.1
	C	C		390	126	13(13)	2(2)	12(12)	2(2)	23%	3%	
Actin-like protein 6A	B	-	120874	60	-	1(1)	-	1(1)	-	0%	-	KRY56723.1
Actin-related protein 2, partial	-	B	155475	-	75	-	1(1)	-	1(1)	-	0%	KRY53921.1
Actin-related protein 2/3 complex subunit 2	A	-	132079	158	-	6(6)	-	6(6)	-	5%	-	KRY56438.1
	B	B		104	114	1(1)	3(3)	1(1)	3(3)	1%	2%	
Actin-related protein 3	B	-	128876	61	-	1(1)	-	1(1)	-	1%	-	KRY47903.1
Acyl-CoA synthetase family member 2, mitochondrial, partial	-	C	67058	-	63	-	1(1)	-	1(1)	-	2%	KRY51298.1
Adducin-related protein 1	A	-	80242	63	-	1(1)	-	1(1)	-	1%	-	KRY47021.1
	B	-		177	-	4(4)	-	4(4)	-	6%	-	
	C	C		107	67	2(2)	1(1)	2(2)	1(1)	3%	1%	
Adenylosuccinate lyase	A	-	185820	129	-	1(1)	-	1(1)	-	0%	-	KRY47589.1
	B	B		1107	70	32(32)	1(1)	20(20)	1(1)	12%	0%	
	-	C		-	59	-	1(1)	-	1(1)	-	0%	
Adenylosuccinate synthetase, partial	A	-	50894	101	-	2(2)	-	2(2)	-	4%	-	KRY50054.1
	B	B		220	126	5(5)	2(2)	5(5)	2(2)	28%	4%	
	C	-		70	-	1(1)	-	1(1)	-	2%	-	
Adenylyl cyclase-associated protein 2	B	-	88901	136	-	3(3)	-	3(3)	-	5%	-	KRY58311.1
ADP-ribose pyrophosphatase, mitochondrial	A	-	55423	66	-	1(1)	-	1(1)	-	2%	-	KRY57225.1
	B	-	58846	102	-	1(1)	-	1(1)	-	2%	-	KRY48334.1
	-	C		-	95	-	1(1)	-	1(1)	-	2%	
	-	A	37863	-	77	-	2(2)	-	2(2)	-	5%	KRY49532.1
	-	B	55835	-	118	-	2(2)	-	2(2)	-	5%	KRY50360.1
Alanine aminotransferase 2	A	-	63694	91	-	1(1)	-	1(1)	-	2%	-	KRY55706.1
	B	B		183	83	3(2)	1(1)	3(2)	1(1)	8%	2%	
	C	C		97	157	1(1)	3(3)	1(1)	3(3)	2%	5%	
Aldehyde dehydrogenase, mitochondrial	C	C	94777	102	244	1(1)	7(7)	1(1)	7(7)	2%	11%	KRY52913.1
Alpha-L-fucosidase	A	-	55241	73	-	2(2)	-	2(2)	-	4%	-	KRY54685.1
	B	B		101	69	3(3)	2(1)	3(3)	2(1)	6%	4%	
Aminomethyltransferase, mitochondrial	-	B	100050	-	111	-	3(3)	-	3(3)	-	3%	KRY54143.1
Aminopeptidase N	-	B	109166	-	69	-	1(1)	-	1(1)	-	1%	KRY54999.1
Ankyrin-2, partial	B	-	821521	70	-	1(1)	-	1(1)	-	0%	-	KRY56996.1
AP-2 complex subunit alpha-2	C	-	106493	80	-	1(1)	-	1(1)	-	1%	-	KRY60602.1
AP-2 complex subunit beta	C	-	106080	74	-	2(2)	-	2(2)	-	2%	-	KRY52390.1
Arrestin domain-containing protein 3, partial	B	-	64593	111	-	2(2)	-	2(2)	-	5%	-	KRY54875.1

Protein name	Presence in band		Mass	Scores		Matches		Sequences		Coverage		Accession number
Aspartate aminotransferase, mitochondrial, partial	A	-	47470	110	-	2(2)	-	2(2)	-	5%	-	KRY49869.1
	C	-		76	-	1(1)	-	1(1)	-	2%	-	
	-	B		-	115	-	1(1)	-	1(1)	-	3%	
Aspartate--tRNA ligase, cytoplasmic	B	-	57949	165	-	3(3)	-	3(3)	-	7%	-	KRY49144.1
	C	C		86	163	1(1)	5(5)	1(1)	5(5)	1%	11%	
Aspartyl aminopeptidase	A	-	52372	67	-	1(1)	-	1(1)	-	2%	-	KRY46829.1
	B	-		382	-	8(8)	-	7(7)	-	20%	-	
Atlastin-2	C	-	68364	212	-	4(4)	-	4(4)	-	10%	-	KRY58631.1
ATP synthase subunit alpha, mitochondrial, partial	A	A	64231	71	71	2(2)	1(1)	2(2)	1(1)	4%	1%	KRY49394.1
	B	B		197	125	4(4)	2(2)	4(4)	2(2)	8%	4%	
	C	C		123	308	2(2)	11(11)	2(2)	9(9)	5%	14%	
ATPase asna1	-	A	40013	-	101	-	2(2)	-	2(2)	-	5%	KRY53402.1
ATP-dependent RNA helicase DDX19A, partial	B	-	114761	89	-	2(2)	-	2(2)	-	2%	-	KRY47762.1
BRCA1-A complex subunit BRE, partial	B	-	52274	68	-	1(1)	-	1(1)	-	2%	-	KRY48007.1
Breast cancer anti-estrogen resistance protein 1, partial	A	A	131710	76	91	1(1)	1(1)	1(1)	1(1)	0%	0%	KRY51653.1
	-	B		-	77	-	1(1)	-	1(1)	-	0%	
C-1-tetrahydrofolate synthase, cytoplasmic	A	-	125620	69	-	1(1)	-	1(1)	-	0%	-	KRY60034.1
Calpain clp-1	A	-	150994	85	-	1(1)	-	1(1)	-	0%	-	KRY60775.1
	B	-		106	-	2(2)	-	2(2)	-	1%	-	
	C	C		208	66	11(10)	1(1)	9(9)	1(1)	7%	0%	
Calponin -like protein OV9M, partial	A	-	63579	95	-	1(1)	-	1(1)	-	2%	-	KRY56610.1
	B	-		78	-	1(1)	-	1(1)	-	2%	-	
	C	-		211	-	5(5)	-	5(5)	-	12%	-	
	-	B	54494	-	92	-	1(1)	-	1(1)	-	3%	KRY56611.1
	-	C		-	106	-	2(2)	-	2(2)	-	6%	
Calreticulin, partial	A	-	51118	208	-	4(4)	-	4(4)	-	9%	-	KRY53845.1
	B	B		734	153	15(15)	4(4)	11(11)	4(4)	40%	13%	
cAMP-dependent protein kinase catalytic subunit alpha, partial	A	-	52893	86	-	2(2)	-	2(2)	-	5%	-	KRY51804.1
	B	-		66	-	1(1)	-	1(1)	-	1%	-	
cAMP-dependent protein kinase regulatory subunit	A	A	47292	94	76	1(1)	1(1)	1(1)	1(1)	3%	3%	KRY58719.1
	B	B		119	106	1(1)	1(1)	1(1)	1(1)	3%	3%	
	-	C		-	118	-	1(1)	-	1(1)	-	3%	
CDK5RAP3-like protein	-	C	57510	-	80	-	2(2)	-	2(2)	-	5%	KRY58531.1
Chitobiosyldiphosphodolichol beta-mannosyltransferase, partial	B	-	44025	99	-	1(1)	-	1(1)	-	5%	-	KRY58507.1
Chondroadherin	-	C	54970	-	77	-	1(1)	-	1(1)	-	2%	KRY58748.1
Chymotrypsin-like elastase family member 2A, partial	A	-	46401	410	-	12(12)	-	10(10)	-	33%	-	KRY59723.1
	B	B		530	381	18(18)	12(12)	16(16)	10(10)	53%	33%	
Chymotrypsinogen B	C	-	141902	126	-	3(3)	-	3(3)	-	3%	-	KRY53598.1
Cleavage and polyadenylation specificity factor subunit 2	C	C	142890	363	380	12(12)	12(12)	11(11)	11(11)	9%	12%	KRY53654.1
Cleavage stimulation factor subunit 3	B	-	125680	60	-	1(1)	-	1(1)	-	0%	-	KRY56722.1
Coatomer subunit delta	C	-	63168	137	-	4(4)	-	4(4)	-	7%	-	KRY53209.1
Coronin-like protein cor-1	C	-	164005	113	-	2(2)	-	2(2)	-	1%	-	KRY60446.1

Protein name	Presence in band		Mass	Scores		Matches		Sequences		Coverage		Accession number
Cuticlin-1	A	-	64300	69	-	1(1)	-	1(1)	-	1%	-	KRY58751.1
Cysteine desulfurase, mitochondrial	B	-	49103	131	-	2(2)	-	2(2)	-	6%	-	KRY49825.1
Cytochrome b-c1 complex subunit 2, mitochondrial, partial	A	-	54732	174	-	4(4)	-	4(4)	-	9%	-	KRY49815.1
	B	B		203	109	3(3)	1(1)	3(3)	1(1)	7%	3%	
Cytosol aminopeptidase	A	A	57910	78	78	1(1)	1(1)	1(1)	1(1)	1%	1%	KRY54953.1
	B	-		195	-	5(5)	-	5(5)	-	10%	-	
	C	C		154	826	3(3)	19(19)	3(3)	9(9)	6%	20%	
Cytosolic non-specific dipeptidase	B	-	58585	149	-	3(3)	-	3(3)	-	8%	-	KRY53197.1
Cytosolic purine 5'-nucleotidase	C	-	124481	92	-	1(1)	-	1(1)	-	0%	-	KRY47033.1
Delta-1-pyrroline-5-carboxylate dehydrogenase, mitochondrial, partial	C	C	132746	100	379	1(1)	8(8)	1(1)	7(7)	1%	7%	KRY58107.1
Deoxyribonuclease-2-alpha	-	A	116176	-	261	-	8(8)	-	7(7)	-	9%	KRY53318.1
	C	C		331	321	7(7)	9(9)	7(7)	8(8)	9%	10%	
Dihydrolipoyl dehydrogenase, mitochondrial	A	-	58994	93	-	1(1)	-	1(1)	-	2%	-	KRY53824.1
	B	B		196	62	7(7)	1(1)	6(6)	1(1)	13%	2%	
	-	C	55073	-	86	-	2(2)	-	2(2)	-	4%	KRY53825.1
Dihydrolipoyllysine-residue acetyltransferase component of pyruvate dehydrogenase complex, mitochondrial	B	B	57120	198	114	6(5)	1(1)	6(5)	1(1)	12%	2%	KRY46167.1
	C	-		106	-	1(1)	-	1(1)	-	2%	-	
Dihydrolipoyllysine-residue succinyltransferase component of 2-oxoglutarate dehydrogenase complex, mitochondrial	B	-	59790	106	-	3(3)	-	3(3)	-	5%	-	KRY53977.1
Dihydropyrimidinase-related protein 3	A	-	86548	62	-	1(1)	-	1(1)	-	1%	-	KRY48683.1
	B	-		131	-	2(2)	-	2(2)	-	2%	-	
	C	C		99	129	2(2)	2(2)	2(2)	2(2)	2%	2%	
Diphthine--ammonia ligase, partial	A	-	149815	67	-	1(1)	-	1(1)	-	0%	-	KRY47980.1
	B	B		110	67	3(3)	1(1)	3(3)	1(1)	2%	0%	
DnaJ -like protein dnj-20	A	-	45223	114	-	2(2)	-	2(2)	-	7%	-	KRY45252.1
	-	B		-	106	-	2(2)	-	2(2)	-	7%	
DnaJ -like protein subfamily C member 3, partial	-	C	57419	-	164	-	4(4)	-	4(4)	-	8%	KRY53630.1
Dynein heavy chain, cytoplasmic	A	B	569784	141	125	4(4)	3(3)	4(4)	3(3)	0%	0%	KRY59545.1
Elongation factor 1-alpha	-	A	54609	-	153	-	4(4)	-	3(3)	-	6%	KRY54798.1
	-	B		-	268	-	7(7)	-	6(6)	-	20%	
	-	C		-	245	-	7(7)	-	6(6)	-	15%	
Elongation factor 1-gamma, partial	B	-	52493	179	-	6(5)	-	6(5)	-	12%	-	KRY58663.1
Elongation factor G, mitochondrial	C	C	101076	64	79	1(1)	1(1)	1(1)	1(1)	1%	1%	KRY55228.1
Elongation factor Ts, mitochondrial	-	B	71640	-	65	-	1(1)	-	1(1)	-	1%	KRY51161.1
Elongator complex protein 3	C	-	65381	75	-	1(1)	-	1(1)	-	1%	-	KRY54549.1
Endoplasmic reticulum resident protein 44	A	-	50147	96	-	1(1)	-	1(1)	-	2%	-	KRY50742.1
	B	B		236	72	5(5)	1(1)	4(4)	1(1)	13%	2%	
	C	-		64	-	1(1)	-	1(1)	-	2%	-	
Endoplasmin, partial	A	A	95730	262	91	7(7)	1(1)	6(6)	1(1)	9%	1%	KRY54651.1
	-	B		-	241	-	6(6)	-	5(5)	-	7%	
Enoyl-CoA delta isomerase 2, mitochondrial	A	-	47790	82	-	1(1)	-	1(1)	-	3%	-	KRY48829.1

Protein name	Presence in band		Mass	Scores		Matches		Sequences		Coverage		Accession number
Enoyl-CoA hydratase, mitochondrial	A	-	34086	82	-	1(1)	-	1(1)	-	4%	-	KRY60485.1
	B	-		121	-	2(2)	-	2(2)	-	7%	-	
Epoxide hydrolase 1	B	-	95423	73	-	1(1)	-	1(1)	-	1%	-	KRY56031.1
Eukaryotic initiation factor 4A, partial	A	A	252102	173	82	4(4)	1(1)	3(3)	1(1)	1%	0%	KRY53637.1
	-	B		-	138	-	2(2)	-	2(2)	-	1%	
	C	C		69	64	1(1)	1(1)	1(1)	1(1)	0%	0%	
Eukaryotic initiation factor 4A-III	B	-	126738	165	-	7(5)	-	7(5)	-	6%	-	KRY49327.1
Eukaryotic initiation factor 4A-III	-	C	123600	-	76	-	1(1)	-	1(1)	-	1%	KRY49326.1
Eukaryotic peptide chain release factor subunit 1	B	-	49470	87	-	1(1)	-	1(1)	-	2%	-	KRY49983.1
Eukaryotic translation initiation factor 3 subunit L	C	-	65995	118	-	6(6)	-	6(6)	-	10%	-	KRY53932.1
Extended synaptotagmin-2, partial	C	-	205677	111	-	4(4)	-	4(4)	-	2%	-	KRY50391.1
Far upstream element-binding protein 2	B	B	75769	273	71	10(10)	1(1)	8(8)	1(1)	12%	1%	KRY57083.1
	C	-		93	-	3(3)	-	3(3)	-	4%	-	
Filamin-A, partial	-	B	259917	-	84	-	1(1)	-	1(1)	-	0%	KRY59624.1
Fragile X mental retardation protein 1 -like protein B	C	-	64707	130	-	4(4)	-	4(4)	-	6%	-	KRY58060.1
Fructose-bisphosphate aldolase 1, partial	A	A	44597	901	321	17(17)	8(8)	10(10)	6(6)	38%	18%	KRY52953.1
	-	B		-	669	-	13(13)	-	9(9)	-	34%	
	C	C		228	126	4(4)	1(1)	4(4)	1(1)	13%	2%	
GDP-fucose protein O-fucosyltransferase 1	A	B	55623	65	70	1(1)	1(1)	1(1)	1(1)	2%	2%	KRY61128.1
GDP-fucose protein O-fucosyltransferase 2	-	C	67028	-	66	-	1(1)	-	1(1)	-	2%	KRY57885.1
Geranylgeranyl transferase type-2 subunit alpha	C	-	65451	60	-	1(1)	-	1(1)	-	1%	-	KRY54409.1
Glioma pathogenesis-related protein 1	A	-	54399	103	-	1(1)	-	1(1)	-	3%	-	KRY45995.1
Glucose-6-phosphate 1-dehydrogenase	C	-	61532	116	-	4(4)	-	4(4)	-	8%	-	KRY54351.1
Glucose-6-phosphate isomerase	B	-	65882	187	-	3(3)	-	3(3)	-	7%	-	KRY50483.1
	C	C		462	193	13(11)	4(4)	13(11)	4(4)	26%	8%	
Glucosylceramidase	C	-	61978	214	-	7(7)	-	5(5)	-	11%	-	KRY50060.1
Glutamate dehydrogenase 2, mitochondrial	A	A	56781	95	78	2(2)	1(1)	2(2)	1(1)	4%	2%	KRY47480.1
Glutathione synthetase, partial	-	C	57435	-	68	-	1(1)	-	1(1)	-	1%	KRY45971.1
Glutathione-S-transferase	A	-	24198	71	-	1(1)	-	1(1)	-	5%	-	ABA42916.1
	C	-		72	-	1(1)	-	1(1)	-	5%	-	
Glyceraldehyde-3-phosphate dehydrogenase	A	A	37666	142	497	5(5)	18(18)	5(5)	12(12)	16%	53%	KRY59635.1
	B	B		199	123	3(2)	2(2)	3(2)	2(2)	11%	6%	
	C	C		325	160	6(6)	3(3)	6(6)	3(3)	24%	13%	
Glycogen phosphorylase, partial	A	-	175168	85	-	1(1)	-	1(1)	-	0%	-	KRY47089.1
	B	B		117	84	1(1)	2(1)	1(1)	2(1)	0%	1%	
	-	C		-	130	-	3(3)	-	3(3)	-	2%	
Glycogenin-1	A	A	41089	135	79	4(4)	2(1)	3(3)	2(1)	10%	5%	KRY61196.1
	B	B		118	106	2(2)	3(3)	2(2)	3(3)	7%	10%	
Guanine nucleotide-binding protein subunit beta-2-like 1	B	-	39904	86	-	1(1)	-	1(1)	-	3%	-	KRY55875.1
	C	-		84	-	1(1)	-	1(1)	-	3%	-	
Heat shock 70 kDa protein 13	C	-	50433	62	-	1(1)	-	1(1)	-	3%	-	KRY49991.1
Heat shock 70 kDa protein 4L	B	-	101361	136	-	2(2)	-	2(2)	-	3%	-	KRY51771.1

Protein name	Presence in band		Mass	Scores		Matches		Sequences		Coverage		Accession number
	C	C		412	98	11(11)	1(1)	11(11)	1(1)	17%	1%	
Heat shock cognate 71 kDa protein, partial	A	A	131529	548	206	18(17)	4(4)	15(14)	4(4)	15%	4%	KRY58599.1
	B	B		278	360	5(5)	13(13)	5(5)	12(12)	5%	13%	
	C	C		918	319	26(26)	10(9)	20(20)	10(9)	21%	10%	
heat shock protein 70	-	A	71860	-	206	-	4(4)	-	4(4)	-	7%	CAA73574.1
Heat shock protein 83, partial	A	A	91707	109	91	5(5)	1(1)	5(5)	1(1)	5%	1%	KRY48983.1
	B	B		593	112	24(23)	3(3)	19(18)	3(3)	22%	4%	
	C	C		264	194	10(9)	6(6)	9(8)	6(6)	12%	8%	
Heterogeneous nuclear ribonucleoprotein F, partial	-	C	54653	-	88	-	1(1)	-	1(1)	-	2%	KRY60656.1
Hexokinase	B	-	56783	567	-	14(14)	-	10(10)	-	31%	-	KRY57386.1
Hexokinase type 2, partial	B	-	52657	232	-	3(3)	-	3(3)	-	8%	-	KRY53884.1
Histidine--tRNA ligase, cytoplasmic	-	C	60198	-	93	-	1(1)	-	1(1)	-	2%	KRY60106.1
Histone deacetylase Rpd3	-	C	55626	-	105	-	2(2)	-	2(2)	-	4%	KRY47841.1
Histone-lysine N-methyltransferase EHMT2, partial	A	B	40953	75	104	1(1)	1(1)	1(1)	1(1)	3%	4%	KRY52481.1
Hsc70-interacting protein, partial	-	C	45031	-	80	-	1(1)	-	1(1)	-	3%	KRY60345.1
hypothetical protein T03_11006	-	C	163737	-	256	-	6(6)	-	6(6)	-	5%	KRY56142.1
hypothetical protein T03_13721	C	-	16489	61	-	1(1)	-	1(1)	-	8%	-	KRY56557.1
hypothetical protein T03_13827	C	C	13483	81	73	1(1)	1(1)	1(1)	1(1)	10%	9%	KRY56487.1
hypothetical protein T03_14678	-	C	55770	-	454	-	11(11)	-	6(6)	-	17%	KRY57784.1
hypothetical protein T03_17187	A	A	46783	743	175	14(14)	4(3)	10(10)	4(3)	36%	10%	KRY50178.1
	B	B		1214	431	25(24)	9(9)	17(16)	8(8)	52%	27%	
	-	C		-	59	-	1(1)	-	1(1)	-	2%	
hypothetical protein T03_17805, partial	C	-	18630	62	-	1(1)	-	1(1)	-	6%	-	KRY53056.1
hypothetical protein T03_18095	B	-	49475	64	-	1(1)	-	1(1)	-	2%	-	KRY58400.1
hypothetical protein T03_4197	B	-	88736	73	-	1(1)	-	1(1)	-	1%	-	KRY53644.1
	C	-		238	-	5(5)	-	4(4)	-	7%	-	
hypothetical protein T03_539	A	A	55965	603	229	15(15)	7(7)	9(9)	5(5)	22%	9%	KRY55455.1
	-	B		-	468	-	13(13)	-	9(9)	-	19%	
hypothetical protein T03_5579, partial	-	A	40699	-	70	-	1(1)	-	1(1)	-	2%	KRY49176.1
hypothetical protein T03_6875	C	-	13215	61	-	1(1)	-	1(1)	-	10%	-	KRY51561.1
hypothetical protein T03_7457	C	-	49230	201	-	4(4)	-	4(4)	-	11%	-	KRY48668.1
hypothetical protein T03_7459	B	-	13934	75	-	1(1)	-	1(1)	-	7%	-	KRY60639.1
	C	C		124	64	1(1)	1(1)	1(1)	1(1)	13%	13%	
hypothetical protein T03_7883	-	B	117012	-	66	-	1(1)	-	1(1)	-	1%	KRY46989.1
	-	C		-	81	-	2(2)	-	2(2)	-	2%	
hypothetical protein T03_7939	-	A	64129	-	300	-	9(9)	-	8(8)	-	18%	KRY48964.1
hypothetical protein T03_8666, partial	A	B	29868	61	86	1(0)	3(2)	1(0)	3(2)	3%	13%	KRY58917.1
	B	C	55679	412	227	8(8)	6(6)	6(6)	5(5)	19%	13%	KRY58914.1
hypothetical protein T03_8694	A	A	40952	110	313	5(4)	11(11)	5(4)	11(11)	15%	32%	KRY46541.1
	B	B		70	78	1(1)	2(1)	1(1)	2(1)	3%	5%	
	C	C		65	64	2(2)	1(1)	2(2)	1(1)	5%	3%	
hypothetical protein T03_9489	C	C	19940	72	82	2(2)	1(1)	2(2)	1(1)	11%	5%	KRY46491.1

Protein name	Presence in band		Mass	Scores		Matches		Sequences		Coverage		Accession number
Hypoxia up-regulated protein 1, partial	C	-	108762	78	-	1(1)	-	1(1)	-	1%	-	KRY49848.1
Ig-like and fibronectin type-III domain-containing protein C25G4.10	C	C	179761	70	77	1(1)	2(2)	1(1)	2(2)	0%	1%	KRY58616.1
Importin subunit alpha-4, partial	-	C	151048	-	61	-	1(1)	-	1(1)	-	0%	KRY51490.1
Innexin unc-9	-	A	48936	-	82	-	1(1)	-	1(1)	-	2%	KRY59455.1
Innexin-12	B	-	55187	120	-	3(3)	-	3(3)	-	7%	-	KRY59456.1
Intermediate filament protein B, partial	A	-	71689	69	-	1(1)	-	1(1)	-	1%	-	KRY59373.1
	B	B		397	75	16(14)	1(1)	15(13)	1(1)	24%	1%	
	C	C		390	289	17(17)	11(11)	15(15)	10(10)	24%	17%	
Intermediate filament protein ifa-1	A	A	73429	207	120	9(8)	6(4)	8(7)	6(4)	12%	8%	KRY45949.1
	B	B		791	120	34(33)	5(4)	29(28)	5(4)	37%	6%	
	C	C		675	518	25(24)	26(25)	21(21)	23(22)	32%	30%	
Intramembrane protease 2	-	C	52321	-	71	-	1(1)	-	1(1)	-	3%	KRY54667.1
Iron-sulfur cluster assembly enzyme ISCU, mitochondrial	C	-	202951	83	-	1(1)	-	1(1)	-	0%	-	KRY49130.1
Isopentenyl-diphosphate Delta-isomerase II, partial	C	-	41386	68	-	1(1)	-	1(1)	-	2%	-	KRY59814.1
J5 protein, partial	-	A	41174	-	94	-	1(1)	-	1(1)	-	2%	AAD42227.1
KDEL motif-containing protein 2, partial			46318	105	-	2(2)	-	2(2)	-	6%	-	KRY44318.1
KH domain-containing, RNA-binding, signal transduction-associated protein 2	C	-	49642	97	-	1(1)	-	1(1)	-	2%	-	KRY48053.1
Kynurenine--oxoglutarate transaminase 3	A	B	60100	184	97	4(4)	1(1)	4(4)	1(1)	9%	2%	KRY54139.1
L-2-hydroxyglutarate dehydrogenase, mitochondrial	A	B	47961	120	116	2(2)	3(3)	2(2)	3(3)	5%	9%	KRY56323.1
LanC-like protein 2	-	A	87536	-	97	-	1(1)	-	1(1)	-	1%	KRY60641.1
LETM1 and EF-hand domain-containing protein 1, mitochondrial	-	B	118848	-	87	-	1(1)	-	1(1)	-	1%	KRY56766.1
Leukocyte elastase inhibitor C, partial	A	-	43705	104	-	2(2)	-	2(2)	-	4%	-	KRY55578.1
	B	B		198	74	7(7)	1(1)	6(6)	1(1)	18%	2%	
Lipase maturation factor 2	C	-	77441	128	-	2(2)	-	1(1)	-	1%	-	KRY59894.1
L-lactate dehydrogenase	C	-	44144	80	-	1(1)	-	1(1)	-	3%	-	KRY58174.1
L-threonine dehydratase catabolic TdcB	-	B	45107	-	73	-	1(1)	-	1(1)	-	2%	KRY56516.1
Lupus La -like protein A, partial	B	-	120320	143	-	2(2)	-	2(2)	-	2%	-	KRY45724.1
	C	-		127	-	2(2)	-	2(2)	-	2%	-	
Lysosomal aspartic protease, partial	A	-	46676	212	-	3(3)	-	3(3)	-	13%	-	KRY49432.1
	B	B		132	257	2(2)	3(3)	2(2)	3(3)	10%	14%	
MAGUK p55 subfamily member 2	C	-	62788	61	-	1(1)	-	1(1)	-	2%	-	KRY57541.1
Malate dehydrogenase, cytoplasmic	A	-	36616	99	-	2(1)	-	2(1)	-	6%	-	KRY57329.1
	B	-		68	-	2(2)	-	2(2)	-	4%	-	
	C	C		224	126	5(5)	3(3)	5(5)	3(3)	17%	12%	
Malate dehydrogenase, partial	-	A	43114	-	156	-	3(3)	-	2(2)	-	8%	KRY47491.1
Mannose-6-phosphate isomerase, partial	A	-	47870	187	-	7(7)	-	6(6)	-	19%	-	KRY55073.1
	-	B		-	99	-	3(2)	-	3(2)	-	10%	
Mediator of RNA polymerase II transcription subunit 23	B	-	258401	96	-	3(2)	-	3(2)	-	2%	-	KRY54660.1
	-	C		-	235	-	8(8)	-	7(7)	-	3%	
Medium-chain specific acyl-CoA dehydrogenase, mitochondrial, partial	A	-	46251	188	-	4(4)	-	4(4)	-	10%	-	KRY52707.1
	-	B		-	148	-	3(3)	-	3(3)	-	7%	

Protein name	Presence in band		Mass	Scores		Matches		Sequences		Coverage		Accession number
Methionine aminopeptidase 1, partial	A	-	45052	139	-	5(4)	-	5(4)	-	13%	-	KRY48197.1
Methylmalonyl-CoA mutase, mitochondrial	C	-	96758	70	-	1(1)	-	1(1)	-	1%	-	KRY55816.1
Mevalonate kinase, partial	A	-	49923	65	-	1(1)	-	1(1)	-	2%	-	KRY48327.1
Mitochondrial EF-Tu1 precursor	A	-	53698	63	-	1(1)	-	1(1)	-	2%	-	BAF32575.1
	B	-		198	-	7(7)	-	7(7)	-	18%	-	
Mitochondrial inner membrane protein	C	-	51259	137	-	2(2)	-	2(2)	-	4%	-	KRY52343.1
Mitochondrial ornithine transporter 1	B	-	131282	81	-	1(1)	-	1(1)	-	0%	-	KRY52596.1
Mitochondrial succinyl-CoA ligase subunit beta -like protein	A	A	47920	272	82	8(8)	1(1)	7(7)	1(1)	20%	2%	KRY60682.1
	B	B		97	180	1(1)	5(5)	1(1)	5(5)	2%	12%	
Mitochondrial-processing peptidase subunit alpha	A	B	114673	151	118	5(5)	2(2)	5(5)	2(2)	5%	2%	KRY50357.1
	-	C	62700	-	117	-	4(4)	-	3(3)	-	6%	KRY50359.1
Mitochondrial-processing peptidase subunit beta	B	B	95488	469	68	13(13)	1(1)	12(12)	1(1)	18%	1%	KRY53080.1
Motochondrial isocitrate dehydrogenase, partial	A	-	91260	72	-	1(1)	-	1(1)	-	1%	-	KRY60163.1
	-	B		-	82	-	1(1)	-	1(1)	-	1%	
mRNA-capping enzyme	C	-	63559	79	-	1(1)	-	1(1)	-	1%	-	KRY57625.1
Myophilin	A	-	29149	95	-	1(1)	-	1(1)	-	4%	-	KRY53866.1
	B	-		106	-	1(1)	-	1(1)	-	4%	-	
	C	C		127	101	2(2)	1(1)	2(2)	1(1)	9%	4%	
Myosin regulatory light chain 1, partial	C	-	21489	82	-	1(1)	-	1(1)	-	7%	-	KRY55173.1
Myosin-4, partial	A	-	233904	124	-	4(4)	-	4(4)	-	2%	-	KRY50587.1
	B	-		90	-	3(2)	-	3(2)	-	1%	-	
	C	-		438	-	13(13)	-	11(11)	-	6%	-	
NAD kinase 2, mitochondrial, partial	B	-	53835	101	-	1(1)	-	1(1)	-	2%	-	KRY54079.1
NADH dehydrogenase [ubiquinone] 1 alpha subcomplex subunit 10, mitochondrial, partial	B	-	52610	93	-	1(1)	-	1(1)	-	2%	-	KRY51158.1
NADH dehydrogenase [ubiquinone] 1 alpha subcomplex subunit 9, mitochondrial, partial	A	-	51279	70	-	1(1)	-	1(1)	-	2%	-	KRY46213.1
NADP-dependent malic enzyme, mitochondrial, partial	B	-	136914	84	-	1(1)	-	1(1)	-	1%	-	KRY59664.1
	C	C		1136	178	31(31)	6(6)	22(22)	6(6)	21%	4%	
NADP-dependent malic enzyme, partial	C	-	67015	116	-	3(3)	-	3(3)	-	5%	-	KRY50082.1
Nedd8-activating enzyme E1 regulatory subunit	B	-	81417	92	-	1(1)	-	1(1)	-	1%	-	KRY49521.1
Neuroglian	C	-	151761	245	-	5(5)	-	5(5)	-	5%	-	KRY57340.1
Nicotinamide phosphoribosyltransferase, partial	A	-	54479	68	-	1(1)	-	1(1)	-	2%	-	KRY61111.1
	B	-		86	-	1(1)	-	1(1)	-	2%	-	
NSFL1 cofactor p47	A	-	96155	76	-	1(1)	-	1(1)	-	1%	-	KRY55488.1
	-	B		-	79	-	1(1)	-	1(1)	-	1%	
Nuclear migration protein nudC, partial	-	A	36924	-	74	-	1(1)	-	1(1)	-	2%	KRY49193.1
Nuclease domain-containing protein	C	-	105353	94	-	1(1)	-	1(1)	-	1%	-	KRY53536.1
Nucleobindin-2	C	-	69745	75	-	1(1)	-	1(1)	-	2%	-	KRY57471.1
Nucleolar GTP-binding protein 1	C	C	217720	122	82	3(3)	1(1)	2(2)	1(1)	1%	0%	KRY59079.1
Nucleolysin TIAR	-	A	41098	-	78	-	1(1)	-	1(1)	-	3%	KRY56682.1
Nucleosome assembly protein 1-like 4	B	-	42769	190	-	6(5)	-	6(5)	-	19%	-	KRY56244.1

Protein name	Presence in band		Mass	Scores		Matches		Sequences		Coverage		Accession number
NudC domain-containing protein 1	C	-	70408	147	-	6(5)	-	6(5)	-	9%	-	KRY59809.1
Obg-like ATPase 1	A		46859	130	-	3(3)	-	3(3)	-	7%	-	KRY54762.1
	-	B		-	200	-	5(5)	-	5(5)	-	16%	
Oligosaccharyltransferase complex subunit OSTC	A	-	35534	71	-	1(1)	-	1(1)	-	4%	-	KRY48322.1
	B	-		73	-	1(1)	-	1(1)	-	4%	-	
	C	-		86	-	1(1)	-	1(1)	-	4%	-	
Ornithine decarboxylase, partial	-	B	168568	-	104	-	1(1)	-	1(1)	-	1%	KRY52483.1
Paramyosin, partial	-	A	96956	-	74	-	1(1)	-	1(1)	-	1%	KRY49321.1
	C	-		114	-	3(3)	-	3(3)	-	3%	-	
Peroxiredoxin-2, partial	A	-	23367	186	-	3(3)	-	3(3)	-	18%	-	KRY49990.1
	-	B		-	165	-	3(3)	-	3(3)	-	18%	
Phosphate carrier protein, mitochondrial, partial	-	A	69926	-	92	-	1(1)	-	1(1)	-	2%	KRY57035.1
	B	-		94	-	2(2)	-	2(2)	-	3%	-	
	-	C		-	88	-	1(1)	-	1(1)	-	2%	
Phosphoacetylglucosamine mutase	A	-	76610	72	-	1(1)	-	1(1)	-	1%	-	KRY55926.1
	B	-		84	-	1(1)	-	1(1)	-	1%	-	
	-	C		-	94	-	1(1)	-	1(1)	-	1%	
Phosphoglucomutase-1	B	-	62761	89	-	2(2)	-	2(2)	-	3%	-	KRY58984.1
	C	C		335	92	10(9)	3(3)	8(7)	3(3)	17%	6%	
Phosphoinositide 3-kinase regulatory subunit 4, partial	-	A	249279	-	82	-	1(1)	-	1(1)	-	0%	KRY53638.1
	-	B		-	138	-	2(2)	-	2(2)	-	1%	
	C	C		69	64	1(1)	1(1)	1(1)	1(1)	0%	0%	
Poly(U)-specific endoribonuclease -like protein, partial	-	B	61077	-	65	-	1(1)	-	1(1)	-	1%	KRY51160.1
Polyadenylate-binding protein	-	B	63357	-	97	-	1(1)	-	1(1)	-	3%	KRY49582.1
	-	C		-	130	-	3(3)	-	3(3)	-	6%	
Polyadenylate-binding protein 1	A	-	89404	151	-	4(4)	-	4(4)	-	5%	-	KRY49579.1
	B	-		228	-	5(5)	-	5(5)	-	8%	-	
	C	C		165	130	3(3)	3(3)	3(3)	3(3)	5%	4%	
Polyribonucleotide nucleotidyltransferase 1, mitochondrial	C	C	137745	113	77	3(3)	1(1)	3(3)	1(1)	2%	1%	KRY55114.1
Polyubiquitin	-	A	71793	-	62	-	1(0)	-	1(0)	-	1%	KRY46475.1
	B	B		76	108	2(1)	1(1)	2(1)	1(1)	2%	2%	
	C	C		80	97	1(1)	1(1)	1(1)	1(1)	2%	2%	
Polyubiquitin -like protein, partial	A	A	14366	122	62	2(1)	1(0)	2(1)	1(0)	19%	7%	KRY24237.1
	B	-		76	-	2(1)	-	2(1)	-	14%	-	
	-	A	12545	-	62	-	1(0)	-	1(0)	-	8%	KRY37210.1
	-	B		-	108	-	1(1)	-	1(1)	-	14%	
	C	C		80	97	1(1)	1(1)	1(1)	1(1)	14%	14%	
Polyubiquitin-B, partial	-	A	17394	-	62	-	1(0)	-	1(0)	-	5%	KRY44570.1
	B	B		76	108	2(1)	1(1)	2(1)	1(1)	11%	10%	
	C	C		80	97	1(1)	1(1)	1(1)	1(1)	10%	10%	
Polyubiquitin-C	-	A	39424	-	62	-	1(0)	-	1(0)	-	2%	KRY55776.1
	B	B		76	108	2(1)	1(1)	2(1)	1(1)	5%	4%	

Protein name	Presence in band		Mass	Scores		Matches		Sequences		Coverage		Accession number
	C	C		80	97	1(1)	1(1)	1(1)	1(1)	4%	4%	
Proactivator polypeptide, partial	B	C	56771	58	87	1(1)	2(2)	1(1)	2(2)	3%	5%	KRY46764.1
Proliferation-associated protein 2G4, partial	A	-	49607	128	-	6(4)	-	6(4)	-	14%	-	KRY53054.1
	B	B		281	164	9(9)	4(4)	9(9)	4(4)	24%	10%	
Prolyl 4-hydroxylase subunit alpha-2	C	-	130247	93	-	3(3)	-	2(2)	-	1%	-	KRY58929.1
Propionyl-CoA carboxylase alpha chain, mitochondrial	B	-	441396	219	-	4(4)	-	4(4)	-	1%	-	KRY51819.1
	-	C		-	66	-	1(1)	-	1(1)	-	0%	
Propionyl-CoA carboxylase beta chain, mitochondrial, partial	A	-	59995	74	-	1(1)	-	1(1)	-	1%	-	KRY52668.1
	C	C		100	549	2(2)	16(16)	2(2)	13(13)	4%	30%	
Protein AAR2 -like protein	A	-	68097	75	-	1(1)	-	1(1)	-	1%	-	KRY56554.1
	-	B		-	69	-	1(1)	-	1(1)	-	1%	
Protein arginine N-methyltransferase 1, partial	A	A	41784	245	62	8(8)	2(2)	7(7)	2(2)	22%	5%	KRY51927.1
	-	B		-	130	-	6(6)	-	6(6)	-	17%	
Protein arginine N-methyltransferase 6, partial	-	A	77995	-	117	-	3(3)	-	2(2)	-	3%	KRY58901.1
Protein argonaute-2	C	-	239895	69	-	1(1)	-	1(1)	-	0%	-	KRY49018.1
Protein CLEC16A, partial	C	-	246145	111	-	4(4)	-	4(4)	-	2%	-	KRY50389.1
Protein DDI1 -like protein 2	B	-	44709	118	-	1(1)	-	1(1)	-	5%	-	KRY49840.1
Protein disulfide-isomerase, partial	C	-	136243	146	-	3(3)	-	3(3)	-	2%	-	KRY60939.1
Protein ERGIC-53, partial	B	-	57042	77	-	2(2)	-	2(2)	-	3%	-	KRY52865.1
	-	C		-	69	-	1(1)	-	1(1)	-	1%	
Protein FAM98A	A	-	69473	91	-	2(2)	-	2(2)	-	3%	-	KRY55912.1
Protein HIRA	C	-	155136	229	-	6(6)	-	6(6)	-	4%	-	KRY45698.1
Protein LSM14 -like protein A	C	-	51261	137	-	2(2)	-	2(2)	-	7%	-	KRY47653.1
Protein MON2 -like protein	B	-	227085	86	-	1(1)	-	1(1)	-	1%	-	KRY53689.1
Protein pad-1, partial	-	B	269147	-	71	-	1(1)	-	1(1)	-	0%	KRY55730.1
Protein Skeletor, isoforms B/C	C	-	158475	92	-	2(2)	-	2(2)	-	1%	-	KRY58447.1
Protein transport protein Sec61 subunit alpha isoform 2	-	A	46439	-	73	-	2(1)	-	2(1)	-	4%	KRY54101.1
Protein transport protein Sec61 subunit alpha-like 2	-	A	50898	-	73	-	2(1)	-	2(1)	-	4%	KRY54103.1
putative [pyruvate dehydrogenase (acetyl-transferring)] kinase, mitochondrial, partial	A	-	53118	139	-	4(4)	-	3(3)	-	8%	-	KRY57184.1
	-	B		-	92	-	2(2)	-	2(2)	-	7%	
putative 26S protease regulatory subunit 10B	A	-	77871	114	-	3(3)	-	3(3)	-	5%	-	KRY59565.1
	B	-		462	-	14(14)	-	13(13)	-	22%	-	
putative 26S proteasome regulatory subunit rpn-6.1	B	-	51807	103	-	2(2)	-	2(2)	-	4%	-	KRY56382.1
	-	C		-	68	-	1(1)	-	1(1)	-	2%	
putative aconitate hydratase, mitochondrial	A	-	232635	134	-	2(2)	-	2(2)	-	1%	-	KRY56796.1
	B	B		97	91	3(3)	1(1)	3(3)	1(1)	2%	0%	
	-	C		-	143	-	4(3)	-	4(3)	-	2%	
putative aminopeptidase W07G4.4	C	-	66737	574	-	19(18)	-	15(14)	-	28%	-	KRY54333.1
	-	C	62585	-	100	-	3(3)	-	3(3)	-	5%	KRY54332.1
putative arginine kinase F46H5.3	A	A	45915	340	88	11(10)	1(1)	9(8)	1(1)	29%	3%	KRY58392.1
	B	-		106	-	2(2)	-	2(2)	-	6%	-	
	C	C		70	72	1(1)	1(1)	1(1)	1(1)	2%	3%	

Protein name	Presence in band		Mass	Scores		Matches		Sequences		Coverage		Accession number
putative glutamine--tRNA ligase, partial	C	-	207108	122	-	3(3)	-	3(3)	-	1%	-	KRY57355.1
putative glycerol-3-phosphate dehydrogenase 2	-	A	38473	-	126	-	1(1)	-	1(1)	-	4%	KRY56481.1
putative histone-binding protein Caf1	B	-	50720	173	-	7(7)	-	7(7)	-	15%	-	KRY53239.1
putative integrin beta-like protein	A	-	59305	83	-	2(2)	-	2(2)	-	6%	-	KRY46033.1
	B	-		56	-	1(1)	-	1(1)	-	3%	-	
putative isocitrate dehydrogenase [NAD] subunit alpha, mitochondrial, partial	-	A	43672	-	63	-	1(0)	-	1(0)	-	2%	KRY48474.1
putative methylmalonate-semialdehyde dehydrogenase [acylating], mitochondrial	-	C	47384	-	62	-	1(1)	-	1(1)	-	2%	KRY58639.1
putative oxidoreductase -like protein	A	B	46423	74	84	1(1)	1(1)	1(1)	1(1)	2%	2%	KRY46329.1
putative phenylalanine-4-hydroxylase 1	-	C	54813	-	78	-	1(1)	-	1(1)	-	2%	KRY52089.1
putative phosphoglycerate kinase	A	-	62385	605	-	20(20)	-	17(17)	-	39%	-	KRY46312.1
	-	B		-	334	-	11(11)	-	9(9)	-	21%	
	-	A	62151	-	113	-	1(1)	-	1(1)	-	2%	KRY46311.1
	C	-		109	-	2(2)	-	2(2)	-	4%	-	
putative phospholipase B-like 2, partial	-	B	65395	-	82	-	1(1)	-	1(1)	-	1%	KRY61331.1
putative pyruvate dehydrogenase E1 component subunit alpha, mitochondrial	A	-	49940	238	-	5(5)	-	5(5)	-	13%	-	KRY58181.1
	-	A	48977	-	245	-	8(8)	-	7(7)	-	17%	KRY58179.1
putative sodium/potassium-transporting ATPase subunit beta-3	A	-	35639	68	-	1(1)	-	1(1)	-	3%	-	KRY55118.1
	B	-		134	-	2(2)	-	2(2)	-	9%	-	
	C	-		91	-	1(1)	-	1(1)	-	3%	-	
putative splicing factor, arginine/serine-rich 7, partial	C	-	137028	72	-	1(1)	-	1(1)	-	1%	-	KRY51241.1
putative trans-2-enoyl-CoA reductase 1, mitochondrial	-	A	39830	-	111	-	1(1)	-	1(1)	-	3%	KRY50342.1
putative UDP-glucuronosyltransferase ugt-58	C	-	72680	67	-	1(1)	-	1(1)	-	2%	-	KRY54467.1
Pyridine nucleotide-disulfide oxidoreductase domain-containing protein 1	-	C	55815	-	224	-	6(6)	-	6(6)	-	14%	KRY46015.1
Pyruvate kinase PKLR	C	-	69929	149	-	3(3)	-	3(3)	-	6%	-	KRY57523.1
Pyruvate kinase PKM	C	-	65506	149	-	3(3)	-	3(3)	-	6%	-	KRY57524.1
Rab GDP dissociation inhibitor alpha	A	A	50324	301	68	8(8)	1(1)	7(7)	1(1)	21%	2%	KRY54650.1
	B	B		1049	125	27(26)	3(3)	19(18)	3(3)	54%	7%	
Rab proteins geranylgeranyltransferase component A	C	-	63173	88	-	2(2)	-	2(2)	-	4%	-	KRY47219.1
Ran GTPase-activating protein 1, partial	C	-	66199	190	-	5(5)	-	4(4)	-	7%	-	KRY60944.1
Replicase polyprotein 1a	-	A	82573	-	94	-	1(1)	-	1(1)	-	1%	KRY55933.1
Replication protein A 70 kDa DNA-binding subunit	-	C	72373	-	64	-	1(1)	-	1(1)	-	2%	KRY46120.1
Retrovirus-related Pol polyprotein from transposon	-	A	68132	-	78	-	1(1)	-	1(1)	-	1%	KRY48786.1
Rho GTPase-activating protein 39	B	-	229114	78	-	2(2)	-	2(2)	-	1%	-	KRY55506.1
Ribokinase, partial	-	A	34856	-	183	-	7(6)	-	7(6)	-	23%	KRY56022.1
RNA-binding motif, single-stranded-interacting protein 3	C	-	63655	98	-	3(3)	-	3(3)	-	5%	-	KRY50396.1
RNA-binding protein squid	-	A	38365	-	63	-	1(0)	-	1(0)	-	2%	KRY50692.1
	B	-		79	-	1(1)	-	1(1)	-	4%	-	
RuvB-like 2	B	-	52985	68	-	1(1)	-	1(1)	-	4%	-	KRY54932.1
RuvB-like helicase 1	B	-	111487	88	-	1(1)	-	1(1)	-	1%	-	KRY48652.1
Secernin-3, partial	-	A	110075	-	85	-	1(1)	-	1(1)	-	1%	KRY45763.1

Protein name	Presence in band		Mass	Scores		Matches		Sequences		Coverage		Accession number
secretion antigen precursor	A	-	46673	67	-	1(1)	-	1(1)	-	2%	-	CAD86782.1
	C	C		782	454	20(20)	11(11)	11(11)	6(6)	31%	20%	
Serine hydroxymethyltransferase	B	B	33657	126	107	1(1)	1(1)	1(1)	1(1)	4%	4%	KRY56265.1
	-	C		-	126	-	1(1)	-	1(1)	-	4%	
Serine protease 30	A	A	146279	229	76	4(4)	1(1)	4(4)	1(1)	4%	1%	KRY58841.1
	B	B		1025	254	18(18)	3(3)	9(9)	3(3)	8%	5%	
	C	C		604	306	9(9)	7(7)	6(6)	6(6)	6%	7%	
Serine/threonine-protein phosphatase 2B catalytic subunit 2	C	-	55902	134	-	3(3)	-	3(3)	-	7%	-	KRY57383.1
Serine/threonine-protein phosphatase 5	C	-	59857	78	-	1(1)	-	1(1)	-	2%	-	KRY53930.1
Serine--tRNA ligase, cytoplasmic	-	C	204653	-	235	-	8(8)	-	7(7)	-	4%	KRY54661.1
Signal recognition particle subunit SRP72	C	-	78986	62	-	1(1)	-	1(1)	-	1%	-	KRY48636.1
Signal transducing adapter molecule 1	B	-	46966	70	-	1(1)	-	1(1)	-	4%	-	KRY51332.1
Snake venom 5'-nucleotidase	-	A	183199	-	65	-	1(1)	-	1(1)	-	0%	KRY49427.1
	B	-		270	-	4(4)	-	3(3)	-	3%	-	
	C	C		733	154	20(20)	3(3)	14(14)	3(3)	10%	2%	
Solute carrier family 2, facilitated glucose transporter member 3	B	-	167928	168	-	2(2)	-	2(2)	-	1%	-	KRY47169.1
	-	C		-	114	-	1(1)	-	1(1)	-	0%	
Spectrin beta chain	-	C	270460	-	63	-	1(1)	-	1(1)	-	0%	KRY58047.1
Spliceosome RNA helicase DDX39B	A	-	83161	80	-	1(1)	-	1(1)	-	1%	-	KRY50703.1
	B	-		220	-	7(7)	-	7(7)	-	11%	-	
	-	C		-	195	-	5(5)	-	5(5)	-	7%	
Splicing factor 3A subunit 1, partial	B	-	116221	75	-	1(1)	-	1(1)	-	1%	-	KRY50075.1
Splicing factor 3A subunit 3	C	-	58548	68	-	2(1)	-	2(1)	-	4%	-	KRY56136.1
Stress-70 protein, mitochondrial	B	-	92371	68	-	1(1)	-	1(1)	-	1%	-	KRY58424.1
	C	-		124	-	3(3)	-	3(3)	-	3%	-	
Stress-activated protein kinase JNK	-	B	60103	-	70	-	1(1)	-	1(1)	-	1%	KRY61292.1
Stress-induced-phosphoprotein 1	A	-	62843	77	-	2(2)	-	2(2)	-	4%	-	KRY50814.1
	C	C		233	87	6(6)	2(2)	6(6)	2(2)	12%	4%	
Structural maintenance of chromosomes protein 3	B	-	180414	123		4(3)	-	4(3)	-	2%	-	KRY53012.1
Succinate dehydrogenase [ubiquinone] flavoprotein subunit, mitochondrial	C	C	97198	164	59	5(5)	1(1)	5(5)	1(1)	6%	1%	KRY54540.1
Succinate-semialdehyde dehydrogenase, mitochondrial	B	-	63166	307	-	9(9)	-	9(9)	-	19%	-	KRY54971.1
	-	C		-	92	-	3(3)	-	3(3)	-	6%	
Succinyl-CoA:3-ketoacid coenzyme A transferase 1, mitochondrial	-	C	56551	-	95	-	1(1)	-	1(1)	-	2%	KRY54562.1
Sulfhydryl oxidase 1	C		109353	230	-	7(7)	-	5(5)	-	5%	-	KRY58937.1
Sulfide:quinone oxidoreductase, mitochondrial	A	-	54403	128	-	2(2)	-	2(2)	-	5%	-	KRY46292.1
	B	B		105	109	3(3)	3(3)	3(3)	3(3)	6%	7%	
	C	-		63	-	1(1)	-	1(1)	-	2%	-	
Tau-tubulin kinase 1	-	B	114198	-	70	-	1(1)	-	1(1)	-	0%	KRY53389.1
T-complex protein 1 subunit alpha	C	C	79972	262	164	8(8)	3(3)	8(8)	3(3)	11%	4%	KRY49588.1
T-complex protein 1 subunit beta	B	-	59807	231	-	6(6)	-	6(6)	-	15%	-	KRY52365.1
	C	C		395	91	13(12)	2(2)	12(11)	2(2)	28%	4%	

Protein name	Presence in band		Mass	Scores		Matches		Sequences		Coverage		Accession number
T-complex protein 1 subunit delta, partial	B	-	25524	68	-	1(1)	-	1(1)	-	3%	-	KRY50590.1
	C	-		85	-	1(1)	-	1(1)	-	5%	-	
T-complex protein 1 subunit epsilon	C	C	127413	847	61	22(22)	1(1)	18(18)	1(1)	17%	1%	KRY50385.1
T-complex protein 1 subunit eta	A	-	175156	115	-	1(1)	-	1(1)	-	0%	-	KRY48081.1
	-	B		-	111	-	1(1)	-	1(1)	-	0%	
	-	C		-	208	-	6(6)	-	6(6)	-	4%	
	C	-	175248	185	-	2(2)	-	2(2)	-	1%	-	KRY48082.1
T-complex protein 1 subunit gamma	B	-	60486	96	-	1(1)	-	1(1)	-	2%	-	KRY47292.1
	C	C		339	158	12(12)	4(3)	11(11)	4(3)	24%	8%	
T-complex protein 1 subunit theta	B	-	151283	63	-	1(1)	-	1(1)	-	1%	-	KRY50910.1
	C	C		300	488	9(9)	13(13)	9(9)	11(11)	7%	8%	
Tetraspanin-1, partial	C	-	32574	73	-	1(1)	-	1(1)	-	4%	-	KRY57011.1
Thioredoxin reductase 1, cytoplasmic, partial	-	C	141583	-	84	-	1(1)	-	1(1)	-	0%	KRY57019.1
Titin	B	-	1732268	109	-	2(2)	-	2(2)	-	0%	-	KRY55434.1
TOM1-like protein 2	B	-	44804	67	-	1(1)	-	1(1)	-	2%	-	KRY50457.1
Transketolase	-	A	140272	-	85	-	1(1)	-	1(1)	-	0%	KRY48205.1
	B	-		82	-	1(1)	-	1(1)	-	0%	-	
	C	C		293	90	9(9)	2(2)	9(9)	2(2)	7%	1%	
Transketolase-like protein 2	-	A	140714	-	85	-	1(1)	-	1(1)	-	0%	KRY48207.1
	-	C		-	90	-	2(2)	-	2(2)	-	1%	
Translation initiation factor eIF-2B subunit beta	-	A	40208	-	78	-	1(1)	-	1(1)	-	3%	KRY60228.1
Translocation protein SEC63 -like protein	C	-	127490	61	-	1(1)	-	1(1)	-	1%	-	KRY52497.1
Transmembrane protease serine 5	A	A	41206	127	293	3(3)	9(9)	3(3)	7(7)	10%	22%	KRY50806.1
	-	B		-	155	-	3(3)	-	3(3)	-	11%	
Transmembrane protease serine 9	A	A	73208	91	117	1(1)	1(1)	1(1)	1(1)	2%	2%	KRY59262.1
	B	-		119	-	2(2)	-	2(2)	-	4%	-	
	C	-		386	-	10(10)	-	6(6)	-	10%	-	
	-	B	111650	-	254	-	3(3)	-	3(3)	-	5%	KRY58843.1
	-	C		-	306	-	7(7)	-	6(6)	-	8%	
Transposon Tf2-6 polyprotein	-	A	186969	-	78	-	1(1)	-	1(1)	-	0%	KRY45402.1
Trifunctional enzyme subunit beta, mitochondrial	A	-	47839	70	-	1(1)	-	1(1)	-	2%	-	KRY48050.1
	-	B		-	66	-	1(1)	-	1(1)	-	1%	
tRNA-splicing ligase RtcB -like protein	-	C	57770	-	98	-	1(1)	-	1(1)	-	2%	KRY54162.1
Tubulin alpha-3 chain	-	B	50322	-	93	-	2(2)	-	2(2)	-	4%	KRY61018.1
	B	-	50924	430	-	7(7)	-	7(7)	-	25%	-	KRY50534.1
	C	C		91	277	3(3)	9(9)	3(3)	8(8)	6%	21%	
Twitchin	B	-	712509	58	-	1(1)	-	1(1)	-	0%	-	KRY50893.1
Type I inositol 1,4,5-trisphosphate 5-phosphatase, partial	A	-	57850	148	-	5(5)	-	4(4)	-	9%	-	KRY50866.1
	-	B	53398	-	92	-	2(2)	-	2(2)	-	4%	KRY50867.1
Ubiquitin carboxyl-terminal hydrolase 14, partial	-	B	57580	-	77	-	1(1)	-	1(1)		2%	KRY58645.1
	C	-		192	-	5(5)	-	4(4)		9%	-	
Ubiquitin carboxyl-terminal hydrolase 15	-	C	155087	-	93	-	2(2)	-	2(2)	-	1%	KRY54182.1

Protein name	Presence in band		Mass	Scores		Matches		Sequences		Coverage		Accession number
Ubiquitin carboxyl-terminal hydrolase 4	-	C	163219	-	93	-	2(2)	-	2(2)	-	1%	KRY54180.1
Ubiquitin carboxyl-terminal hydrolase 7, partial	C	-	127949	61	-	1(1)	-	1(1)	-	1%	-	KRY49807.1
Ubiquitin-like modifier-activating enzyme 5	A	-	41307	68	-	1(1)	-	1(1)	-	4%	-	KRY47667.1
UDP-glucose 4-epimerase	A	A	86965	83	74	1(1)	2(2)	1(1)	2(2)	1%	3%	KRY59504.1
	-	B		-	68	-	1(1)	-	1(1)	-	1%	
UDP-N-acetylhexosamine pyrophosphorylase	B	-	84084	68	-	1(1)	-	1(1)	-	1%	-	KRY50795.1
	-	C		-	68	-	1(1)	-	1(1)	-	1%	
Uncharacterized protein T03_9851	B	B	42427	490	263	11(11)	6(5)	5(5)	4(3)	23%	16%	KRY58607.1
Uridine 5'-monophosphate synthase	B	-	108908	70	-	1(1)	-	1(1)	-	0%	-	KRY46591.1
UTP--glucose-1-phosphate uridylyltransferase, partial	-	B	154987	-	75	-	1(1)	-	1(1)	-	0%	KRY53918.1
UV excision repair protein RAD23 -like protein B, partial	-	C	53628	-	131	-	2(2)	-	2(2)	-	6%	KRY52283.1
Vacuolar protein sorting-associated protein 26B	B	-	215378	86	-	1(1)	-	1(1)	-	1%	-	KRY53691.1
Vacuolar protein sorting-associated protein 45	C	-	161398	123	-	3(3)	-	3(3)	-	2%	-	KRY55296.1
Vacuolar protein sorting-associated protein 4A, partial	-	C	56812	-	58	-	1(1)	-	1(1)	-	2%	KRY53888.1
Very long-chain specific acyl-CoA dehydrogenase, mitochondrial, partial	C	-	70153	245	-	10(10)	-	9(9)	-	14%	-	KRY52870.1
V-type proton ATPase subunit C, partial	-	A	42184	-	87	-	1(1)	-	1(1)	-	3%	KRY54431.1
Xaa-Pro aminopeptidase 1, partial	C	-	74254	115	-	2(2)	-	2(2)	-	3%	-	KRY57414.1
