# Supplementary material for: Immunoproteomic Analysis of Trichinella britovi Proteins Recognized by IgG Antibodies from Meat Juice of Carnivores Naturally Infected with T. britovi
Source: Pathogens. 2022 Oct 6;11(10):1155. doi: 10.3390/pathogens11101155 (PMC9612144; doi:10.3390/pathogens11101155)
Supplement: Supplementary file 1 [file pathogens-11-01155-s001.zip › Table S2.pdf]

Table S2. Results of the LC-MS/MS analysis of selected gel fragments for three examined raccoon dogs: R1, R2 and R3.

Protein name	Presence in band no.			Mass	Scores			Matches			Sequences			Coverage			Accession number
	R1	R2	R3		R1	R2	R3	R1	R2	R3	R1	R2	R3	R1	R2	R3	
1,4-alpha-glucan-branching enzyme	C	-	-	155692	71	-	-	1(1)	-	-	1(1)	-	-	1%	-	-	KRY55089.1
	D	-	-		62	-	-	1(1)	-	-	1(1)	-	-	1%	-	-	
	-	C	-	160167	-	64	-	-	1(1)	-	-	1(1)	-	-	0%	-	KRY55088.1
1,5-anhydro-D-fructose reductase	A	-	-	37989	169	-	-	2(2)	-	-	2(2)	-	-	10%	-	-	KRY43899.1
	B	B	B		66	107	144	1(1)	2(1)	2(2)	1(1)	2(1)	2(2)	2%	11%	9%	
	C	C	C		131	80	121	1(1)	1(1)	2(2)	1(1)	1(1)	2(2)	6%	2%	11%	
	D	-	-		204	-	-	2(2)	-	-	2(2)	-	-	10%	-	-	
	-	-	E		-	-	63	-	-	1(1)	-	-	1(1)	-	-	2%	
	-	-	F		-	-	65	-	-	1(1)	-	-	1(1)	-	-	2%	
1-phosphatidylinositol 4,5-bisphosphate phosphodiesterase beta-4, partial	D	-	-	145873	75	-	-	1(1)	-	-	1(1)	-	-	0%	-	-	KRY59603.1
	-	D	-	140352	-	114	-	-	2(2)	-	-	2(2)	-	-	1%	-	KRY59604.1
2,3-bisphosphoglycerate-independent phosphoglycerate mutase	A	A	A	59212	187	131	154	4(3)	4(4)	5(5)	4(3)	4(4)	5(5)	10%	9%	10%	KRY48325.1
	B	B	B		86	138	200	2(2)	4(4)	6(6)	2(2)	4(4)	6(6)	3%	8%	15%	
	C	C	-		336	257	-	10(10)	8(8)	-	10(10)	7(7)	-	21%	13%	-	
	D	D	D		247	198	72	7(7)	5(5)	1(1)	7(7)	5(5)	1(1)	16%	12%	2%	
	-	-	E		-	-	220	-	-	6(6)	-	-	6(6)	-	-	12%	
	-	-	F		-	-	127	-	-	2(2)	-	-	2(2)	-	-	4%	
	-	-	G		-	-	92	-	-	2(2)	-	-	2(2)	-	-	4%	
26S protease regulatory subunit 10B	A	A	A	55162	150	184	160	2(2)	5(5)	3(3)	2(2)	5(5)	3(3)	5%	12%	8%	KRY59566.1
	B	B	B		210	247	364	6(6)	9(8)	11(11)	6(6)	9(8)	10(10)	14%	21%	24%	
	D	-	-		96	-	-	1(1)	-	-	1(1)	-	-	3%	-	-	
26S protease regulatory subunit 4, partial	A	-	-	77301	73	-	-	1(1)	-	-	1(1)	-	-	1%	-	-	KRY52921.1
	B	B	B		90	94	93	1(1)	2(2)	2(2)	1(1)	2(2)	2(2)	1%	3%	3%	
26S protease regulatory subunit 6B	B	B	-	40507	78	105	-	1(1)	2(2)	-	1(1)	2(2)	-	2%	6%	-	KRY48180.1
26S proteasome non-ATPase regulatory subunit 1, partial	-	D	-	125778	-	80	-	-	1(1)	-	-	1(1)	-	-	0%	-	KRY58375.1
	-	-	E		-	-	246	-	-	6(6)	-	-	5(5)	-	-	6%	
	-	-	D	119157	-	-	86	-	-	1(1)	-	-	1(1)	-	-	1%	KRY58377.1
	-	-	F		-	-	67	-	-	1(1)	-	-	1(1)	-	-	1%	
26S proteasome non-ATPase regulatory subunit 12	C	C	-	67345	61	82	-	1(1)	2(2)	-	1(1)	2(2)	-	1%	3%	-	KRY57716.1
26S proteasome non-ATPase regulatory subunit 3, partial	C	-	C	59673	56	-	127	1(0)	-	1(1)	1(0)	-	1(1)	2%	-	4%	KRY53037.1
	-	D	-		-	77	-	-	1(1)	-	-	1(1)	-	-	2%	-	
	-	-	G		-	-	78	-	-	1(1)	-	-	1(1)	-	-	2%	
26S proteasome non-ATPase regulatory subunit 4	-	-	B	42698	-	-	163	-	-	4(4)	-	-	3(3)	-	-	6%	KRY60873.1
	C	-	-		79	-	-	1(1)	-	-	1(1)	-	-	4%	-	-	
26S proteasome non-ATPase regulatory subunit 6, partial	A	A	A	50950	90	119	62	1(1)	3(3)	1(1)	1(1)	3(3)	1(1)	2%	6%	2%	KRY50659.1
2-oxoglutarate dehydrogenase, mitochondrial, partial	-	-	C	122337	-	-	61	-	-	1(1)	-	-	1(1)	-	-	1%	KRY52012.1
	-	D	D		-	73	67	-	1(1)	1(1)	-	1(1)	1(1)	-	1%	1%	
32 kDa beta-galactoside-binding lectin	-	A	A	31910	-	99	81	-	1(1)	1(1)	-	1(1)	1(1)	-	4%	4%	KRY59236.1
	B	B	B		123	91	99	2(2)	3(3)	1(1)	2(2)	3(3)	1(1)	9%	12%	4%	
	C	C	C		194	183	138	4(4)	3(3)	3(3)	4(4)	3(3)	3(3)	18%	14%	12%	
	D	D	D		182	144	105	4(4)	3(3)	3(3)	4(4)	3(3)	3(3)	17%	12%	12%	
	-	-	E		-	-	114	-	-	1(1)	-	-	1(1)	-	-	5%	
	-	-	F		-	-	105	-	-	2(2)	-	-	2(2)	-	-	8%	
	-	-	G		-	-	107	-	-	1(1)	-	-	1(1)	-	-	5%	

32 kDa beta-galactoside-binding lectin, partial	A	-	A	37367	113	-	61	2(2)	-	1(1)	2(2)	-	1(1)	9%	-	3%	KRY52250.1
	B	B	B		85	89	103	1(1)	1(1)	4(4)	1(1)	1(1)	4(4)	3%	3%	11%	
	C	-	C		104	-	118	3(3)	-	4(4)	3(3)	-	4(4)	10%	-	12%	
	D	D	D		93	103	93	1(1)	3(3)	1(1)	1(1)	3(3)	1(1)	3%	3%	3%	
	-	-	E		-	-	89	-	-	1(1)	-	-	1(1)	-	-	3%	
	-	-	F		-	-	97	-	-	2(2)	-	-	2(2)	-	-	6%	
	-	-	G		-	-	95	-	-	2(2)	-	-	2(2)	-	-	6%	
3-ketoacyl-CoA thiolase, mitochondrial	A	A	A	42697	2573	950	1308	51(51)	31(30)	34(34)	22(22)	16(16)	18(18)	72%	56%	64%	KRY51064.1
	B	B	B		165	139	322	3(2)	5(5)	8(8)	3(2)	5(5)	7(7)	8%	15%	28%	
	C	C	C		464	230	369	12(12)	5(5)	6(6)	9(9)	5(5)	4(4)	40%	13%	17%	
	D	D	D		536	204	137	13(13)	4(4)	3(3)	11(11)	4(4)	3(3)	41%	13%	8%	
	-	-	E		-	-	227	-	-	7(7)	-	-	6(6)	-	-	16%	
	-	-	F		-	-	143	-	-	3(3)	-	-	3(3)	-	-	10%	
	-	-	G		-	-	154	-	-	3(3)	-	-	3(3)	-	-	8%	
40S ribosomal protein S13	-	C	C	25073	-	60	61	-	1(1)	1(1)	-	1(1)	1(1)	-	3%	3%	KRY56989.1
40S ribosomal protein S18	-	-	A	26097	-	-	84	-	-	2(2)	-	-	2(2)	-	-	9%	KRY55489.1
	-	B	-		-	89	-	-	1(1)	-	-	1(1)	-	-	6%	-	
	C	C	C		75	97	77	1(1)	1(1)	1(1)	1(1)	1(1)	1(1)	4%	6%	4%	
	-	D	D		-	62	71	-	1(1)	1(1)	-	1(1)	1(1)	-	4%	4%	
	-	-	E		-	-	79	-	-	1(1)	-	-	1(1)	-	-	6%	
	-	-	G		-	-	110	-	-	1(1)	-	-	1(1)	-	-	6%	
40S ribosomal protein S3	C	C	-	38314	77	74	-	1(1)	2(2)	-	1(1)	2(2)	-	5%	6%	-	KRY60420.1
	D	D	D		91	59	108	3(3)	1(1)	1(1)	3(3)	1(1)	1(1)	8%	2%	3%	
	-	-	G		-	-	67	-	-	1(1)	-	-	1(1)	-	-	2%	
40S ribosomal protein S3a, partial	-	-	C	32687	-	-	59	-	-	1(1)	-	-	1(1)	-	-	2%	KRY56687.1
	D	-	-		130	-	-	1(1)	-	-	1(1)	-	-	6%	-	-	
40S ribosomal protein S4	D	-	-	31072	58	-	-	1(1)	-	-	1(1)	-	-	3%	-	-	KRY48988.1
40S ribosomal protein S5, partial	C	C	-	24818	104	66	-	1(1)	1(1)	-	1(1)	1(1)	-	6%	6%	-	KRY49420.1
	D	-	-		110	-	-	1(1)	-	-	1(1)	-	-	6%	-	-	
40S ribosomal protein S8	C	C	C	72413	82	98	78	1(1)	1(1)	2(2)	1(1)	1(1)	2(2)	2%	2%	2%	KRY60196.1
40S ribosomal protein S9	C	-	-	26490	75	-	-	1(1)	-	-	1(1)	-	-	4%	-	-	KRY47012.1
	D	-	-		76	-	-	1(1)	-	-	1(1)	-	-	4%	-	-	
	-	-	G		-	-	75	-	-	1(1)	-	-	1(1)	-	-	4%	
4-hydroxybutyrate coenzyme A transferase, partial	A	A	A	53804	161	116	169	6(6)	3(3)	6(6)	5(5)	3(3)	5(5)	10%	5%	10%	KRY56869.1
	B	B	B		271	265	352	10(10)	9(9)	14(14)	10(10)	8(8)	11(11)	19%	18%	22%	
	-	C	C		-	90	61	-	1(1)	1(1)	-	1(1)	1(1)	-	2%	2%	
	-	-	E		-	-	72	-	-	1(1)	-	-	1(1)	-	-	1%	
4-hydroxyphenylpyruvate dioxygenase	-	A	-	44422	-	82	-	-	2(2)	-	-	2(2)	-	-	6%	-	KRY55837.1
5'-3' exoribonuclease 1	-	C	C	237195	-	80	106	-	1(1)	1(1)	-	1(1)	1(1)	-	0%	0%	KRY61082.1
5'-AMP-activated protein kinase catalytic subunit alpha-2	C	C	-	94860	165	94	-	2(2)	1(1)	-	1(1)	1(1)	-	2%	2%	-	KRY53623.1
60 kDa heat shock protein, mitochondrial	A	A	A	158357	385	224	332	10(10)	8(7)	9(9)	9(9)	8(7)	9(9)	8%	7%	8%	KRY51676.1
	B	B	B		155	198	243	5(5)	7(7)	8(8)	5(5)	7(7)	8(8)	4%	6%	7%	
	C	C	C		1293	1316	482	35(35)	31(31)	15(15)	26(26)	24(24)	15(15)	20%	21%	12%	
	D	D	-		83	89	-	2(2)	1(1)	-	2(2)	1(1)	-	2%	1%	-	
	-	-	E		-	-	112	-	-	3(3)	-	-	3(3)	-	-	2%	
	-	-	F		-	-	79	-	-	1(1)	-	-	1(1)	-	-	0%	
60 kDa SS-A/Ro ribonucleoprotein, partial	C	C	C	69458	631	577	698	14(13)	14(14)	17(17)	10(9)	11(11)	11(11)	24%	26%	25%	KRY47029.1
	D	-	-		64	-	-	1(1)	-	-	1(1)	-	-	1%	-	-	
60S acidic ribosomal protein P0	A	A	A	32092	145	62	144	2(2)	1(0)	3(3)	2(2)	1(0)	3(3)	8%	4%	13%	KRY54528.1

	-	B	B		-	94	111	-	1(1)	1(1)	-	1(1)	1(1)	-	3%	3%	
	C	C	-		103	138	-	2(2)	2(2)	-	2(2)	2(2)	-	8%	9%	-	
	D	D	-		69	93	-	2(2)	1(1)	-	2(2)	1(1)	-	7%	5%	-	
	-	-	E		-	-	119	-	-	1(1)	-	-	1(1)	-	-	5%	
	-	-	F		-	-	67	-	-	1(1)	-	-	1(1)	-	-	4%	
60S ribosomal protein L15	C	C	-	84126	68	104	-	1(1)	1(1)	-	1(1)	1(1)	-	1%	1%	-	KRY49952.1
	-	-	F		-	-	89	-	-	1(1)	-	-	1(1)	-	-	1%	
	-	-	G		-	-	112	-	-	2(2)	-	-	2(2)	-	-	3%	
60S ribosomal protein L17, partial	-	D	-	146568	-	66	-	-	1(1)	-	-	1(1)	-	-	0%	-	KRY50982.1
60S ribosomal protein L18a	-	-	C	22864	-	-	66	-	-	1(1)	-	-	1(1)	-	-	5%	KRY60168.1
	-	-	G		-	-	63	-	-	1(1)	-	-	1(1)	-	-	4%	
60S ribosomal protein L3	A	A	A	34644	111	83	88	5(5)	5(4)	3(3)	5(5)	5(4)	3(3)	15%	14%	8%	KRY46416.1
	B	-	-		64	-	-	2(2)	-	-	2(2)	-	-	5%	-	-	
	D	-	-		68	-	-	1(1)	-	-	1(1)	-	-	3%	-	-	
	-	-	F		-	-	68	-	-	1(1)	-	-	1(1)	-	-	3%	
	-	-	G		-	-	67	-	-	1(1)	-	-	1(1)	-	-	3%	
60S ribosomal protein L4	-	A	-	31108	-	111	-	-	2(2)	-	-	2(2)	-	-	8%	-	KRY61057.1
	C	-	C		70	-	60	1(1)	-	1(1)	1(1)	-	1(1)	3%	-	3%	
	-	D	-		-	64	-	-	1(1)	-	-	1(1)	-	-	1%	-	
	-	-	E		-	-	65	-	-	1(1)	-	-	1(1)	-	-	3%	
	-	-	G		-	-	67	-	-	2(1)	-	-	2(1)	-	-	5%	
60S ribosomal protein L4, partial	-	-	A	34612	-	-	99	-	-	4(4)	-	-	4(4)	-	-	13%	KRY61059.1
60S ribosomal protein L7a	-	-	B	48759	-	-	78	-	-	1(1)	-	-	1(1)	-	-	3%	KRY60094.1
	C	-	C		99	-	83	1(1)	-	1(1)	1(1)	-	1(1)	3%	-	3%	
	D	-	-		79	-	-	1(1)	-	-	1(1)	-	-	3%	-	-	
6-phosphofructokinase	-	C	C	94935	-	96	109	-	1(1)	1(1)	-	1(1)	1(1)	-	2%	2%	KRY50273.1
	-	D	-		-	71	-	-	1(1)	-	-	1(1)	-	-	1%	-	
	-	-	G		-	-	69	-	-	1(1)	-	-	1(1)	-	-	1%	
Acetolactate synthase-like protein	C	-	-	69865	63	-	-	1(1)	-	-	1(1)	-	-	1%	-	-	KRY51410.1
Acetyl-CoA acetyltransferase, mitochondrial	-	A	A	43775	-	89	68	-	2(2)	1(1)	-	2(2)	1(1)	-	5%	2%	KRY60246.1
Actin-interacting protein 1	A	-	-	71319	96	-	-	2(2)	-	-	2(2)	-	-	3%	-	-	KRY51005.1
	C	-	C		1107	-	737	29(28)	-	21(21)	26(25)	-	19(19)	49%	-	37%	
Actin-like protein 6A	B	-	-	120874	94	-	-	1(1)	-	-	1(1)	-	-	0%	-	-	KRY56723.1
Actin-related protein 2, partial	-	A	-	155475	-	88	-	-	1(1)	-	-	1(1)	-	-	0%	-	KRY53921.1
	B	-	B		69	-	75	1(1)	-	1(1)	1(1)	-	1(1)	0%	-	1%	
	-	-	E		-	-	83	-	-	1(1)	-	-	1(1)	-	-	0%	
	-	-	G		-	-	64	-	-	1(1)	-	-	1(1)	-	-	0%	
Actin-related protein 2/3 complex subunit 2	A	A	A	132079	230	255	249	5(5)	8(7)	5(5)	5(5)	8(7)	5(5)	5%	6%	5%	KRY56438.1
	-	B	B		-	91	82	-	1(1)	1(1)	-	1(1)	1(1)	-	0%	1%	
	C	C	C		141	66	124	2(2)	1(1)	2(2)	2(2)	1(1)	2(2)	2%	1%	1%	
	D	D	-		153	120	-	3(3)	2(2)	-	3(3)	2(2)	-	3%	1%	-	
Actin-related protein 3	A	-	-	128876	61	-	-	1(1)	-	-	1(1)	-	-	1%	-	-	KRY47903.1
	-	-	B		-	-	62	-	-	1(1)	-	-	1(1)	-	-	1%	
Adducin-related protein 1	-	A	A	80242	-	94	68	-	1(1)	1(1)	-	1(1)	1(1)	-	1%	1%	KRY47021.1
	B	B	B		94	101	199	2(2)	2(2)	5(5)	2(2)	2(2)	5(5)	3%	3%	9%	
	C	C	C		96	144	111	3(3)	5(5)	3(3)	3(3)	5(5)	3(3)	4%	7%	5%	
	D	-	-		76	-	-	1(1)	-	-	1(1)	-	-	1%	-	-	
	-	-	E		-	-	97	-	-	3(3)	-	-	3(3)	-	-	4%	
	-	-	G		-	-	90	-	-	1(1)	-	-	1(1)	-	-	1%	

Adenylosuccinate lyase	A	A	A	185820	141	65	80	5(5)	1(1)	2(2)	5(5)	1(1)	2(2)	2%	0%	1%	KRY47589.1
	B	B	B		751	804	1219	21(19)	22(22)	34(34)	14(13)	14(14)	20(20)	10%	10%	11%	
Adenylosuccinate synthetase, partial	A	A	A	50894	307	97	225	9(9)	2(2)	5(5)	9(9)	2(2)	5(5)	22%	4%	12%	KRY50054.1
	B	B	B		218	234	200	7(7)	7(7)	5(5)	6(6)	7(7)	4(4)	15%	17%	9%	
	C	-	C		73	-	75	1(1)	-	1(1)	1(1)	-	1(1)	2%	-	1%	
	D	D	D		90	112	77	2(2)	2(2)	1(1)	2(2)	2(2)	1(1)	5%	5%	2%	
	-	-	E		-	-	137	-	-	3(3)	-	-	3(3)	-	-	6%	
	-	-	F		-	-	65	-	-	1(1)	-	-	1(1)	-	-	2%	
	-	-	G		-	-	63	-	-	1(0)	-	-	1(0)	-	-	2%	
Adenylyl cyclase-associated protein 2	B	B	B	88901	113	126	142	2(2)	3(3)	3(3)	2(2)	3(3)	3(3)	3%	5%	5%	KRY58311.1
	-	-	E		-	-	74	-	-	1(1)	-	-	1(1)	-	-	1%	
ADP-ribose pyrophosphatase, mitochondrial	A	A	-	55835	150	145	-	4(3)	4(3)	-	4(3)	4(3)	-	9%	9%	-	KRY50360.1
	C	C	C	58846	108	83	71	2(2)	1(1)	1(1)	2(2)	1(1)	1(1)	4%	2%	2%	KRY48334.1
	-	B	B	55423	-	123	165	-	3(3)	4(4)	-	3(3)	3(3)	-	7%	7%	KRY57225.1
	-	D	-		-	90	-	-	2(2)	-	-	2(2)	-	-	4%	-	
ADP-ribose pyrophosphatase, mitochondrial, partial	B	B	B	137379	144	125	185	4(4)	3(3)	4(4)	4(4)	2(2)	3(3)	1%	2%	3%	KRY52752.1
	C	C	C		399	252	297	14(14)	12(11)	7(7)	10(10)	10(9)	5(5)	9%	7%	4%	
	-	D	-		-	115	-	-	4(3)	-	-	4(3)	-	-	3%	-	
	-	-	E		-	-	93	-	-	1(1)	-	-	1(1)	-	-	0%	
Alanine aminotransferase 2	-	A	-	63694	-	98	-	-	1(1)	-	-	1(1)	-	-	2%	-	KRY55706.1
	B	B	B		102	139	118	1(1)	3(3)	2(1)	1(1)	3(3)	2(1)	2%	4%	3%	
	-	C	C		-	79	62	-	1(1)	1(1)	-	1(1)	1(1)	-	2%	1%	
	-	-	E		-	-	105	-	-	2(2)	-	-	2(2)	-	-	3%	
	-	-	F		-	-	105	-	-	2(2)	-	-	2(2)	-	-	3%	
	-	-	G		-	-	100	-	-	1(1)	-	-	1(1)	-	-	2%	
Alanine--tRNA ligase, cytoplasmic	D	-	-	114270	139	-	-	3(3)	-	-	3(3)	-	-	4%	-	-	KRY47973.1
	-	-	G		-	-	64	-	-	1(1)	-	-	1(1)	-	-	1%	
Aldehyde dehydrogenase, mitochondrial	-	-	B	94777	-	-	62	-	-	1(1)	-	-	1(1)	-	-	1%	KRY52913.1
	-	C	-		-	65	-	-	1(1)	-	-	1(1)	-	-	1%	-	
Alpha,alpha-trehalose-phosphate synthase[UDP-forming] 2	-	-	E	157392	-	-	67	-	-	1(1)	-	-	1(1)	-	-	1%	KRY47139.1
Alpha-actinin, sarcomeric	D	D	D	105768	970	921	505	40(40)	40(40)	28(27)	25(25)	30(30)	20(19)	30%	35%	25%	KRY53594.1
Alpha-L-fucosidase	A	A	A	55241	142	129	131	3(3)	5(5)	5(5)	3(3)	5(5)	5(5)	6%	10%	10%	KRY54685.1
	-	B	-		-	70	-	-	2(2)	-	-	2(2)	-	-	4%	-	
Alpha-mannosidase 2C1, partial	-	D	-	117975	-	278	-	-	8(8)	-	-	8(8)	-	-	8%	-	KRY61141.1
	-	-	E		-	-	94	-	-	2(2)	-	-	2(2)	-	-	2%	
Angiotensin-converting enzyme	D	D	D	105899	252	200	92	8(8)	4(4)	2(2)	8(8)	4(4)	2(2)	11%	6%	2%	KRY61047.1
	-	-	E		-	-	70	-	-	1(1)	-	-	1(1)	-	-	1%	
Ankyrin-2, partial	-	B	B	821521	-	82	66	-	1(1)	1(1)	-	1(1)	1(1)	-	0%	0%	KRY56996.1
	C	C	C		200	229	150	3(3)	7(7)	3(3)	3(3)	7(7)	3(3)	0%	1%	0%	
	D	-	D		320	-	88	10(10)	-	1(1)	10(10)	-	1(1)	1%	-	0%	
	-	-	E		-	-	144	-	-	5(5)	-	-	5(5)	-	-	0%	
	-	-	F		-	-	142	-	-	3(3)	-	-	3(3)	-	-	0%	
	-	-	G		-	-	83	-	-	2(2)	-	-	2(2)	-	-	0%	
Antigen -like protein	D	D	D	157979	319	199	108	9(9)	8(8)	2(2)	9(9)	8(8)	2(2)	8%	6%	1%	KRY50726.1
	-	-	E		-	-	90	-	-	2(2)	-	-	2(2)	-	-	1%	
AP-2 complex subunit alpha-2	-	-	C	106493	-	-	71	-	-	1(1)	-	-	1(1)	-	-	1%	KRY60602.1
	D	D	-		62	83	-	1(1)	1(1)	-	1(1)	1(1)	-	1%	1%	-	
	-	-	G		-	-	82	-	-	1(1)	-	-	1(1)	-	-	1%	
AP-2 complex subunit beta	-	-	C	106080	-	-	85	-	-	1(1)	-	-	1(1)	-	-	1%	KRY52390.1

	D	D	D		143	320	147	4(4)	8(8)	2(2)	4(4)	7(7)	2(2)	5%	10%	2%	
	-	-	G		-	-	77	-	-	1(1)	-	-	1(1)	-	-	1%	
Apoptosis-inducing factor 1, mitochondrial	-	C	-	69452	-	66	-	-	1(1)	-	-	1(1)	-	-	1%	-	KRY56249.1
Arrestin domain-containing protein 3, partial	-	B	B	64593	-	84	88	-	1(1)	1(1)	-	1(1)	1(1)	-	2%	2%	KRY54875.1
Asparagine--tRNA ligase, cytoplasmic	C	C	C	101632	535	420	237	13(13)	13(13)	8(8)	12(12)	11(11)	7(7)	16%	14%	8%	KRY51836.1
Aspartate aminotransferase, mitochondrial, partial	-	A	A	47470	-	132	143	-	2(2)	3(3)	-	2(2)	3(3)	-	7%	9%	KRY49869.1
	D	-	-		82	-	-	1(1)	-	-	1(1)	-	-	3%	-	-	
Aspartate--tRNA ligase, cytoplasmic	B	B	B	57949	87	90	154	1(1)	2(2)	4(4)	1(1)	2(2)	4(4)	1%	4%	8%	KRY49144.1
Aspartate--tRNA ligase, mitochondrial	C	C	C	70007	96	59	70	1(1)	1(0)	1(1)	1(1)	1(0)	1(1)	1%	1%	1%	KRY48162.1
Aspartyl aminopeptidase	B	B	B	52372	152	272	441	4(4)	6(6)	10(10)	4(4)	6(6)	8(8)	10%	16%	26%	KRY46829.1
Atlastin-2	C	C	C	68364	183	103	182	5(5)	1(1)	3(3)	5(5)	1(1)	3(3)	11%	2%	6%	KRY58631.1
ATP synthase subunit alpha, mitochondrial, partial	A	A	A	64231	86	93	128	1(1)	1(1)	2(2)	1(1)	1(1)	2(2)	2%	1%	4%	KRY49394.1
	B	B	B		82	133	213	3(3)	1(1)	5(5)	3(3)	1(1)	5(5)	5%	2%	11%	
	C	C	C		204	184	130	5(5)	4(4)	3(3)	5(5)	4(4)	3(3)	11%	8%	6%	
	D	D	-		161	96	-	4(4)	1(1)	-	4(4)	1(1)	-	9%	3%	-	
	-	-	E		-	-	117	-	-	2(2)	-	-	2(2)	-	-	4%	
	-	-	F		-	-	102	-	-	2(2)	-	-	2(2)	-	-	4%	
	-	-	G		-	-	107	-	-	3(3)	-	-	3(3)	-	-	7%	
ATPase family AAA domain-containing protein 3	-	C	C	71288	-	65	120	-	1(1)	2(2)	-	1(1)	2(2)	-	1%	3%	KRY52366.1
ATP-binding cassette sub-family E member 1, partial	C	C	C	113463	94	149	234	2(2)	2(2)	9(9)	2(2)	2(2)	9(9)	3%	3%	11%	KRY56471.1
ATP-dependent RNA helicase DDX19A, partial	B	-	-	114761	76	-	-	1(1)	-	-	1(1)	-	-	1%	-	-	KRY47762.1
ATP-dependent RNA helicase DDX3X	-	C	C	83346	-	62	105	-	1(1)	1(1)	-	1(1)	1(1)	-	1%	1%	KRY46364.1
ATP-dependent RNA helicase DDX3Y	C	C	C	84608	77	75	99	1(1)	1(1)	2(2)	1(1)	1(1)	2(2)	1%	1%	3%	KRY57048.1
	-	-	E		-	-	70	-	-	1(1)	-	-	1(1)	-	-	1%	
	-	-	G		-	-	69	-	-	1(1)	-	-	1(1)	-	-	1%	
Band 4.1-like protein 1, partial	-	C	-	79147	-	65	-	-	1(1)	-	-	1(1)	-	-	1%	-	KRY54007.1
Beta-hexosaminidase subunit alpha	-	-	C	63810	-	-	66	-	-	1(1)	-	-	1(1)	-	-	1%	KRY61443.1
	D	-	D		70	-	68	1(1)	-	1(1)	1(1)	-	1(1)	1%	-	1%	
BRCA1-A complex subunit BRE, partial	-	-	B	52274	-	-	83	-	-	1(1)	-	-	1(1)	-	-	2%	KRY48007.1
Breast cancer anti-estrogen resistance protein 1, partial	-	B	-	131710	-	76	-	-	1(1)	-	-	1(1)	-	-	0%	-	KRY51653.1
	-	C	C		-	93	111	-	1(1)	1(1)	-	1(1)	1(1)	-	0%	0%	
	-	-	E		-	-	75	-	-	1(1)	-	-	1(1)	-	-	0%	
C-1-tetrahydrofolate synthase, cytoplasmic	-	A	A	125620	-	72	82	-	1(1)	1(1)	-	1(1)	1(1)	-	0%	0%	KRY60034.1
	-	B	B		-	76	82	-	1(1)	1(1)	-	1(1)	1(1)	-	0%	0%	
	C	-	C		82	-	95	1(1)	-	2(2)	1(1)	-	2(2)	0%	-	1%	
	D	D	D		403	395	155	14(14)	15(15)	7(7)	14(14)	14(14)	7(7)	14%	13%	6%	
	-	-	E		-	-	95	-	-	2(2)	-	-	2(2)	-	-	1%	
	-	-	G		-	-	77	-	-	1(1)	-	-	1(1)	-	-	0%	
Calcium-transporting ATPase sarcoplasmic/endoplasmic reticulum type	D	D	D	123299	201	292	122	6(6)	10(10)	4(3)	6(6)	8(8)	4(3)	7%	9%	4%	KRY56386.1
	-	-	E		-	-	97	-	-	2(2)	-	-	2(2)	-	-	2%	
	-	-	F		-	-	88	-	-	2(2)	-	-	2(2)	-	-	1%	
	-	-	G		-	-	105	-	-	2(2)	-	-	2(2)	-	-	2%	
Calnexin, partial	C	C	C	66552	131	86	92	3(3)	2(1)	2(2)	3(3)	2(1)	2(2)	6%	4%	3%	KRY56739.1
Calpain clp-1	B	B	B	150994	80	87	80	1(1)	1(1)	1(1)	1(1)	1(1)	1(1)	0%	0%	0%	KRY60775.1
	C	C	C		620	303	307	17(17)	15(14)	10(8)	11(11)	11(11)	7(6)	10%	10%	6%	
	D	D	D		117	76	80	2(2)	1(1)	2(1)	2(2)	1(1)	2(1)	2%	0%	1%	
	-	-	E		-	-	111	-	-	4(4)	-	-	4(4)	-	-	3%	
	-	-	F		-	-	87	-	-	1(1)	-	-	1(1)	-	-	0%	
	-	-	G		-	-	77	-	-	2(2)	-	-	2(2)	-	-	1%	

Calponin -like protein OV9M	B	B	B	90680	111	145	102	1(1)	4(4)	4(4)	1(1)	4(4)	4(4)	1%	5%	7%	KRY53098.1
Calponin -like protein OV9M, partial	-	-	A	54494	-	-	85	-	-	2(2)	-	-	2(2)	-	-	5%	KRY56611.1
	B	-	B		73	-	236	1(1)	-	5(5)	1(1)	-	5(5)	2%	-	16%	
	-	-	C		-	-	84	-	-	1(1)	-	-	1(1)	-	-	3%	
Calreticulin, partial	A	A	A	51118	294	256	212	4(4)	6(6)	4(4)	4(4)	6(6)	4(4)	15%	16%	13%	KRY53845.1
	B	B	B		375	510	486	8(8)	13(13)	9(9)	7(7)	11(11)	8(8)	21%	37%	33%	
	C	-	-		164	-	-	3(3)	-	-	3(3)	-	-	12%	-	-	
Calsequestrin-1	A	A	-	60111	67	66	-	3(2)	1(1)	-	3(2)	1(1)	-	6%	1%	-	KRY61439.1
	B	B	B		193	307	424	6(6)	10(10)	15(15)	5(5)	8(8)	10(10)	10%	16%	22%	
Calsyntenin-1, partial	D	D	-	111629	173	107	-	2(2)	1(1)	-	2(2)	1(1)	-	3%	1%	-	KRY49690.1
cAMP-dependent protein kinase catalytic subunit alpha, partial	A	-	-	52893	68	-	-	1(1)	-	-	1(1)	-	-	1%	-	-	KRY51804.1
cAMP-dependent protein kinase regulatory subunit	A	A	A	47292	90	169	85	1(1)	2(2)	1(1)	1(1)	2(2)	1(1)	3%	7%	3%	KRY58719.1
	B	-	B		104	-	109	1(1)	-	1(1)	1(1)	-	1(1)	3%	-	3%	
	-	-	C		-	-	63	-	-	1(1)	-	-	1(1)	-	-	3%	
Carboxylesterase 4A	C	-	-	97483	69	-	-	1(1)	-	-	1(1)	-	-	1%	-	-	KRY49050.1
CCA tRNA nucleotidyltransferase 1, mitochondrial	B	-	-	86959	61	-	-	1(1)	-	-	1(1)	-	-	1%	-	-	KRY45865.1
Chymotrypsin-like elastase family member 2A, partial	A	A	A	46401	564	337	446	19(17)	9(9)	12(12)	15(14)	7(7)	11(11)	46%	23%	41%	KRY59723.1
Chymotrypsinogen B	C	C	C	141902	225	136	74	5(5)	2(2)	2(2)	5(5)	2(2)	2(2)	5%	2%	1%	KRY53598.1
Clathrin heavy chain 1	-	-	C	196138	-	-	67	-	-	1(1)	-	-	1(1)	-	-	0%	KRY52726.1
	D	D	D		95	97	96	1(1)	2(2)	2(2)	1(1)	2(2)	2(2)	0%	1%	1%	
	-	-	E		-	-	205	-	-	5(5)	-	-	5(5)	-	-	3%	
	-	-	F		-	-	221	-	-	6(6)	-	-	6(6)	-	-	4%	
	-	-	G		-	-	184	-	-	4(4)	-	-	4(4)	-	-	2%	
Cleavage and polyadenylation specificity factor subunit 2	B	B	-	142890	97	95	-	2(2)	2(2)	-	2(2)	2(2)	-	1%	1%	-	KRY53654.1
	C	C	C		333	165	181	11(11)	5(5)	4(4)	10(10)	5(5)	3(3)	11%	4%	3%	
Cleavage stimulation factor subunit 3	B	-	-	125680	94	-	-	1(1)	-	-	1(1)	-	-	0%	-	-	KRY56722.1
Coatomer subunit alpha, partial	-	-	E	143838	-	-	124	-	-	2(2)	-	-	2(2)	-	-	1%	KRY52848.1
	-	-	F		-	-	87	-	-	1(1)	-	-	1(1)	-	-	0%	
	-	-	G		-	-	77	-	-	1(1)	-	-	1(1)	-	-	0%	
Coatomer subunit beta	D	D	D	168037	174	171	94	5(5)	3(3)	1(1)	5(5)	3(3)	1(1)	4%	3%	0%	KRY60662.1
	-	-	E		-	-	73	-	-	1(1)	-	-	1(1)	-	-	0%	
Coatomer subunit beta', partial	D	-	-	249362	116	-	-	3(3)	-	-	3(3)	-	-	1%	-	-	KRY52263.1
	-	-	E		-	-	140	-	-	6(6)	-	-	6(6)	-	-	2%	
Coatomer subunit delta	A	-	-	63168	69	-	-	1(1)	-	-	1(1)	-	-	2%	-	-	KRY53209.1
	C	-	C		129	-	87	3(3)	-	1(1)	3(3)	-	1(1)	6%	-	2%	
COP9 signalosome complex subunit	A	-	-	195133	66	-	-	1(1)	-	-	1(1)	-	-	0%	-	-	KRY61179.1
COP9 signalosome complex subunit 4, partial	-	A	-	47193	-	92	-	-	1(1)	-	-	1(1)	-	-	2%	-	KRY48695.1
Coronin-like protein cor-1	A	A	A	164005	94	99	80	1(1)	2(2)	1(1)	1(1)	2(2)	1(1)	0%	1%	0%	KRY60446.1
	-	-	B		-	-	97	-	-	1(1)	-	-	1(1)	-	-	0%	
	C	C	C		409	381	474	8(8)	6(6)	8(8)	8(8)	6(6)	7(7)	9%	7%	8%	
Cullin-associated nedd8-dissociated protein 1	-	-	G	145189	-	-	74	-	-	1(1)	-	-	1(1)	-	-	0%	KRY51041.1
Cuticlin-1	A	-	-	64300	68	-	-	1(1)	-	-	1(1)	-	-	1%	-	-	KRY58751.1
Cysteine and glycine-rich protein 1, partial	-	-	C	28836	-	-	99	-	-	1(1)	-	-	1(1)	-	-	6%	KRY56824.1
	-	-	E		-	-	104	-	-	1(1)	-	-	1(1)	-	-	6%	
Cysteine desulfurase, mitochondrial	B	B	B	49103	130	78	124	2(2)	2(2)	3(2)	2(2)	2(2)	3(2)	6%	7%	8%	KRY49825.1
Cytochrome b-c1 complex subunit 2, mitochondrial, partial	A	A	A	54732	243	120	187	4(4)	3(3)	2(2)	4(4)	3(3)	2(2)	11%	7%	5%	KRY49815.1
	B	B	B		140	183	106	2(2)	3(3)	1(1)	2(2)	3(3)	1(1)	5%	8%	2%	
Cytosol aminopeptidase	A	A	A	57910	88	76	89	2(2)	1(1)	2(2)	2(2)	1(1)	2(2)	3%	1%	3%	KRY54953.1

	B	B	B		139	90	219	4(4)	2(2)	7(7)	4(4)	2(2)	7(7)	8%	3%	15%	
	C	C	C		260	149	101	6(6)	4(4)	3(3)	6(6)	4(4)	3(3)	11%	8%	5%	
	D	D	D		179	161	71	5(5)	5(5)	1(1)	5(5)	5(5)	1(1)	13%	9%	2%	
	-	-	E		-	-	78	-	-	1(1)	-	-	1(1)	-	-	1%	
	-	-	F		-	-	103	-	-	4(4)	-	-	4(4)	-	-	8%	
	-	-	G		-	-	99	-	-	3(3)	-	-	3(3)	-	-	5%	
Cytosolic non-specific dipeptidase	C	-	-	58585	69	-	-	1(1)	-	-	1(1)	-	-	2%	-	-	KRY53197.1
Cytosolic purine 5'-nucleotidase	C	C	C	124481	157	69	75	4(4)	1(1)	1(1)	4(4)	1(1)	1(1)	3%	0%	0%	KRY47033.1
DDB1- and CUL4-associated factor 8	-	-	C	63244	-	-	85	-	-	1(1)	-	-	1(1)	-	-	2%	KRY56217.1
Deoxyribonuclease-2-alpha	B	B	B	39748	338	205	408	7(7)	5(5)	10(10)	7(7)	5(5)	9(9)	26%	16%	32%	KRY53116.1
	-	D	D	116176	-	667	249	-	15(15)	7(7)	-	10(10)	6(6)	-	13%	7%	KRY53318.1
	-	-	E		-	-	197	-	-	5(5)	-	-	5(5)	-	-	5%	
	-	-	F		-	-	113	-	-	2(2)	-	-	2(2)	-	-	2%	
Deoxyribonuclease-2-beta	-	B	-	40364	-	205	-	-	5(5)	-	-	5(5)	-	-	16%	-	KRY53117.1
Dihydrolipoyl dehydrogenase, mitochondrial	-	-	A	58994	-	-	91	-	-	2(2)	-	-	2(2)	-	-	6%	KRY53824.1
	B	-	B		94	-	156	4(4)	-	4(4)	4(4)	-	3(3)	6%	-	5%	
	-	-	C		-	-	67	-	-	2(1)	-	-	2(1)	-	-	3%	
	D	-	-		92	-	-	2(2)	-	-	2(2)	-	-	3%	-	-	
Dihydrolipoyllysine-residue acetyltransferase component of pyruvate dehydrogenase complex, mitochondrial	-	A	A	57120	-	85	59	-	1(1)	1(1)	-	1(1)	1(1)	-	1%	1%	KRY46167.1
	B	B	B		159	100	143	3(3)	3(3)	5(4)	3(3)	3(3)	5(4)	6%	4%	9%	
	C	-	-		84	-	-	1(1)	-	-	1(1)	-	-	1%	-	-	
	D	-	-		120	-	-	1(1)	-	-	1(1)	-	-	2%	-	-	
	-	-	E		-	-	125	-	-	1(1)	-	-	1(1)	-	-	2%	
Dihydrolipoyllysine-residue succinyltransferase component of 2-oxoglutarate dehydrogenase complex, mitochondrial	A	-	-	59790	144	-	-	3(3)	-	-	3(3)	-	-	6%	-	-	KRY53977.1
Dihydropyrimidinase-related protein 3	B	B	B	86548	100	106	139	2(2)	2(2)	3(3)	2(2)	2(2)	3(3)	2%	2%	4%	KRY48683.1
	C	C	C		80	134	108	1(1)	3(3)	2(2)	1(1)	3(3)	2(2)	1%	4%	3%	
Diphthine--ammonia ligase, partial	A	A	A	149815	104	67	106	1(1)	1(1)	2(2)	1(1)	1(1)	2(2)	0%	0%	1%	KRY47980.1
	B	B	B		104	112	204	1(1)	2(2)	4(4)	1(1)	2(2)	3(3)	0%	1%	3%	
	C	C	C		120	136	109	2(2)	3(3)	2(2)	2(2)	3(3)	2(2)	1%	1%	1%	
	D	D	D		557	412	267	11(11)	7(7)	5(5)	5(5)	3(3)	2(2)	7%	3%	2%	
	-	-	E		-	-	91	-	-	1(1)	-	-	1(1)	-	-	0%	
Disks large -like protein 1	-	C	-	103219	-	62	-	-	1(1)	-	-	1(1)	-	-	1%	-	KRY57378.1
DnaJ -like protein dnj-20	A	A	A	45223	148	172	162	2(2)	3(3)	3(3)	2(2)	3(3)	2(2)	7%	7%	7%	KRY45252.1
Dolichyl-diphosphooligosaccharide--protein glycosyltransferase 48 kDa subunit	-	-	B	85265	-	-	79	-	-	1(1)	-	-	1(1)	-	-	1%	KRY46770.1
Dolichyl-diphosphooligosaccharide--protein glycosyltransferase subunit 1	C	C	C	70258	87	73	123	3(3)	2(2)	5(5)	3(3)	2(2)	5(5)	4%	3%	9%	KRY49628.1
Dolichyl-diphosphooligosaccharide--protein glycosyltransferase subunit 2	C	C	C	91066	392	359	322	9(9)	13(12)	10(10)	8(8)	12(11)	10(10)	14%	19%	15%	KRY57029.1
Drebrin-like protein	-	-	A	121340	-	-	60	-	-	1(1)	-	-	1(1)	-	-	0%	KRY46208.1
Dual specificity mitogen-activated protein kinase kinase 3	-	C	C	47059	-	80	78	-	1(1)	1(1)	-	1(1)	1(1)	-	3%	3%	KRY61347.1
	-	-	E		-	-	69	-	-	1(1)	-	-	1(1)	-	-	3%	
Dymeclin	-	-	C	86333	-	-	78	-	-	1(1)	-	-	1(1)	-	-	1%	KRY55372.1
Dynamin	-	D	D	99981	-	140	108	-	8(8)	2(2)	-	8(8)	2(2)	-	9%	2%	KRY57399.1
Dynein heavy chain, cytoplasmic	A	A	A	569784	146	112	128	4(4)	1(1)	3(3)	4(4)	1(1)	3(3)	0%	0%	0%	KRY59545.1
	-	-	C		-	-	65	-	-	1(1)	-	-	1(1)	-	-	0%	
	-	-	G	568220	-	-	79	-	-	1(1)	-	-	1(1)	-	-	0%	KRY59544.1
E3 ubiquitin-protein ligase HUWE1	-	-	E	497021	-	-	69	-	-	1(1)	-	-	1(1)	-	-	0%	KRY53411.1

	-	-	G		-	-	75	-	-	1(1)	-	-	1(1)	-	-	0%	
E3 ubiquitin-protein ligase listerin	-	-	G	206888	-	-	76	-	-	1(1)	-	-	1(1)	-	-	0%	KRY47160.1
E3 ubiquitin-protein ligase UBR5	D	-	-	416466	127	-	-	1(1)	-	-	1(1)	-	-	0%	-	-	KRY58314.1
Ectonucleotide pyrophosphatase/phosphodiesterase C27A7.1	D	-	-	89968	73	-	-	1(1)	-	-	1(1)	-	-	1%	-	-	KRY49188.1
Elongation factor 1-alpha	-	A	-	54609	-	631	-	-	18(18)	-	-	9(9)	-	-	29%	-	KRY54798.1
	B	-	-		952	-	-	23(23)	-	-	12(12)	-	-	39%	-	-	
	-	-	C		-	-	261	-	-	9(9)	-	-	7(7)	-	-	19%	
	-	-	E		-	-	219	-	-	5(5)	-	-	4(4)	-	-	11%	
	-	-	F		-	-	136	-	-	3(3)	-	-	3(3)	-	-	6%	
	-	-	G		-	-	207	-	-	5(5)	-	-	4(4)	-	-		
Elongation factor 1-gamma, partial	B	B	B	52493	77	92	79	1(1)	1(1)	1(1)	1(1)	1(1)	1(1)	2%	3%	2%	KRY58663.1
Elongation factor G, mitochondrial	-	B	-	101076	-	95	-	-	1(1)	-	-	1(1)	-	-	1%	-	KRY55228.1
	C	C	C		57	106	98	1(1)	2(2)	2(2)	1(1)	2(2)	2(2)	1%	2%	2%	
	D	D	-		87	66	-	1(1)	1(1)	-	1(1)	1(1)	-	1%	1%	-	
Elongation factor Ts, mitochondrial	A	-	A	71640	73	-	60	2(2)	-	1(1)	2(2)	-	1(1)	3%	-	1%	KRY51161.1
Elongation factor Tu, mitochondrial, partial	B	-	-	55743	88	-	-	2(2)	-	-	2(2)	-	-	4%	-	-	KRY60374.1
	-	-	E		-	-	62	-	-	1(1)	-	-	1(1)	-	-	2%	
Endoplasmic oxidoreductin-1, partial	-	-	B	56076	-	-	88	-	-	1(1)	-	-	1(1)	-	-	2%	KRY50205.1
Endoplasmic reticulum resident protein 44	A	A	A	50147	81	100	106	1(1)	1(1)	1(1)	1(1)	1(1)	1(1)	2%	2%	2%	KRY50742.1
	B	B	B		168	281	353	5(5)	8(7)	10(10)	4(4)	5(5)	5(5)	13%	18%	18%	
	D	D	-		59	69	-	1(1)	1(1)	-	1(1)	1(1)	-	2%	2%	-	
Endoplasmin, partial	A	A	A	95730	352	224	247	7(7)	6(6)	7(7)	6(6)	5(5)	6(6)	9%	8%	10%	KRY54651.1
	-	C	-		-	292	-	-	7(7)	-	-	7(7)	-	-	11%	-	
	D	-	D		1925	-	390	53(53)	-	19(18)	35(35)	-	17(17)	44%	-	14%	
Enoyl-CoA delta isomerase 2, mitochondrial	A	A	A	47790	144	92	98	5(5)	3(2)	2(2)	4(4)	3(2)	2(2)	11%	8%	5%	KRY48829.1
Enoyl-CoA hydratase, mitochondrial	A	-	A	34086	147	-	98	3(3)	-	1(1)	3(3)	-	1(1)	13%	-	4%	KRY60485.1
	-	-	B		-	-	85	-	-	1(1)	-	-	1(1)	-	-	4%	
	C	C	C		90	102	129	3(3)	3(3)	3(3)	3(3)	3(3)	2(2)	15%	12%	7%	
	D	D	-		174	104	-	2(2)	2(2)	-	2(2)	2(2)	-	9%	7%	-	
	-	-	E		-	-	69	-	-	1(1)	-	-	1(1)	-	-	3%	
Epsin-2	C	-	-	57740	65	-	-	1(1)	-	-	1(1)	-	-	2%	-	-	KRY61379.1
Eukaryotic initiation factor 4A, partial	A	A	A	252102	268	208	160	7(7)	5(5)	3(3)	6(6)	5(5)	3(3)	3%	2%	1%	KRY53637.1
	-	C	C		-	83	94	-	1(1)	1(1)	-	1(1)	1(1)	-	0%	0%	
	D	C	-		85	116	-	1(1)	2(2)	-	1(1)	2(2)	-	0%	1%	-	
	-	-	E		-	-	73	-	-	1(1)	-	-	1(1)	-	-	0%	
Eukaryotic initiation factor 4A-III	B	B	B	123600	133	163	215	4(4)	6(6)	7(7)	4(4)	6(6)	7(7)	4%	4%	6%	KRY49326.1
	C	C	C		165	65	127	3(3)	1(1)	2(1)	3(3)	1(1)	2(1)	3%	1%	2%	
Eukaryotic peptide chain release factor GTP-binding subunit ERF3A, partial	C	-	-	45762	156	-	-	2(2)	-	-	2(2)	-	-	8%	-	-	KRY52451.1
Eukaryotic peptide chain release factor GTP-binding subunit ERF3B	C	-	-	65245	156	-	-	2(2)	-	-	2(2)	-	-	6%	-	-	KRY52453.1
Eukaryotic peptide chain release factor subunit 1	-	-	B	49470	-	-	89	-	-	1(1)	-	-	1(1)	-	-	2%	KRY49983.1
Eukaryotic translation initiation factor 3 subunit C	-	C	-	122944	-	71	-	-	1(1)	-	-	1(1)	-	-	0%	-	KRY47525.1
	-	D	-		-	62	-	-	1(1)	-	-	1(1)	-	-	0%	-	
	-	-	F		-	-	63	-	-	1(1)	-	-	1(1)	-	-	0%	
	-	-	G		-	-	77	-	-	2(2)	-	-	2(2)	-	-	1%	
Eukaryotic translation initiation factor 3 subunit L	C	C	C	65995	145	117	165	5(5)	5(5)	5(5)	5(5)	5(5)	5(5)	9%	7%	9%	KRY53932.1
Eukaryotic translation initiation factor 4 gamma 1	-	-	E	156176	-	-	114	-	-	1(1)	-	-	1(1)	-	-	1%	KRY55755.1

Eukaryotic translation initiation factor 5B, partial	D	-	-	129310	79	-	-	1(1)	-	-	1(1)	-	-	0%	-	-	KRY59270.1
Exportin-2	D	D	D	123252	92	95	92	1(1)	1(1)	1(1)	1(1)	1(1)	1(1)	1%	1%	1%	KRY48592.1
	-	-	G		-	-	88	-	-	1(1)	-	-	1(1)	-	-	1%	
Extended synaptotagmin-2, partial	-	-	C	205677	-	-	133	-	-	2(2)	-	-	2(2)	-	-	1%	KRY50391.1
Far upstream element-binding protein 2	A	-	A	75769	85	-	85	1(1)	-	2(2)	1(1)	-	2(2)	1%	-	3%	KRY57083.1
	B	B	B		102	69	190	2(1)	2(1)	5(5)	2(1)	2(1)	4(4)	3%	3%	6%	
	C	C	-		246	238	-	7(7)	12(10)	-	7(7)	11(10)	-	9%	15%	-	
Filamin-A, partial	-	A	-	259917	-	66	-	-	1(1)	-	-	1(1)	-	-	0%	-	KRY59624.1
	-	C	C		-	121	126	-	2(2)	2(2)	-	2(2)	2(2)	-	1%	1%	
	D	D	D		505	154	89	12(12)	3(3)	1(1)	12(12)	3(3)	1(1)	8%	1%	0%	
	-	-	E		-	-	114	-	-	2(2)	-	-	2(2)	-	-	1%	
	-	-	F		-	-	63	-	-	1(1)	-	-	1(1)	-	-	0%	
	-	-	G		-	-	158	-	-	3(3)	-	-	3(3)	-	-	1%	
Fragile X mental retardation protein 1 -like protein B	C	C	-	64707	117	85	-	2(2)	1(1)	-	2(2)	1(1)	-	4%	1%	-	KRY58060.1
Fragile X mental retardation syndrome-related protein 1	-	-	A	48548	-	-	64	-	-	1(1)	-	-	1(1)	-	-	2%	KRY58061.1
Fructose-bisphosphate aldolase 1, partial	A	A	A	44597	1124	753	870	22(22)	13(13)	16(16)	14(14)	9(9)	11(11)	50%	36%	44%	KRY52953.1
	B	B	B		109	357	315	2(2)	8(8)	6(5)	2(2)	7(7)	5(4)	8%	28%	22%	
	C	C	C		212	259	187	3(3)	5(5)	3(3)	3(3)	4(4)	3(3)	16%	12%	9%	
	D	D	D		156	197	173	2(2)	4(4)	3(2)	2(2)	4(4)	3(2)	10%	11%	10%	
	-	-	E		-	-	143	-	-	3(3)	-	-	3(3)	-	-	9%	
	-	-	F		-	-	94	-	-	1(1)	-	-	1(1)	-	-	3%	
	-	-	G		-	-	162	-	-	2(2)	-	-	2(2)	-	-	6%	
GDP-fucose protein O-fucosyltransferase 1	A	A	-	55623	112	83	-	2(2)	1(1)	-	2(2)	1(1)	-	6%	2%	-	KRY61128.1
Gelsolin, cytoplasmic, partial	D	-	-	62573	96	-	-	1(1)	-	-	1(1)	-	-	2%	-	-	KRY58897.1
General vesicular transport factor, partial	-	D	-	118001	-	62	-	-	1(1)	-	-	1(1)	-	-	1%	-	KRY58955.1
Gephyrin	-	C	C	70317	-	99	77	-	2(2)	1(1)	-	2(2)	1(1)	-	3%	1%	KRY53926.1
Glioma pathogenesis-related protein 1	A	A	A	54399	67	80	148	1(1)	1(1)	1(1)	1(1)	1(1)	1(1)	3%	3%	3%	KRY45995.1
	-	B	B		-	81	64	-	1(1)	1(1)	-	1(1)	1(1)	-	3%	3%	
	-	C	C		-	92	76	-	2(2)	1(1)	-	2(2)	1(1)	-	3%	3%	
	D	D	-		58	101	-	1(1)	1(1)	-	1(1)	1(1)	-	1%	3%	-	
Glucose-6-phosphate 1-dehydrogenase	C	C	C	61532	101	109	97	3(3)	2(2)	2(2)	3(3)	2(2)	2(2)	6%	4%	4%	KRY54351.1
Glucose-6-phosphate isomerase	B	B	B	65882	108	167	223	2(2)	3(3)	4(4)	2(2)	3(3)	4(4)	4%	7%	10%	KRY50483.1
	C	C	C		253	244	160	6(5)	6(6)	2(2)	6(5)	6(6)	2(2)	12%	13%	5%	
	D	D	-		88	66	-	1(1)	1(1)	-	1(1)	1(1)	-	2%	2%	-	
Glucosidase 2 subunit beta	C	C	-	59343	76	68	-	1(1)	1(1)	-	1(1)	1(1)	-	2%	2%	-	KRY56172.1
Glucosylceramidase	C	C	C	61978	188	143	125	5(5)	3(3)	3(3)	5(5)	3(3)	3(3)	10%	5%	6%	KRY50060.1
Glutamate dehydrogenase 2, mitochondrial	A	-	A	56781	123	-	102	2(2)	-	3(3)	2(2)	-	3(3)	5%	-	6%	KRY47480.1
	-	-	G		-	-	76	-	-	1(1)	-	-	1(1)	-	-	2%	
	-	-	F	60132	-	-	78	-	-	1(1)	-	-	1(1)	-	-	2%	KRY47481.1
Glutamate dehydrogenase, mitochondrial, partial	-	-	D	57060	-	-	86	-	-	1(1)	-	-	1(1)	-	-	3%	KRY47482.1
	-	-	F		-	-	78	-	-	1(1)	-	-	1(1)	-	-	2%	
glutathione-S-transferase	-	A	A	24198	-	64	64	-	1(0)	1(1)	-	1(0)	1(1)	-	5%	5%	ABA42916.1
	-	-	B		-	-	65	-	-	1(1)	-	-	1(1)	-	-	5%	
	C	C	C		70	72	63	1(1)	2(1)	1(1)	1(1)	2(1)	1(1)	5%	9%	5%	
	-	D	-		-	64	-	-	1(1)	-	-	1(1)	-	-	5%	-	
	-	-	E		-	-	69	-	-	1(1)	-	-	1(1)	-	-	5%	
	-	-	G		-	-	71	-	-	1(1)	-	-	1(1)	-	-	5%	
Glyceraldehyde-3-phosphate dehydrogenase 1	A	A	A	37666	125	210	167	5(4)	7(7)	5(5)	5(4)	7(7)	5(5)	17%	25%	19%	KRY59635.1
	B	B	B		86	233	289	2(2)	6(5)	7(7)	2(2)	6(5)	7(7)	7%	21%	26%	

	C	C	C		387	271	281	9(9)	8(8)	6(6)	8(8)	8(8)	6(6)	33%	30%	24%	
	D	D	D		479	374	149	12(12)	10(10)	3(3)	10(10)	8(8)	3(3)	40%	30%	12%	
	-	-	E		-	-	206	-	-	5(4)	-	-	5(4)	-	-	19%	
	-	-	F		-	-	82	-	-	1(1)	-	-	1(1)	-	-	2%	
	-	-	G		-	-	155	-	-	3(3)	-	-	3(3)	-	-	8%	
Glycogen debranching enzyme, partial	-	C	C	200494	-	95	72	-	1(1)	1(1)	-	1(1)	1(1)	-	0%	0%	KRY52323.1
	D	D	-		390	150	-	10(10)	4(4)	-	10(10)	4(4)	-	7%	2%	-	
	-	-	E		-	-	530	-	-	12(11)	-	-	11(10)	-	-	7%	
	-	-	F		-	-	568	-	-	17(17)	-	-	16(16)	-	-	11%	
	-	-	G		-	-	275	-	-	9(8)	-	-	9(8)	-	-	6%	
Glycogen phosphorylase, partial	A	A	-	175168	107	132	-	2(2)	3(3)	-	2(2)	3(3)	-	1%	2%	-	KRY47089.1
	-	B	B		-	117	84	-	2(2)	3(3)	-	2(2)	3(3)	-	2%	2%	
	-	C	C		-	231	282	-	8(8)	10(9)	-	8(8)	9(8)	-	6%	7%	
	D	D	D		2144	1604	912	57(57)	45(45)	28(27)	30(30)	26(26)	22(22)	23%	20%	19%	
	-	-	E		-	-	675	-	-	18(18)	-	-	15(15)	-	-	12%	
Glycogenin-1	A	A	A	41089	158	118	133	4(4)	3(3)	3(3)	3(3)	2(2)	3(3)	10%	6%	10%	KRY61196.1
	B	B	B		80	98	67	1(1)	3(3)	1(1)	1(1)	3(3)	1(1)	3%	10%	3%	
Glycylpeptide N-tetradecanoyltransferase 2, partial	-	B	B	125590	-	83	104	-	1(1)	2(2)	-	1(1)	2(2)	-	1%	2%	KRY51777.1
GMP synthase [glutamine-hydrolyzing], partial	C	-	-	79440	60	-	-	1(1)	-	-	1(1)	-	-	1%	-	-	KRY52958.1
	-	-	G		-	-	69	-	-	1(1)	-	-	1(1)	-	-	1%	
Golgi apparatus protein 1 -like protein, partial	D	D	-	136466	95	64	-	1(1)	1(1)	-	1(1)	1(1)	-	0%	0%	-	KRY52646.1
	-	-	E		-	-	475	-	-	12(12)	-	-	12(12)	-	-	13%	
Guanine nucleotide-binding protein subunit beta-2-like 1	-	C	C	39904	-	155	96	-	3(3)	1(1)	-	3(3)	1(1)	-	10%	4%	KRY55875.1
	D	-	D		84	-	86	1(1)	-	1(1)	1(1)	-	1(1)	4%	-	3%	
	-	-	E		-	-	79	-	-	1(1)	-	-	1(1)	-	-	3%	
	-	-	F		-	-	72	-	-	1(1)	-	-	1(1)	-	-	3%	
	-	-	G		-	-	84	-	-	1(1)	-	-	1(1)	-	-	3%	
H/ACA ribonucleoprotein complex subunit 4	-	B	-	51067	-	68	-	-	1(1)	-	-	1(1)	-	-	1%	-	KRY53301.1
Heat shock 70 kDa protein 13	C	-	-	50433	67	-	-	1(1)	-	-	1(1)	-	-	2%	-	-	KRY49991.1
Heat shock 70 kDa protein 4L	-	-	B	101361	-	-	91	-	-	1(1)	-	-	1(1)	-	-	1%	KRY51771.1
	-	-	E		-	-	115	-	-	5(5)	-	-	5(5)	-	-	7%	
	-	-	G		-	-	72	-	-	1(1)	-	-	1(1)	-	-	1%	
Heat shock cognate 71 kDa protein, partial	A	A	A	131529	633	636	569	19(18)	21(19)	18(18)	17(16)	16(14)	16(16)	17%	17%	16%	KRY58599.1
	B	B	B		175	308	165	3(3)	7(7)	6(6)	3(3)	7(7)	6(6)	2%	7%	5%	
	C	C	C		1183	1014	1036	40(38)	36(34)	33(32)	28(27)	24(22)	24(23)	25%	21%	24%	
	D	D	D		727	505	218	20(20)	14(14)	8(8)	18(18)	13(13)	8(8)	17%	13%	8%	
	-	-	E		-	-	421	-	-	11(11)	-	-	10(10)	-	-	10%	
	-	-	F		-	-	437	-	-	12(12)	-	-	11(11)	-	-	11%	
	-	-	G		-	-	380	-	-	10(10)	-	-	9(9)	-	-	9%	
heat shock protein 70	B	B	-	71860	175	308	-	3(3)	7(7)	-	3(3)	7(7)	-	5%	13%	-	CAA73574.1
Heat shock protein 83, partial	A	A	A	91707	207	193	225	5(5)	7(7)	8(8)	5(5)	7(7)	8(8)	6%	10%	10%	KRY48983.1
	B	B	B		362	369	441	12(12)	15(14)	18(17)	11(11)	13(13)	12(12)	15%	19%	15%	
	C	C	C		562	446	429	19(19)	12(12)	17(15)	19(19)	11(11)	15(13)	24%	16%	19%	
	D	D	D		551	594	345	13(13)	17(17)	9(9)	11(11)	15(15)	9(9)	16%	22%	13%	
	-	-	E		-	-	456	-	-	13(13)	-	-	12(12)	-	-	17%	
	-	-	F		-	-	320	-	-	10(9)	-	-	10(9)	-	-	14%	
	-	-	G		-	-	424	-	-	15(15)	-	-	14(14)	-	-	20%	
Hexokinase	A	-	A	56783	241	-	140	3(3)	-	3(3)	2(2)	-	2(2)	6%	-	7%	KRY57386.1
	B	B	B		262	464	540	8(8)	12(11)	14(14)	7(7)	9(8)	12(12)	16%	28%	34%	

	C	C	-		92	86	-	1(1)	1(1)	-	1(1)	1(1)	-	3%	2%	-	
	-	D	-		-	59	-	-	1(1)	-	-	1(1)	-	-	1%	-	
Hexokinase type 2, partial	B	B	B	52657	98	75	139	1(1)	1(1)	4(3)	1(1)	1(1)	3(3)	2%	3%	9%	KRY53884.1
Histone-arginine methyltransferase CARM1	C	-	-	69282	71	-	-	1(1)	-	-	1(1)	-	-	1%	-	-	KRY59724.1
Histone-binding protein RBBP7	-	B	B	48666	-	60	78	-	1(1)	1(1)	-	1(1)	1(1)	-	3%	3%	KRY54520.1
Histone-lysine N-methyltransferase EHMT2, partial	A	A	A	40953	161	142	158	3(3)	2(2)	3(3)	3(3)	2(2)	3(3)	15%	7%	11%	KRY52481.1
Hsp90 co-chaperone Cdc37	A	A	-	42726	85	81	-	1(1)	1(1)	-	1(1)	1(1)	-	4%	2%	-	KRY60145.1
	B	-	-		70	-	-	1(1)	-	-	1(1)	-	-	2%	-	-	
hypothetical protein T03_10292	-	-	E	70667	-	-	64	-	-	1(1)	-	-	1(1)	-	-	1%	KRY52704.1
hypothetical protein T03_11006	B	-	B	163737	136	-	146	3(3)	-	5(5)	3(3)	-	5(5)	2%	-	3%	KRY56142.1
	-	-	C		-	-	80	-	-	1(1)	-	-	1(1)	-	-	0%	
hypothetical protein T03_13827	A	A	A	13483	84	86	109	1(1)	1(1)	2(2)	1(1)	1(1)	2(2)	9%	10%	19%	KRY56487.1
	B	B	B		71	79	82	1(1)	1(1)	1(1)	1(1)	1(1)	1(1)	10%	10%	10%	
	-	C	-		-	116	-	-	2(2)	-	-	2(2)	-	-	19%	-	
	-	-	F		-	-	78	-	-	1(1)	-	-	1(1)	-	-	10%	
hypothetical protein T03_14678	A	-	-	55770	88	-	-	1(1)	-	-	1(1)	-	-	2%	-	-	KRY57784.1
	B	B	B		71	116	133	1(1)	2(2)	2(2)	1(1)	2(2)	2(2)	1%	4%	4%	
	-	-	C		-	-	735	-	-	20(20)	-	-	13(13)	-	-	27%	
	-	D	D		-	74	94	-	1(1)	2(2)	-	1(1)	2(2)	-	3%	4%	
	-	-	E		-	-	147	-	-	3(3)	-	-	3(3)	-	-	9%	
	-	-	F		-	-	65	-	-	1(1)	-	-	1(1)	-	-	3%	
	-	-	G		-	-	82	-	-	1(1)	-	-	1(1)	-	-	1%	
hypothetical protein T03_14974, partial	-	-	B	43617	-	-	78	-	-	1(1)	-	-	1(1)	-	-	3%	KRY46929.1
	C	C	-		95	76	-	2(2)	1(1)	-	2(2)	1(1)	-	6%	3%	-	
	D	D	-		98	61	-	1(1)	1(1)	-	1(1)	1(1)	-	3%	3%	-	
hypothetical protein T03_16806, partial	A	-	-	30897	56	-	-	1(1)	-	-	1(1)	-	-	5%	-	-	KRY50505.1
hypothetical protein T03_17187	A	A	A	46783	1259	671	814	24(23)	14(14)	15(15)	18(17)	11(11)	11(11)	56%	39%	43%	KRY50178.1
	B	B	B		832	879	895	14(14)	17(17)	19(18)	9(9)	12(12)	14(13)	35%	45%	49%	
	C	C	C		288	246	197	6(6)	5(5)	3(3)	6(6)	4(4)	2(2)	23%	11%	9%	
hypothetical protein T03_17805, partial	-	C	-	18630	-	63	-	-	1(1)	-	-	1(1)	-	-	6%	-	KRY53056.1
hypothetical protein T03_3884	-	D	-	26017	-	59	-	-	1(1)	-	-	1(1)	-	-	3%	-	KRY58587.1
	-	-	F		-	-	64	-	-	1(1)	-	-	1(1)	-	-	3%	
hypothetical protein T03_3917, partial	C	-	C	46109	86	-	69	1(1)	-	1(1)	1(1)	-	1(1)	3%	-	3%	KRY54907.1
hypothetical protein T03_4197	-	B	B	88736	-	74	79	-	1(1)	1(1)	-	1(1)	1(1)	-	1%	1%	KRY53644.1
	D	D	D		1077	521	159	28(27)	17(17)	6(6)	23(22)	16(16)	6(6)	33%	25%	9%	
	-	-	E		-	-	95	-	-	1(1)	-	-	1(1)	-	-	4%	
hypothetical protein T03_4258, partial	-	-	D	32222	-	-	70	-	-	1(1)	-	-	1(1)	-	-	4%	KRY53223.1
	-	-	E		-	-	67	-	-	1(1)	-	-	1(1)	-	-	4%	
hypothetical protein T03_539	A	A	A	55965	1134	756	785	26(26)	20(20)	19(19)	16(16)	11(11)	12(12)	30%	19%	19%	KRY55455.1
	B	B	B		227	364	242	5(5)	9(9)	5(4)	4(4)	8(8)	4(3)	8%	15%	9%	
	-	D	-		-	73	-	-	1(1)	-	-	1(1)	-	-	2%	-	
hypothetical protein T03_7457	C	-	C	49230	111	-	94	1(1)	-	1(1)	1(1)	-	1(1)	3%	-	3%	KRY48668.1
hypothetical protein T03_7459	-	B	-	13934	-	120	-	-	1(1)	-	-	1(1)	-	-	13%	-	KRY60639.1
	-	C	C		-	124	124	-	1(1)	1(1)	-	1(1)	1(1)	-	13%	13%	
	D	D	-		124	117	-	1(1)	1(1)	-	1(1)	1(1)	-	13%	13%	-	
	-	-	E		-	-	93	-	-	1(1)	-	-	1(1)	-	-	13%	
	-	-	F		-	-	103	-	-	1(1)	-	-	1(1)	-	-	13%	
	-	-	G		-	-	90	-	-	1(1)	-	-	1(1)	-	-	13%	
hypothetical protein T03_7883	D	D	D	117012	195	210	102	4(4)	3(3)	1(1)	4(4)	2(2)	1(1)	4%	2%	0%	KRY46989.1

	-	-	E		-	-	220	-	-	5(5)	-	-	4(4)	-	-	4%	
	-	-	F		-	-	99	-	-	3(2)	-	-	3(2)	-	-	2%	
hypothetical protein T03_7939	A	A	A	64129	68	137	79	1(1)	2(2)	1(1)	1(1)	2(2)	1(1)	1%	4%	1%	KRY48964.1
	-	-	B		-	-	70	-	-	1(1)	-	-	1(1)	-	-	1%	
	C	-	-		98	-	-	2(2)	-	-	2(2)	-	-	4%	-	-	
	-	D	-		-	61	-	-	1(1)	-	-	1(1)	-	-	1%	-	
hypothetical protein T03_8666	A	A	A	55679	82	67	102	1(1)	2(2)	3(3)	1(1)	2(2)	3(3)	2%	7%	14%	KRY58914.1
	B	B	-		130	228	-	3(3)	7(7)	-	3(3)	7(7)	-	7%	9%	-	
	C	-	-		107	-	-	4(4)	-	-	4(4)	-	-	7%	-	-	
hypothetical protein T03_8694	A	A	A	40952	144	188	155	5(5)	8(7)	7(6)	5(5)	8(7)	6(5)	14%	22%	17%	KRY46541.1
	B	B	B		63	78	90	1(1)	1(1)	3(3)	1(1)	1(1)	3(3)	3%	3%	9%	
	-	-	C		-	-	83	-	-	1(1)	-	-	1(1)	-	-	3%	
	D	D	-		78	68	-	2(2)	2(1)	-	2(2)	2(1)	-	5%	6%	-	
hypothetical protein T03_9489	C	C	C	19940	72	68	65	1(1)	1(1)	1(1)	1(1)	1(1)	1(1)	5%	5%	5%	KRY46491.1
	D	D	-		65	68	-	1(1)	1(1)	-	1(1)	1(1)	-	5%	5%	-	
	-	-	E		-	-	71	-	-	1(1)	-	-	1(1)	-	-	5%	
hypothetical protein T03_9928	C	-	C	82569	86	-	69	1(1)	-	1(1)	1(1)	-	1(1)	1%	-	1%	KRY47039.1
	-	-	E		-	-	62	-	-	1(1)	-	-	1(1)	-	-	1%	
hypothetical protein T03_9976	A	A	-	69321	92	119	-	2(2)	3(3)	-	2(2)	3(3)	-	3%	5%	-	KRY53902.1
	B	B	B		79	109	89	1(1)	3(3)	1(1)	1(1)	3(3)	1(1)	2%	8%	1%	
	-	-	F		-	-	77	-	-	1(1)	-	-	1(1)	-	-	1%	
Hypoxia up-regulated protein 1, partial	D	-	-	108762	188	-	-	3(3)	-	-	3(3)	-	-	4%	-	-	KRY49848.1
	-	-	F		-	-	206	-	-	5(5)	-	-	5(5)	-	-	6%	
Ig-like and fibronectin type-III domain-containing protein C25G4.10	C	C	-	179761	191	107	-	3(2)	2(2)	-	3(2)	2(2)	-	2%	1%	-	KRY58616.1
	-	-	E		-	-	62	-	-	1(1)	-	-	1(1)	-	-	0%	
	-	-	F		-	-	62	-	-	1(1)	-	-	1(1)	-	-	0%	
Importin subunit beta-1, partial	D	D	-	101582	203	103	-	3(3)	1(1)	-	3(3)	1(1)	-	5%	1%	-	KRY56880.1
	-	-	E		-	-	68	-	-	1(1)	-	-	1(1)	-	-	0%	
Importin-7	-	-	E	158791	-	-	73	-	-	1(1)	-	-	1(1)	-	-	0%	KRY59671.1
Importin-8	-	-	E	157915	-	-	73	-	-	1(1)	-	-	1(1)	-	-	0%	KRY59672.1
Innexin unc-9	-	D	-	48936	-	66	-	-	1(1)	-	-	1(1)	-	-	2%	-	KRY59455.1
Innexin-12	B	B	B	55187	84	92	105	3(3)	3(2)	4(3)	3(3)	3(2)	4(3)	6%	6%	9%	KRY59456.1
Inositol monophosphatase ttx-7, partial	-	-	B	32017	-	-	62	-	-	1(1)	-	-	1(1)	-	-	4%	KRY54643.1
	-	C	-		-	101	-	-	1(1)	-	-	1(1)	-	-	3%	-	
Insulin-degrading enzyme	C	-	-	117599	66	-	-	1(1)	-	-	1(1)	-	-	0%	-	-	KRY56124.1
	-	-	D		-	-	132	-	-	3(3)	-	-	3(3)	-	-	3%	
	-	-	E		-	-	82	-	-	2(2)	-	-	2(2)	-	-	1%	
Integrin beta pat-3	-	-	E	97116	-	-	109	-	-	2(2)	-	-	2(2)	-	-	2%	KRY53462.1
Intermediate filament protein B, partial	A	A	-	71689	114	94	-	3(3)	2(2)	-	3(3)	2(2)	-	4%	2%	-	KRY59373.1
	B	B	B		165	199	192	7(7)	10(9)	12(10)	7(7)	10(9)	11(10)	10%	15%	14%	
	C	C	C		827	670	983	37(35)	29(29)	42(42)	26(26)	21(21)	27(27)	33%	33%	37%	
	-	D	-		-	82	-	-	1(1)	-	-	1(1)	-	-	1%	-	
	-	-	G		-	-	80	-	-	2(2)	-	-	2(2)	-	-	3%	
Intermediate filament protein ifa-1	A	A	A	73429	307	289	401	13(13)	13(13)	15(13)	12(12)	12(12)	13(11)	17%	20%	20%	KRY45949.1
	B	B	B		397	436	536	18(18)	17(17)	23(23)	17(17)	15(15)	20(20)	23%	20%	30%	
	D	D	D		281	229	95	7(6)	10(9)	2(2)	7(6)	10(9)	2(2)	14%	17%	2%	
	-	-	E		-	-	198	-	-	7(6)	-	-	6(5)	-	-	9%	
	-	-	F		-	-	125	-	-	6(6)	-	-	6(6)	-	-	8%	
Iron-sulfur cluster assembly enzyme ISCU, mitochondrial	-	-	B	202951	-	-	76	-	-	1(1)	-	-	1(1)	-	-	0%	KRY49130.1

Isochorismatase domain-containing protein 2, mitochondrial	-	C	C	19834	-	80	78	-	1(1)	1(1)	-	1(1)	1(1)	-	8%	8%	KRY61349.1
	-	-	E		-	-	69	-	-	1(1)	-	-	1(1)	-	-	8%	
Isoleucine--tRNA ligase, cytoplasmic	-	-	E	143414	-	-	110	-	-	2(2)	-	-	2(2)	-	-	1%	KRY51016.1
	-	-	F		-	-	64	-	-	1(1)	-	-	1(1)	-	-	0%	
	-	-	G		-	-	78	-	-	1(1)	-	-	1(1)	-	-	0%	
Isopentenyl-diphosphate Delta-isomerase II, partial	-	B	-	41386	-	64	-	-	1(1)	-	-	1(1)	-	-	2%	-	KRY59814.1
	C	C	-		79	67	-	1(1)	1(1)	-	1(1)	1(1)	-	2%	2%	-	
	D	-	-		84	-	-	1(1)	-	-	1(1)	-	-	2%	-	-	
	-	-	E		-	-	85	-	-	1(1)	-	-	1(1)	-	-	2%	
	-	-	G		-	-	75	-	-	1(1)	-	-	1(1)	-	-	2%	
J5 protein, partial	A	-	A	41174	77	-	94	1(1)	-	1(1)	1(1)	-	1(1)	2%	-	2%	AAD42227.1
	-	-	B		-	-	77	-	-	1(1)	-	-	1(1)	-	-	3%	
KDEL motif-containing protein 1	-	-	C	58465	-	-	86	-	-	1(1)	-	-	1(1)	-	-	2%	KRY54258.1
KDEL motif-containing protein 2	C	-	C	59198	57	-	86	1(0)	-	1(1)	1(0)	-	1(1)	1%	-	2%	KRY54257.1
KH domain-containing, RNA-binding, signal transduction-associated protein 2	-	-	B	49642	-	-	112	-	-	3(2)	-	-	3(2)	-	-	7%	KRY48053.1
Kinesin heavy chain	-	-	C	115384	-	-	89	-	-	1(1)	-	-	1(1)	-	-	1%	KRY58465.1
	D	D	-		96	78	-	2(2)	1(1)	-	2(2)	1(1)	-	2%	0%	-	
	-	-	G		-	-	66	-	-	1(1)	-	-	1(1)	-	-	0%	
Kynurenine--oxoglutarate transaminase 3	A	A	A	60100	241	209	227	5(5)	7(6)	4(4)	4(4)	7(6)	4(4)	11%	16%	12%	KRY54139.1
	-	B	-		-	61	-	-	1(1)	-	-	1(1)	-	-	2%	-	
L-2-hydroxyglutarate dehydrogenase, mitochondrial	A	A	A	47961	356	193	144	8(8)	4(4)	3(3)	7(7)	4(4)	3(3)	23%	15%	9%	KRY56323.1
Laminin subunit alpha-2, partial	-	-	E	358743	-	-	64	-	-	1(1)	-	-	1(1)	-	-	0%	KRY58196.1
LETM1 and EF-hand domain-containing protein 1, mitochondrial	A	A	-	118848	92	69	-	2(2)	1(1)	-	2(2)	1(1)	-	2%	1%	-	KRY56766.1
	-	B	-		-	87	-	-	1(1)	-	-	1(1)	-	-	1%	-	
Leucine-rich PPR motif-containing protein, mitochondrial	-	-	E	154713	-	-	112	-	-	4(4)	-	-	4(4)	-	-	2%	KRY57360.1
	-	-	G		-	-	66	-	-	1(1)	-	-	1(1)	-	-	0%	
Leucine-rich repeat-containing protein 15	B	-	-	77180	62	-	-	1(1)	-	-	1(1)	-	-	2%	-	-	KRY61264.1
	C	-	-	68831	63	-	-	1(1)	-	-	1(1)	-	-	1%	-	-	KRY60652.1
	D	D	D		284	85	68	10(8)	5(4)	1(0)	9(8)	5(4)	1(0)	18%	8%	1%	
	-	-	E		-	-	65	-	-	1(1)	-	-	1(1)	-	-	1%	
Leucine-rich repeat-containing protein 4B, partial	D	D	-	92627	197	139	-	2(2)	2(2)	-	2(2)	2(2)	-	3%	3%	-	KRY56692.1
Leucine-rich repeat-containing protein let-4	B	-	-	81991	62	-	-	1(1)	-	-	1(1)	-	-	1%	-	-	KRY61263.1
Leucine--tRNA ligase, cytoplasmic	D	-	-	135420	99	-	-	1(1)	-	-	1(1)	-	-	1%	-	-	KRY54245.1
	-	-	E		-	-	76	-	-	2(2)	-	-	2(2)	-	-	1%	
Leukocyte elastase inhibitor C, partial	A	A	A	43705	160	111	74	4(4)	3(3)	1(1)	4(4)	3(3)	1(1)	13%	9%	2%	KRY55578.1
	B	B	B		128	164	131	2(2)	5(5)	6(4)	2(2)	4(4)	5(4)	6%	14%	13%	
LIM domain and actin-binding protein 1	A	-	-	108499	66	-	-	1(1)	-	-	1(1)	-	-	1%	-	-	KRY61178.1
Lipase maturation factor 2	C	C	C	77441	81	157	136	1(1)	3(3)	2(2)	1(1)	3(3)	1(1)	2%	7%	1%	KRY59894.1
	D	-	-		111	-	-	1(1)	-	-	1(1)	-	-	3%	-	-	
	-	-	E		-	-	72	-	-	1(1)	-	-	1(1)	-	-	1%	
	-	-	G		-	-	97	-	-	2(2)	-	-	2(2)	-	-	4%	
Lipopolysaccharide biosynthesis protein RfbH	-	-	B	50863	-	-	64	-	-	1(1)	-	-	1(1)	-	-	1%	KRY52541.1
Lissencephaly-1 -like protein	A	A	A	62181	61	65	75	1(1)	1(1)	1(1)	1(1)	1(1)	1(1)	1%	1%	2%	KRY60128.1
L-lactate dehydrogenase	-	C	-	44144	-	89	-	-	1(1)	-	-	1(1)	-	-	3%	-	KRY58174.1
Lon protease -like protein, mitochondrial, partial	D	D	-	107329	209	80	-	7(7)	2(2)	-	7(7)	2(2)	-	8%	1%	-	KRY50200.1
Lupus La -like protein A, partial	B	B	B	120320	74	75	105	1(1)	2(2)	1(1)	1(1)	2(2)	1(1)	1%	2%	1%	KRY45724.1
	C	C	C		269	204	262	5(5)	4(4)	4(4)	5(5)	4(4)	2(2)	6%	5%	3%	
Lysine--tRNA ligase	-	C	C	68729	-	123	111	-	2(2)	3(3)	-	2(2)	3(3)	-	4%	4%	KRY55658.1

Lysosomal aspartic protease, partial	A	A	A	46676	261	215	179	3(3)	3(3)	2(2)	3(3)	3(3)	2(2)	10%	13%	6%	KRY49432.1
	-	B	B		-	108	128	-	1(1)	1(1)	-	1(1)	1(1)	-	6%	6%	
Malate dehydrogenase, cytoplasmic	A	A	A	36616	143	154	106	2(2)	2(2)	1(1)	2(2)	2(2)	1(1)	8%	8%	5%	KRY57329.1
	B	B	-		109	129	-	2(2)	2(2)	-	2(2)	2(2)	-	6%	8%	-	
	C	C	C		143	84	156	4(4)	3(3)	3(3)	4(4)	3(3)	3(3)	15%	7%	10%	
	D	D	D		97	192	115	3(2)	5(5)	2(2)	3(2)	5(5)	2(2)	6%	15%	8%	
	-	-	E		-	-	183	-	-	5(5)	-	-	4(4)	-	-	15%	
	-	-	F		-	-	141	-	-	3(3)	-	-	3(3)	-	-	10%	
	-	-	G		-	-	125	-	-	2(2)	-	-	2(2)	-	-	6%	
Malate dehydrogenase, partial	-	-	A	43114	-	-	63	-	-	1(1)	-	-	1(1)	-	-	2%	KRY47491.1
Mannose-6-phosphate isomerase, partial	A	A	A	47870	329	181	236	9(9)	6(6)	7(7)	7(7)	4(4)	5(5)	23%	14%	15%	KRY55073.1
Mediator of RNA polymerase II transcription subunit 23	B	B	B	258401	78	145	151	1(1)	3(3)	2(2)	1(1)	3(3)	2(2)	0%	1%	1%	KRY54660.1
	-	C	-		-	100	-	-	1(1)	-	-	1(1)	-	-	0%	-	
Medium-chain specific acyl-CoA dehydrogenase, mitochondrial, partial	A	A	A	46251	167	136	209	3(3)	4(4)	6(5)	3(3)	4(4)	65(5)	8%	10%	12%	KRY52707.1
Mesocentin	-	-	E	363972	-	-	88	-	-	1(1)	-	-	1(1)	-	-	0%	KRY60969.1
	-	-	G		-	-	154	-	-	2(2)	-	-	2(2)	-	-	0%	
Methionine aminopeptidase 1, partial	A	A	A	45052	107	137	134	2(2)	5(4)	3(3)	2(2)	5(4)	3(3)	5%	13%	8%	KRY48197.1
Methionine--tRNA ligase, cytoplasmic, partial	D	-	-	102963	99	-	-	1(1)	-	-	1(1)	-	-	1%	-	-	KRY51150.1
Methylmalonyl-CoA mutase, mitochondrial	C	C	C	96758	232	246	237	5(5)	6(6)	4(4)	5(5)	6(6)	4(4)	8%	8%	6%	KRY55816.1
Mitochondrial chaperone BCS1	A	-	-	168033	103	-	-	1(1)	-	-	1(1)	-	-	1%	-	-	KRY58920.1
Mitochondrial EF-Tu1 precursor	B	B	B	53698	88	232	226	2(2)	7(7)	7(6)	2(2)	7(7)	7(6)	4%	15%	17%	BAF32575.1
	-	-	E		-	-	62	-	-	1(1)	-	-	1(1)	-	-	2%	
Mitochondrial inner membrane protein	C	C	C	51259	139	123	101	4(3)	2(2)	1(1)	4(3)	2(2)	1(1)	9%	5%	3%	KRY52343.1
Mitochondrial ornithine transporter 1	B	B	B	131282	107	126	120	2(2)	3(3)	2(2)	2(2)	3(3)	2(2)	2%	3%	2%	KRY52596.1
Mitochondrial succinyl-CoA ligase subunit beta -like protein	A	A	A	47920	233	196	249	7(7)	6(6)	7(7)	7(7)	6(6)	7(7)	20%	15%	20%	KRY60682.1
	-	B	-		-	96	-	-	1(1)	-	-	1(1)	-	-	2%	-	
	-	C	C		-	70	65	-	2(1)	1(1)	-	2(1)	1(1)	-	4%	2%	
Mitochondrial-processing peptidase subunit alpha	A	A	A	114673	150	145	163	4(3)	4(3)	4(4)	4(3)	4(3)	4(4)	4%	4%	4%	KRY50357.1
Mitochondrial-processing peptidase subunit beta	A	A	A	95488	118	73	96	4(4)	2(2)	2(2)	4(4)	2(2)	2(2)	5%	2%	2%	KRY53080.1
	B	B	B		281	414	519	8(8)	11(10)	14(14)	7(7)	10(9)	11(11)	11%	15%	16%	
Molybdenum cofactor synthesis protein cinnamon	-	C	C	71831	-	99	77	-	2(2)	1(1)	-	2(2)	1(1)	-	3%	1%	KRY53927.1
Motochondrial isocitrate dehydrogenase, partial	A	A	A	91260	147	93	129	4(4)	1(1)	2(2)	4(4)	1(1)	2(2)	6%	1%	3%	KRY60163.1
mRNA-capping enzyme	C	-	-	63559	66	-	-	1(1)	-	-	1(1)	-	-	2%	-	-	KRY57625.1
Multiple epidermal growth factor-like domains protein 11	-	-	E	126169	-	-	77	-	-	2(2)	-	-	2(2)	-	-	2%	KRY57673.1
Muscle M-line assembly protein unc-89	-	-	E	822834	-	-	82	-	-	2(2)	-	-	2(2)	-	-	0%	KRY53785.1
	-	-	G		-	-	70	-	-	1(1)	-	-	1(1)	-	-	0%	
Myophilin	-	A	A	29149	-	94	91	-	1(1)	1(1)	-	1(1)	1(1)	-	4%	4%	KRY53866.1
	-	B	B		-	101	98	-	1(1)	1(1)	-	1(1)	1(1)	-	4%	4%	
	C	C	C		95	106	78	1(1)	1(1)	1(1)	1(1)	1(1)	1(1)	4%	4%	5%	
	D	-	-		137	-	-	2(2)	-	-	2(2)	-	-	11%	-	-	
	-	-	E		-	-	74	-	-	1(1)	-	-	1(1)	-	-	5%	
	-	-	F		-	-	96	-	-	1(1)	-	-	1(1)	-	-	4%	
Myosin heavy chain, non-muscle	-	-	G	234900	-	-	82	-	-	2(2)	-	-	2(2)	-	-	0%	KRY59395.1
Myosin regulatory light chain 1, partial	-	C	-	21489	-	98	-	-	1(1)	-	-	1(1)	-	-	7%	-	KRY55173.1
	D	D	-		100	97	-	1(1)	1(1)	-	1(1)	1(1)	-	7%	7%	-	
	-	-	G		-	-	76	-	-	2(2)	-	-	2(2)	-	-	13%	
Myosin-4, partial	A	A	A	233904	172	172	116	6(6)	4(4)	2(2)	6(6)	4(4)	2(2)	4%	2%	1%	KRY50587.1
	-	B	B		-	75	87	-	1(1)	1(1)	-	1(1)	1(1)	-	0%	0%	

	C	C	C		646	848	407	18(17)	21(19)	12(11)	16(15)	18(16)	9(8)	11%	12%	5%	
	D	D	D		1145	709	312	33(30)	20(19)	12(12)	30(27)	19(18)	12(12)	18%	11%	7%	
	-	-	E		-	-	1645	-	-	60(59)	-	-	47(46)	-	-	26%	
	-	-	F		-	-	1611	-	-	54(53)	-	-	44(43)	-	-	24%	
	-	-	G		-	-	1514	-	-	55(54)	-	-	45(44)	-	-	23%	
N-acetyltransferase 10	C	C	-	237253	64	79	-	1(1)	1(1)	-	1(1)	1(1)	-	0%	0%	-	KRY54548.1
NAD kinase 2, mitochondrial, partial	-	B	B	53835	-	113	148	-	2(2)	2(2)	-	2(2)	2(2)	-	6%	6%	KRY54079.1
NAD(P) transhydrogenase, mitochondrial	D	D	D	114749	201	210	73	4(4)	4(4)	1(1)	4(4)	4(4)	1(1)	4%	4%	1%	KRY57619.1
	-	-	E		-	-	115	-	-	2(2)	-	-	2(2)	-	-	2%	
	-	-	G		-	-	89	-	-	1(1)	-	-	1(1)	-	-	1%	
NAD-dependent alcohol dehydrogenase	-	D	-	28802	-	61	-	-	1(1)	-	-	1(1)	-	-	4%	-	KRY47388.1
NADP-dependent malic enzyme, mitochondrial, partial	B	B	B	136914	79	100	118	2(2)	3(3)	5(4)	2(2)	3(3)	5(4)	1%	2%	4%	KRY59664.1
	C	C	C		1956	1170	1184	46(45)	31(31)	31(31)	26(25)	22(22)	24(24)	26%	23%	27%	
	D	D	D		306	108	93	10(9)	3(2)	1(1)	10(9)	3(2)	1(1)	11%	2%	0%	
	-	-	E		-	-	214	-	-	7(7)	-	-	6(6)	-	-	5%	
	-	-	F		-	-	137	-	-	3(3)	-	-	3(3)	-	-	2%	
	-	-	G		-	-	142	-	-	3(3)	-	-	3(3)	-	-	2%	
NADP-dependent malic enzyme, partial	C	C	-	67015	160	154	-	6(6)	5(4)	-	6(6)	5(4)	-	12%	6%	-	KRY50082.1
NADPH-dependent diflavin oxidoreductase 1	C	C	-	68945	91	73	-	1(1)	1(1)	-	1(1)	1(1)	-	2%	2%	-	KRY52027.1
Nedd8-activating enzyme E1 regulatory subunit	-	-	B	81417	-	-	72	-	-	1(1)	-	-	1(1)	-	-	1%	KRY49521.1
	-	-	C		-	-	154	-	-	2(2)	-	-	2(2)	-	-	4%	
	D	-	-		82	-	-	1(1)	-	-	1(1)	-	-	1%	-	-	
Neurogenic locus notch -like protein	-	D	-	478274	-	61	-	-	1(1)	-	-	1(1)	-	-	0%	-	KRY46373.1
Neuroglian	C	C	C	151761	221	229	217	4(4)	5(5)	3(3)	4(4)	5(5)	3(3)	3%	5%	4%	KRY57340.11 51761
	D	-	D		85	-	76	1(1)	-	1(1)	1(1)	-	1(1)	0%	-	0%	
	-	-	E		-	-	274	-	-	7(7)	-	-	7(7)	-	-	6%	
	-	-	F		-	-	76	-	-	1(1)	-	-	1(1)	-	-	0%	
Neutral alpha-glucosidase AB	D	-	-	132754	148	-	-	3(3)	-	-	3(3)	-	-	4%	-	-	KRY60908.1
	-	-	E		-	-	84	-	-	1(1)	-	-	1(1)	-	-	0%	
Nicalin-1	C	C	C	67341	60	60	66	1(1)	1(1)	1(1)	1(1)	1(1)	1(1)	1%	1%	1%	KRY55697.1
Nicastrin, partial	D	-	D	84040	117	-	82	3(3)	-	1(1)	3(3)	-	1(1)	5%	-	1%	KRY58667.1
Nicotinamide phosphoribosyltransferase, partial	-	B	-	54479	-	75	-	-	1(1)	-	-	1(1)	-	-	2%	-	KRY61111.1
Nidogen-1	-	-	E	194996	-	-	91	-	-	2(2)	-	-	2(2)	-	-	1%	KRY48612.1
	-	-	F		-	-	91	-	-	1(1)	-	-	1(1)	-	-	0%	
Nodal modulator 1	-	-	E	254703	-	-	62	-	-	1(1)	-	-	1(1)	-	-	0%	KRY49785.1
Non-POU domain-containing octamer-binding protein, partial	-	B	-	51272	-	65	-	-	1(1)	-	-	1(1)	-	-	2%	-	KRY59182.1
NSFL1 cofactor p47	-	A	A	96155	-	64	76	-	1(1)	1(1)	-	1(1)	1(1)	-	1%	1%	KRY55488.1
	-	-	B		-	-	61	-	-	1(0)	-	-	1(0)	-	-	1%	
Nuclear export mediator factor Nemf	B	-	B	96804	91	-	78	1(1)	-	2(1)	1(1)	-	2(1)	1%	-	2%	KRY60186.1
Nuclease domain-containing protein	C	C	C	105353	63	90	68	1(1)	1(1)	2(2)	1(1)	1(1)	2(2)	0%	1%	2%	KRY53536.1
	D	D	D		409	363	127	11(11)	10(10)	3(3)	10(10)	10(10)	3(3)	12%	12%	3%	
	-	-	E		-	-	163	-	-	5(5)	-	-	5(5)	-	-	5%	
	-	-	G		-	-	108	-	-	2(2)	-	-	2(2)	-	-	2%	
Nucleolar GTP-binding protein 1	-	A	-	217720	-	80	-	-	1(1)	-	-	1(1)	-	-	0%	-	KRY59079.1
Nucleosome assembly protein 1-like 4	-	-	A	42769	-	-	67	-	-	1(1)	-	-	1(1)	-	-	2%	KRY56244.1
	B	B	B		111	171	205	4(4)	4(4)	5(5)	4(4)	4(4)	5(5)	12%	19%	21%	
NudC domain-containing protein 1	C	C	-	70408	203	115	-	5(5)	4(4)	-	5(5)	4(4)	-	8%	4%	-	KRY59809.1
Obg-like ATPase 1	A	A	A	46859	257	129	300	9(9)	3(2)	9(9)	9(9)	3(2)	9(9)	24%	7%	25%	KRY54762.1

	-	B	-		-	63	-	-	1(1)	-	-	1(1)	-	-	2%	-	
Oligosaccharyltransferase complex subunit OSTC	A	A	-	35534	98	78	-	1(1)	1(1)	-	1(1)	1(1)	-	4%	4%	-	KRY48322.1
	-	B	B		-	66	105	-	1(1)	1(1)	-	1(1)	1(1)	-	4%	4%	
	C	C	C		97	87	91	1(1)	1(1)	1(1)	1(1)	1(1)	1(1)	4%	4%	4%	
	D	D	-		111	86	-	1(1)	1(1)	-	1(1)	1(1)	-	4%	4%	-	
Ornithine decarboxylase, partial	A	A	A	146144	161	142	158	3(3)	2(2)	3(3)	3(3)	2(2)	3(3)	4%	2%	3%	KRY52484.1
Oxysterol-binding protein-related protein 9	D	-	-	92864	94	-	-	2(2)	-	-	2(2)	-	-	2%	-	-	KRY56357.1
Papilin, partial	A	A	A	248330	65	137	83	1(1)	2(2)	1(1)	1(1)	2(2)	1(1)	0%	1%	0%	KRY57082.1
	-	D	-	250404	-	102	-	-	1(1)	-	-	1(1)	-	-	0%	-	KRY54791.1
	-	-	E		-	-	65	-	-	1(1)	-	-	1(1)	-	-	0%	
	-	-	G		-	-	89	-	-	3(3)	-	-	3(3)	-	-	1%	
Paramyosin, partial	C	C	C	96956	193	227	149	4(4)	5(5)	4(4)	4(4)	5(5)	4(4)	6%	7%	5%	KRY49321.1
	-	D	D		-	1580	603	-	51(49)	19(19)	-	35(34)	17(17)	-	43%	24%	
	-	-	E		-	-	377	-	-	13(13)	-	-	13(13)	-	-	17%	
	-	-	F		-	-	209	-	-	6(6)	-	-	6(6)	-	-	7%	
	-	-	G		-	-	278	-	-	10(10)	-	-	9(9)	-	-	12%	
PDZ and LIM domain protein Zasp, partial	-	-	B	87739	-	-	61	-	-	1(1)	-	-	1(1)	-	-	1%	KRY58370.1
Pecanex-like protein 1	D	D	-	364322	131	66	-	5(4)	1(1)	-	5(4)	1(1)	-	1%	0%	-	KRY53192.1
Peptidyl-glycine alpha-amidating monooxygenase A	D	D	-	47555	120	73	-	1(1)	1(1)	-	1(1)	1(1)	-	4%	4%	-	KRY57729.1
Peroxiredoxin-2, partial	A	A	A	23367	317	251	260	6(6)	4(4)	5(5)	6(6)	4(4)	5(5)	39%	29%	29%	KRY49990.1
	-	-	B		-	-	63	-	-	1(1)	-	-	1(1)	-	-	5%	
	C	C	C		110	98	93	1(1)	2(2)	1(1)	1(1)	2(2)	1(1)	5%	10%	8%	
	-	-	E		-	-	95	-	-	1(1)	-	-	1(1)	-	-	5%	
Phenylalanine--tRNA ligase beta subunit	C	C	C	70907	159	241	144	4(4)	8(8)	4(4)	4(4)	8(8)	4(4)	6%	14%	6%	KRY59452.1
Phosphate carrier protein, mitochondrial, partial	A	-	-	69926	90	-	-	1(1)	-	-	1(1)	-	-	2%	-	-	KRY57035.1
	-	B	B		-	138	157	-	2(2)	3(3)	-	2(2)	3(3)	-	4%	6%	
Phosphoacetylglucosamine mutase	C	-	-	76610	76	-	-	1(1)	-	-	1(1)	-	-	1%	-	-	KRY55926.1
	-	-	E		-	-	86	-	-	1(1)	-	-	1(1)	-	-	1%	
Phosphoglucomutase-1	B	-	B	62761	68	-	64	1(1)	-	1(1)	1(1)	-	1(1)	1%	-	2%	KRY58984.1
	C	C	C		371	195	167	10(10)	5(5)	6(6)	9(9)	5(5)	6(6)	18%	11%	10%	
	D	D	-		81	70	-	1(1)	1(1)	-	1(1)	1(1)	-	1%	1%	-	
Phosphoinositide 3-kinase regulatory subunit 4, partial	-	A	A	249279	-	208	160	-	5(5)	3(3)	-	5(5)	3(3)	-	2%	1%	KRY53638.1
	-	C	C		-	83	94	-	1(1)	1(1)	-	1(1)	1(1)	-	0%	0%	
	D	D	-		81	116	-	1(1)	2(2)	-	1(1)	2(2)	-	0%	1%	-	
	-	-	E		-	-	73	-	-	1(1)	-	-	1(1)	-	-	0%	
Phospholipase A-2-activating protein	-	-	G	90526	-	-	70	-	-	1(1)	-	-	1(1)	-	-	1%	KRY60109.1
Phosphoribosyl pyrophosphate synthase-associated protein 2, partial	-	B	B	89366	-	139	130	-	4(4)	4(4)	-	3(3)	4(4)	-	4%	6%	KRY59095.1
Phosphorylase b kinase gamma catalytic chain, skeletal muscle/heart isoform	-	-	A	47526	-	-	66	-	-	1(1)	-	-	1(1)	-	-	1%	KRY59142.1
Plasminogen	-	C	-	84221	-	67	-	-	1(1)	-	-	1(1)	-	-	1%	-	KRY53811.1
Plexin domain-containing protein 2	D	-	-	56442	68	-	-	2(2)	-	-	2(2)	-	-	4%	-	-	KRY53501.1
pliceosome RNA helicase DDX39B	-	B	-	83161	-	175	-	-	6(6)	-	-	6(6)	-	-	8%	-	KRY50703.1
Poly(U)-specific endoribonuclease -like protein, partial	A	-	A	61077	73	-	60	2(2)	-	1(1)	2(2)	-	1(1)	3%	-	2%	KRY51160.1
Polyadenylate-binding protein	-	A	A	63357	-	120	97	-	2(2)	2(2)	-	2(2)	2(2)	-	5%	3%	KRY49582.1
	B	B	B		68	89	143	1(1)	1(1)	3(3)	1(1)	1(1)	3(3)	2%	3%	7%	
	-	C	C		-	461	504	-	13(13)	16(16)	-	13(13)	15(15)	-	28%	28%	
Polyadenylate-binding protein 1, partial	A	-	-	90660	187	-	-	4(4)	-	-	4(4)	-	-	6%	-	-	KRY49578.1
	C	-	-		364	-	-	11(11)	-	-	11(11)	-	-	18%	-	-	

Poly-cysteine and histidine-tailed protein, partial	D	D	-	53251	68	76	-	1(1)	1(1)	-	1(1)	1(1)	-	1%	1%	-	KRY58369.1
	-	-	E		-	-	76	-	-	1(1)	-	-	1(1)	-	-	1%	
	-	-	G		-	-	64	-	-	1(1)	-	-	1(1)	-	-	1%	
Polyribonucleotide nucleotidyltransferase 1, mitochondrial	C	C	-	137745	103	148	-	2(1)	3(3)	-	2(1)	3(3)	-	2%	2%	-	KRY55114.1
	-	D	-		-	69	-	-	1(1)	-	-	1(1)	-	-	1%	-	
Polyubiquitin	A	A	A	71793	99	103	103	1(1)	1(1)	1(1)	1(1)	1(1)	1(1)	2%	2%	2%	KRY46475.1
	B	B	B		84	124	72	2(2)	2(1)	1(1)	2(2)	2(1)	1(1)	3%	3%	2%	
	-	C	C		-	60	135	-	1(0)	2(2)	-	1(0)	2(2)	-	1%	2%	
	-	D	D		-	121	70	-	1(1)	1(1)	-	1(1)	1(1)	-	2%	2%	
	-	-	E		-	-	106	-	-	1(1)	-	-	1(1)	-	-	2%	
	-	-	F		-	-	114	-	-	1(1)	-	-	1(1)	-	-	2%	
	-	-	G		-	-	110	-	-	1(1)	-	-	1(1)	-	-	2%	
Polyubiquitin -like protein, partial	-	-	A	12545	-	-	103	-	-	1(1)	-	-	1(1)	-	-	14%	KRY37210.1
	B	B	B		84	124	72	2(2)	2(1)	1(1)	2(2)	2(1)	1(1)	22%	22%	14%	
	-	C	C		-	60	135	-	1(0)	2(2)	-	1(0)	2(2)	-	8%	14%	
	-	D	D		-	121	70	-	1(1)	1(1)	-	1(1)	1(1)	-	14%	14%	
	-	-	E		-	-	106	-	-	1(1)	-	-	1(1)	-	-	14%	
	-	-	F		-	-	114	-	-	1(1)	-	-	1(1)	-	-	14%	
	-	-	G		-	-	110	-	-	1(1)	-	-	1(1)	-	-	14%	
Polyubiquitin-B, partial	A	A	A	17394	99	103	103	1(1)	1(1)	1(1)	1(1)	1(1)	1(1)	10%	10%	10%	KRY44570.1
	B	B	B		84	124	72	2(2)	2(1)	1(1)	2(2)	2(1)	1(1)	16%	16%	10%	
	-	C	C		-	60	135	-	1(0)	2(2)	-	1(0)	2(2)	-	5%	11%	
	-	D	D		-	121	70	-	1(1)	1(1)	-	1(1)	1(1)	-	10%	10%	
	-	-	E		-	-	106	-	-	1(1)	-	-	1(1)	-	-	10%	
	-	-	F		-	-	114	-	-	1(1)	-	-	1(1)	-	-	10%	
	-	-	G		-	-	110	-	-	1(1)	-	-	1(1)	-	-	10%	
Polyubiquitin-C	A	A	A	39424	99	103	103	1(1)	1(1)	1(1)	1(1)	1(1)	1(1)	4%	4%	4%	KRY55776.1
	B	B	B		84	124	72	2(2)	2(1)	1(1)	2(2)	2(1)	1(1)	7%	7%	4%	
	-	C	-		-	60	-	-	1(0)	-	-	1(0)	-	-	2%	-	
	-	D	D		-	121	70	-	1(1)	1(1)	-	1(1)	1(1)	-	4%	4%	
	-	-	E		-	-	106	-	-	1(1)	-	-	1(1)	-	-	4%	
	-	-	F		-	-	114	-	-	1(1)	-	-	1(1)	-	-	4%	
	-	-	G		-	-	110	-	-	1(1)	-	-	1(1)	-	-	4%	
Polyubiquitin-C, partial	-	-	C	75000	-	-	135	-	-	2(2)	-	-	2(2)	-	-	2%	KRY45530.1
PRA1 family protein 2	-	-	G	21421	-	-	92	-	-	1(1)	-	-	1(1)	-	-	5%	KRY51440.1
Proliferation-associated protein 2G4, partial	A	A	A	49607	135	168	139	4(4)	4(4)	5(4)	4(4)	4(4)	5(4)	9%	11%	12%	KRY53054.1
	B	B	B		196	327	321	6(6)	11(11)	10(10)	6(6)	11(11)	9(9)	14%	27%	26%	
Prolyl 4-hydroxylase subunit alpha-2	C	C	C	130247	89	82	82	2(2)	2(2)	1(1)	2(2)	2(2)	1(1)	2%	2%	1%	KRY58929.1
Propionyl-CoA carboxylase alpha chain, mitochondrial	A	-	-	441396	66	-	-	1(1)	-	-	1(1)	-	-	0%	-	-	KRY51819.1
	B	B	B		67	128	93	1(1)	2(2)	1(1)	1(1)	2(2)	1(1)	0%	0%	0%	
	C	-	C		241	-	220	4(4)	-	5(5)	4(4)	-	5(5)	1%	-	1%	
	-	D	D		-	187	121	-	5(5)	1(1)	-	5(5)	1(1)	-	1%	0%	
	-	-	F		-	-	151	-	-	3(3)	-	-	3(3)	-	-	0%	
Propionyl-CoA carboxylase beta chain, mitochondrial, partial	A	A	A	59995	142	113	152	4(4)	2(2)	3(3)	4(4)	2(2)	3(3)	9%	4%	7%	KRY52668.1
	B	B	B		273	417	281	8(8)	11(11)	10(9)	8(8)	9(9)	10(9)	18%	22%	21%	
	C	C	C		91	86	171	2(2)	2(2)	5(5)	2(2)	2(2)	5(5)	4%	4%	11%	
	D	D	D		87	200	123	3(3)	5(5)	3(3)	3(3)	5(5)	3(3)	6%	11%	7%	
	-	-	E		-	-	140	-	-	5(5)	-	-	5(5)	-	-	10%	
	-	-	F		-	-	125	-	-	3(3)	-	-	3(3)	-	-	6%	

	-	-	G		-	-	130	-	-	2(2)	-	-	2(2)	-	-	5%	
Proteasome subunit alpha type-3, partial	-	-	C	77206	-	-	81	-	-	1(1)	-	-	1(1)	-	-	2%	KRY47615.1
Proteasome subunit alpha type-6	-	-	C	27985	-	-	99	-	-	1(1)	-	-	1(1)	-	-	5%	KRY50620.1
Protein AAR2 -like protein	-	A	A	68097	-	80	80	-	1(1)	1(1)	-	1(1)	1(1)	-	1%	1%	KRY56554.1
Protein arginine N-methyltransferase 1, partial	A	A	A	41784	144	230	308	4(4)	8(8)	8(8)	4(4)	8(8)	8(8)	10%	23%	24%	KRY51927.1
Protein arginine N-methyltransferase 6, partial	-	A	-	77995	-	64	-	-	1(1)	-	-	1(1)	-	-	1%	-	KRY58901.1
Protein argonaute-2	-	C	C	239895	-	60	62	-	1(0)	1(1)	-	1(0)	1(1)	-	0%	0%	KRY49018.1
	-	-	E		-	-	78	-	-	1(1)	-	-	1(1)	-	-	0%	
	-	-	F		-	-	71	-	-	1(1)	-	-	1(1)	-	-	0%	
	-	-	G		-	-	78	-	-	1(1)	-	-	1(1)	-	-	0%	
Protein argonaute-2, partial	D	D	-	106796	281	237	-	9(9)	6(6)	-	9(9)	6(6)	-	14%	9%	-	KRY60812.1
Protein CLEC16A, partial	C	-	C	246145	211	-	133	5(5)	-	2(2)	5(5)	-	2(2)	3%	-	1%	KRY50389.1
Protein DDI1 -like protein 2	-	B	B	44709	-	62	109	-	1(1)	3(3)	-	1(1)	3(3)	-	5%	10%	KRY49840.1
Protein disulfide-isomerase A6	A	A	-	59296	86	121	-	1(1)	3(3)	-	1(1)	3(3)	-	1%	7%	-	KRY54523.1
	C	-	C		84	-	87	1(1)	-	1(1)	1(1)	-	1(1)	1%	-	1%	
	D	-	-		105	-	-	3(3)	-	-	3(3)	-	-	6%	-	-	
	-	-	F		-	-	94	-	-	2(2)	-	-	2(2)	-	-	4%	
Protein disulfide-isomerase, partial	C	C	C	84095	69	116	131	1(1)	3(3)	2(2)	1(1)	3(3)	2(2)	1%	4%	3%	KRY60941.1
Protein ERGIC-53, partial	B	B	B	57042	70	71	79	2(2)	1(1)	1(1)	2(2)	1(1)	1(1)	3%	1%	1%	KRY52865.1
Protein HGV2	-	C	-	48344	-	97	-	-	1(1)	-	-	1(1)	-	-	2%	-	KRY60606.1
Protein HIRA	-	C	C	155136	-	211	206	-	5(5)	5(5)	-	5(5)	5(5)	-	4%	4%	KRY45698.1
Protein lap1	C	-	-	162713	65	-	-	1(1)	-	-	1(1)	-	-	0%	-	-	KRY57372.1
Protein LSM14 -like protein A	C	C	C	51261	135	82	82	2(2)	2(2)	1(1)	2(2)	2(2)	1(1)	5%	5%	2%	KRY47653.1
Protein MON2 -like protein	-	B	B	228385	-	64	61	-	1(1)	1(1)	-	1(1)	1(1)	-	0%	0%	KRY53690.1
Protein pad-1, partial	A	-	A	269147	72	-	81	1(1)	-	1(1)	1(1)	-	1(1)	0%	-	0%	KRY55730.1
Protein phosphatase 1E	-	-	B	48913	-	-	87	-	-	1(1)	-	-	1(1)	-	-	4%	KRY55596.1
Protein phosphatase methylesterase 1	-	-	A	70545	-	-	67	-	-	1(1)	-	-	1(1)	-	-	1%	KRY57191.1
Protein Skeletor, isoforms B/C	C	C	C	158475	162	206	156	3(3)	5(5)	3(3)	3(3)	5(5)	3(3)	2%	4%	3%	KRY58447.1
Protein Skeletor, isoforms D/E	C	C	-	166013	162	206	-	3(3)	5(5)	-	3(3)	5(5)	-	2%	4%	-	KRY58448.1
Protein transport protein Sec24C	D	D	D	131036	169	155	137	4(4)	4(4)	3(3)	4(4)	4(4)	3(3)	3%	4%	2%	KRY54357.1
	-	-	E		-	-	172	-	-	4(4)	-	-	4(4)	-	-	4%	
Protein transport protein Sec61 subunit alpha isoform 2	-	A	-	46439	-	64	-	-	1(1)	-	-	1(1)	-	-	2%	-	KRY54101.1
Protein UBASH3A -like protein	-	-	C	142437	-	-	99	-	-	1(1)	-	-	1(1)	-	-	1%	KRY56821.1
	-	-	E		-	-	104	-	-	1(1)	-	-	1(1)	-	-	1%	
Protein unc-45 -like protein A	D	-	-	398201	127	-	-	1(1)	-	-	1(1)	-	-	0%	-	-	KRY58313.1
Proton myo-inositol cotransporter, partial	C	-	-	71203	86	-	-	2(2)	-	-	2(2)	-	-	3%	-	-	KRY54302.1
Pseudouridine-5'-monophosphatase	-	-	A	70411	-	-	66	-	-	1(1)	-	-	1(1)	-	-	1%	KRY55631.1
Puromycin-sensitive aminopeptidase, partial	D	D	-	101232	94	87	-	3(3)	3(2)	-	3(3)	3(2)	-	4%	2%	-	KRY58185.1
	-	-	E		-	-	76	-	-	1(1)	-	-	1(1)	-	-	1%	
putative [pyruvate dehydrogenase (acetyl-transferring)] kinase, mitochondrial, partial	A	A	A	53118	181	111	157	4(4)	3(2)	3(3)	3(3)	3(2)	3(3)	8%	8%	8%	KRY57184.1
putative 26S proteasome regulatory subunit rpn-6.1	B	-	B	51807	94	-	96	1(1)	-	1(1)	1(1)	-	1(1)	2%	-	2%	KRY56382.1
putative aconitate hydratase, mitochondrial	A	A	A	232635	96	87	88	1(1)	1(1)	1(1)	1(1)	1(1)	1(1)	0%	0%	0%	KRY56796.1
	-	B	-		-	108	-	-	3(2)	-	-	3(2)	-	-	1%	-	
	C	C	-		97	80	-	2(2)	2(2)	-	2(2)	2(2)	-	1%	1%	-	
	D	D	D		206	93	95	5(5)	1(1)	2(2)	5(5)	1(1)	2(2)	3%	0%	1%	
	-	-	E		-	-	151	-	-	3(3)	-	-	3(3)	-	-	1%	
	-	-	F		-	-	67	-	-	1(1)	-	-	1(1)	-	-	0%	
	-	-	G		-	-	141	-	-	3(3)	-	-	3(3)	-	-	2%	

putative aminopeptidase W07G4.4	C	C	C	66737	660	331	420	16(16)	9(9)	11(11)	14(14)	8(8)	11(11)	32%	17%	23%	KRY54333.1
putative arginine kinase F46H5.3	-	A	A	45915	-	403	503	-	12(12)	12(12)	-	10(10)	10(10)	-	32%	33%	KRY58392.1
	-	B	B		-	110	146	-	1(1)	2(2)	-	1(1)	2(2)	-	3%	7%	
	C	C	-		158	90	-	3(3)	3(3)	-	3(3)	3(3)	-	10%	9%	-	
	D	D	D		135	136	75	3(3)	3(3)	1(1)	3(3)	3(3)	1(1)	9%	9%	3%	
	-	-	E		-	-	138	-	-	3(3)	-	-	3(3)	-	-	10%	
	-	-	F		-	-	94	-	-	2(2)	-	-	2(2)	-	-	6%	
	-	-	G		-	-	89	-	-	4(2)	-	-	4(2)	-	-	11%	
putative aspartate aminotransferase, cytoplasmic	A	-	A	205821	81	-	70	1(1)	-	1(1)	1(1)	-	1(1)	1%	-	1%	KRY48097.1
putative delta-1-pyrroline-5-carboxylate synthase, partial	D	-	-	86241	62	-	-	1(1)	-	-	1(1)	-	-	1%	-	-	KRY49148.1
putative glutamine--tRNA ligase, partial	C	C	C	207108	164	164	91	3(3)	6(6)	3(3)	3(3)	6(6)	3(3)	2%	3%	1%	KRY57355.1
putative glycogen [starch] synthase	D	-	-	79600	94	-	-	3(3)	-	-	3(3)	-	-	4%	-	-	KRY59075.1
putative histone-binding protein Caf1	B	B	B	50720	106	75	101	3(3)	2(2)	2(2)	3(3)	2(2)	2(2)	6%	4%	5%	KRY53239.1
putative integrin beta-like protein	A	A	A	59305	259	122	169	4(4)	1(1)	4(4)	4(4)	1(1)	4(4)	13%	4%	11%	KRY46033.1
	-	B	B		-	207	222	-	3(3)	6(6)	-	3(3)	6(6)	-	9%	20%	
putative mannosyl-oligosaccharide glucosidase	D	D	D	95127	273	158	105	7(7)	4(4)	3(3)	7(7)	4(4)	3(3)	11%	6%	4%	KRY53241.1
putative oxidoreductase -like protein	A	A	A	47000	78	76	81	1(1)	1(1)	1(1)	1(1)	1(1)	1(1)	2%	2%	2%	KRY46863.1
putative peptidyl-glycine alpha-amidating monooxygenase T19B4.1	D	-	-	46826	120	-	-	1(1)	-	-	1(1)	-	-	4%	-	-	KRY57727.1
putative phosphoglycerate kinase	A	A	-	62385	999	699	-	28(27)	25(23)	-	19(18)	18(17)	-	45%	41%	-	KRY46312.1
	-	-	B		-	-	171	-	-	5(5)	-	-	5(5)	-	-	12%	
	C	-	-		135	-	-	2(2)	-	-	2(2)	-	-	4%	-	-	
	B	B	-	62151	80	75	-	2(2)	1(1)	-	2(2)	1(1)	-	4%	1%	-	KRY46311.1
	-	-	C		-	-	136	-	-	2(2)	-	-	2(2)	-	-	4%	
	D	D	-		134	107	-	3(3)	3(3)	-	3(3)	3(3)	-	8%	7%	-	
	-	-	E		-	-	119	-	-	2(2)	-	-	2(2)	-	-	4%	
	-	-	F		-	-	73	-	-	1(1)	-	-	1(1)	-	-	1%	
putative phospholipase B-like 2, partial	A	A	A	65395	81	66	63	2(2)	1(1)	1(1)	2(2)	1(1)	1(1)	3%	1%	1%	KRY61331.1
putative pterin-4-alpha-carbinolamine dehydratase	C	C	-	130533	85	73	-	2(2)	1(1)	-	2(2)	1(1)	-	1%	1%	-	KRY58441.1
putative pyruvate dehydrogenase E1 component subunit alpha, mitochondrial	A	A	A	49940	172	161	267	5(5)	4(4)	6(6)	5(5)	4(4)	6(6)	15%	10%	18%	KRY58181.1
	C	C	-		76	115	-	1(1)	1(1)	-	1(1)	1(1)	-	2%	2%	-	
	D	D	-		93	83	-	2(2)	2(2)	-	2(2)	2(2)	-	5%	4%	-	
putative sodium/potassium-transporting ATPase subunit beta-3	A	A	-	35639	79	81	-	1(1)	1(1)	-	1(1)	1(1)	-	3%	3%	-	KRY55118.1
	B	B	B		80	79	69	1(1)	1(1)	1(1)	1(1)	1(1)	1(1)	3%	3%	3%	
	-	C	-		-	77	-	-	1(1)	-	-	1(1)	-	-	3%	-	
putative splicing factor, arginine/serine-rich 7, partial	-	A	A	137028	-	64	64	-	1(0)	1(1)	-	1(0)	1(1)	-	1%	0%	KRY51241.1
	-	-	B		-	-	65	-	-	1(1)	-	-	1(1)	-	-	1%	
	C	-	C		70	-	63	1(1)	-	1(1)	1(1)	-	1(1)	1%	-	1%	
	-	D	-		-	64	-	-	1(1)	-	-	1(1)	-	-	1%	-	
	-	-	E		-	-	69	-	-	1(1)	-	-	1(1)	-	-	1%	
	-	-	G		-	-	71	-	-	1(1)	-	-	1(1)	-	-	1%	
putative tRNA (uracil-O(2)-)-methyltransferase, partial	-	C	-	72192	-	63	-	-	1(1)	-	-	1(1)	-	-	1%	-	KRY57299.1
putative UDP-glucuronosyltransferase ugt-58	-	C	-	63880	-	77	-	-	2(2)	-	-	2(2)	-	-	4%	-	KRY54469.1
Pyruvate carboxylase 1	-	-	E	129873	-	-	145	-	-	3(3)	-	-	3(3)	-	-	3%	KRY49943.1
Pyruvate dehydrogenase E1 component subunit beta, mitochondrial	C	-	-	75458	103	-	-	1(1)	-	-	1(1)	-	-	2%	-	-	KRY57570.1
	-	D	-		-	65	-	-	1(1)	-	-	1(1)	-	-	1%	-	
Pyruvate kinase PKLR	-	-	C	69929	-	-	170	-	-	2(2)	-	-	2(2)	-	-	5%	KRY57523.1
Pyruvate kinase PKM	-	-	C	65506	-	-	170	-	-	2(2)	-	-	2(2)	-	-	5%	KRY57524.1
Rab GDP dissociation inhibitor alpha	A	A	A	50324	262	253	325	6(6)	6(6)	8(8)	6(6)	6(6)	7(7)	18%	12%	20%	KRY54650.1

	B	B	B		727	930	766	19(19)	24(23)	22(22)	13(13)	15(14)	16(16)	39%	45%	47%	
Rab proteins geranylgeranyltransferase component A	C	C	-	63173	173	92	-	2(2)	1(1)	-	2(2)	1(1)	-	6%	3%	-	KRY47219.1
Ran GTPase-activating protein 1, partial	C	C	C	66199	275	258	205	7(7)	7(7)	6(6)	6(6)	7(7)	6(6)	11%	12%	11%	KRY60944.1
RANBP2-like and GRIP domain-containing protein 5/6, partial	-	-	G	112852	-	-	88	-	-	1(1)	-	-	1(1)	-	-	1%	KRY49040.1
Regulator of chromosome condensation	-	-	B	74425	-	-	69	-	-	1(1)	-	-	1(1)	-	-	1%	KRY55803.1
Replicase polyprotein 1a	A	-	A	82573	77	-	94	1(1)	-	1(1)	1(1)	-	1(1)	1%	-	1%	KRY55933.1
	-	-	B		-	-	77	-	-	1(1)	-	-	1(1)	-	-	1%	
Rho GTPase-activating protein 39	-	B	-	229114	-	126	-	-	2(2)	-	-	2(2)	-	-	1%	-	KRY55506.1
RNA-binding protein squid	-	A	A	38365	-	89	105	-	1(1)	1(1)	-	1(1)	1(1)	-	4%	4%	KRY50692.1
RWD domain-containing protein 2B	A	-	-	79837	88	-	-	1(1)	-	-	1(1)	-	-	1%	-	-	KRY47777.1
	B	B	B		71	116	133	1(1)	2(2)	2(2)	1(1)	2(2)	2(2)	1%	3%	3%	
	D	D	D		156	74	94	4(4)	1(1)	2(2)	4(4)	1(1)	2(2)	3%	2%	3%	
	-	-	E		-	-	147	-	-	3(3)	-	-	3(3)	-	-	6%	
	-	-	F		-	-	65	-	-	1(1)	-	-	1(1)	-	-	2%	
	-	-	G		-	-	82	-	-	1(1)	-	-	1(1)	-	-	1%	
S1 RNA-binding domain-containing protein 1, partial	-	D	-	121011	-	62	-	-	1(1)	-	-	1(1)	-	-	0%	-	KRY57178.1
secretion antigen precursor	A	-	-	46673	88	-	-	1(1)	-	-	1(1)	-	-	2%	-	-	CAD86782.1
	B	B	B		71	116	133	1(1)	2(2)	2(2)	1(1)	2(2)	2(2)	2%	5%	5%	
	C	C	C		1200	939	735	33(33)	25(24)	20(20)	16(16)	11(10)	13(13)	39%	35%	32%	
	D	D	D		156	74	94	4(4)	1(1)	2(2)	3(3)	1(1)	2(2)	6%	3%	5%	
	-	-	E		-	-	147	-	-	3(3)	-	-	3(3)	-	-	10%	
	-	-	F		-	-	65	-	-	1(1)	-	-	1(1)	-	-	3%	
	-	-	G		-	-	82	-	-	1(1)	-	-	1(1)	-	-	2%	
Serine hydroxymethyltransferase	B	B	-	33657	122	130	-	1(1)	1(1)	-	1(1)	1(1)	-	4%	4%	-	KRY56265.1
	-	-	E		-	-	112	-	-	1(1)	-	-	1(1)	-	-	4%	
	-	-	G		-	-	91	-	-	1(1)	-	-	1(1)	-	-	4%	
Serine protease 30	A	A	A	146279	695	228	522	11(10)	4(4)	8(7)	6(5)	4(4)	5(4)	7%	5%	8%	KRY58841.1
	B	B	B		697	919	930	13(13)	15(15)	15(15)	6(6)	6(6)	7(7)	6%	7%	8%	
	C	C	C		1561	876	1129	29(29)	14(14)	19(19)	7(7)	7(7)	7(7)	8%	10%	7%	
	D	D	D		531	253	150	7(7)	5(5)	3(3)	5(5)	5(5)	2(2)	6%	5%	2%	
	-	-	E		-	-	246	-	-	5(5)	-	-	4(4)	-	-	4%	
	-	-	F		-	-	71	-	-	1(1)	-	-	1(1)	-	-	1%	
	-	-	G		-	-	77	-	-	1(1)	-	-	1(1)	-	-	0%	
Serine/threonine-protein kinase mig-15	D	D	-	236043	257	133	-	5(5)	2(2)	-	5(5)	2(2)	-	4%	1%	-	KRY51313.1
Serine/threonine-protein phosphatase 2A 56 kDa regulatory subunit delta isoform, partial	-	C	-	73972	-	82	-	-	1(1)	-	-	1(1)	-	-	2%	-	KRY60738.1
Serine/threonine-protein phosphatase 2A 65 kDa regulatory subunit A alpha isoform	-	-	B	72963	-	-	107	-	-	1(1)	-	-	1(1)	-	-	2%	KRY51863.1
	-	D	-		-	130	-	-	2(2)	-	-	2(2)	-	-	5%	-	
	-	-	E		-	-	115	-	-	1(1)	-	-	1(1)	-	-	2%	
	-	-	F		-	-	93	-	-	1(1)	-	-	1(1)	-	-	2%	
Serine/threonine-protein phosphatase 2B catalytic subunit 2	-	-	B	55902	-	-	76	-	-	1(1)	-	-	1(1)	-	-	2%	KRY57383.1
Serine/threonine-protein phosphatase PP1	-	-	B	36096	-	-	76	-	-	1(1)	-	-	1(1)	-	-	3%	KRY53483.1
Serine--tRNA ligase, cytoplasmic	B	B	B	204653	78	145	151	1(1)	3(3)	2(2)	1(1)	3(3)	2(2)	0%	2%	2%	KRY54661.1
	-	C	-		-	100	-	-	1(1)	-	-	1(1)	-	-	0%	-	
Serrate RNA effector molecule -like protein	D	-	-	104611	112	-	-	3(3)	-	-	3(3)	-	-	3%	-	-	KRY49278.1
Signal recognition particle receptor subunit alpha -like protein	-	B	B	73535	-	83	101	-	1(1)	1(1)	-	1(1)	1(1)	-	1%	1%	KRY60530.1
Signal recognition particle subunit SRP72	C	-	C	76023	65	-	78	1(1)	-	1(1)	1(1)	-	1(1)	1%	-	1%	KRY48637.1

Snake venom 5'-nucleotidase	B	-	-	183199	112	-	-	2(2)	-	-	2(2)	-	-	2%	-	-	KRY49427.1
	C	C	C		1377	911	924	36(36)	26(24)	23(23)	18(18)	15(15)	16(16)	13%	12%	10%	
	D	D	-		60	155	-	1(1)	2(2)	-	1(1)	2(2)	-	1%	1%	-	
	-	-	E		-	-	124	-	-	2(2)	-	-	2(2)	-	-	1%	
	-	-	F		-	-	63	-	-	1(0)	-	-	1(0)	-	-	0%	
	-	-	G		-	-	98	-	-	1(1)	-	-	1(1)	-	-	0%	
Sodium/calcium exchanger 1, partial	D	D	-	93898	143	67	-	2(2)	1(1)	-	2(2)	1(1)	-	3%	1%	-	KRY49530.1
Sodium/potassium-transporting ATPase subunit alpha	C	-	-	115783	93	-	-	1(1)	-	-	1(1)	-	-	1%	-	-	KRY48181.1
	D	D	D		249	309	113	7(7)	8(8)	2(2)	7(7)	8(8)	2(2)	8%	9%	2%	
	-	-	E		-	-	202	-	-	6(6)	-	-	6(6)	-	-	8%	
	-	-	F		-	-	180	-	-	5(4)	-	-	5(4)	-	-	6%	
	-	-	G		-	-	184	-	-	5(5)	-	-	5(5)	-	-	6%	
Solute carrier family 2, facilitated glucose transporter member 3	A	A	A	167928	100	72	141	1(1)	1(1)	2(2)	1(1)	1(1)	2(2)	0%	0%	1%	KRY47169.1
	B	B	B		182	165	159	3(3)	3(3)	2(2)	3(3)	3(3)	2(2)	2%	2%	1%	
	-	C	-		-	60	-	-	1(1)	-	-	1(1)	-	-	0%	-	
	D	D	-		81	78	-	1(1)	1(1)	-	1(1)	1(1)	-	0%	0%	-	
	-	-	E		-	-	127	-	-	2(2)	-	-	2(2)	-	-	1%	
	-	-	F		-	-	73	-	-	1(1)	-	-	1(1)	-	-	0%	
	-	-	G		-	-	135	-	-	2(2)	-	-	2(2)	-	-	1%	
Solute carrier family 35 member F6	-	-	E	42929	-	-	78	-	-	1(1)	-	-	1(1)	-	-	2%	KRY61494.1
	-	-	G		-	-	79	-	-	1(1)	-	-	1(1)	-	-	2%	
Sorting nexin lst-4	-	-	C	78100	-	-	69	-	-	1(1)	-	-	1(1)	-	-	1%	KRY54778.1
Spectrin alpha chain	C	C	C	326074	131	62	86	2(2)	1(1)	1(1)	2(2)	1(1)	1(1)	1%	0%	0%	KRY60692.1
	D	D	D		449	342	105	14(14)	15(14)	6(6)	13(13)	14(13)	6(6)	6%	5%	2%	
	-	-	E		-	-	725	-	-	35(35)	-	-	33(33)	-	-	12%	
	-	-	F		-	-	589	-	-	28(27)	-	-	26(26)	-	-	10%	
	-	-	G		-	-	1111	-	-	46(45)	-	-	42(41)	-	-	17%	
Spectrin beta chain	C	C	-	270460	139	87	-	2(2)	1(1)	-	2(2)	1(1)	-	1%	0%	-	KRY58047.1
	D	D	D		604	548	181	17(17)	19(17)	6(6)	16(16)	19(17)	6(6)	9%	8%	3%	
	-	-	E		-	-	525	-	-	18(18)	-	-	18(18)	-	-	9%	
	-	-	F		-	-	499	-	-	22(21)	-	-	19(19)	-	-	9%	
	-	-	G		-	-	653	-	-	25(24)	-	-	24(23)	-	-	11%	
Spliceosome RNA helicase DDX39B	-	-	A	83161	-	-	68	-	-	1(1)	-	-	1(1)	-	-	1%	KRY50703.1
	B	-	B		80	-	218	2(2)	-	5(5)	2(2)	-	5(5)	2%	-	8%	
	-	C	C		-	80	85	-	1(1)	1(1)	-	1(1)	1(1)	-	1%	1%	
	-	D	-		-	79	-	-	1(1)	-	-	1(1)	-	-	1%	-	
Splicing factor 3A subunit 1, partial	-	B	-	116221	-	84	-	-	1(1)	-	-	1(1)	-	-	1%	-	KRY50075.1
	D	-	-		66	-	-	1(1)	-	-	1(1)	-	-	0%	-	-	
Splicing factor 3A subunit 3	C	C	C	58548	208	115	108	3(3)	3(3)	1(1)	3(3)	3(3)	1(1)	9%	7%	2%	KRY56136.1
Splicing factor 3B subunit 3	-	-	E	137455	-	-	152	-	-	3(3)	-	-	3(3)	-	-	2%	KRY50462.1
Squamous cell carcinoma antigen recognized by T-cells 3	A	-	-	143419	109	-	-	1(1)	-	-	1(1)	-	-	1%	-	-	KRY55648.1
	D	-	-		116	-	-	1(1)	-	-	1(1)	-	-	1%	-	-	
Stress-70 protein, mitochondrial	A	A	A	92371	60	63	72	1(1)	1(0)	1(1)	1(1)	1(0)	1(1)	1%	1%	1%	KRY58424.1
	C	C	C		154	164	127	6(5)	4(4)	4(4)	6(5)	4(4)	4(4)	7%	5%	5%	
	D	D	-		112	104	-	2(2)	2(2)	-	2(2)	2(2)	-	3%	2%	-	
	-	-	E		-	-	101	-	-	2(2)	-	-	2(2)	-	-	3%	
	-	-	G		-	-	93	-	-	1(1)	-	-	1(1)	-	-	1%	
Stress-induced-phosphoprotein 1	A	A	A	59744	89	110	68	1(1)	2(2)	1(1)	1(1)	2(2)	1(1)	2%	5%	2%	KRY50816.1
	C	C	C		766	591	982	24(24)	23(21)	32(31)	22(22)	18(16)	20(19)	45%	39%	42%	

Structural maintenance of chromosomes protein 3	B	B	B	180414	125	99	148	3(3)	2(2)	5(5)	3(3)	2(2)	4(4)	1%	1%	3%	KRY53012.1
Succinate dehydrogenase [ubiquinone] flavoprotein subunit, mitochondrial	C	C	C	72418	677	534	669	21(21)	18(17)	16(15)	17(17)	14(13)	12(11)	33%	25%	23%	KRY54543.1
Succinate dehydrogenase [ubiquinone] iron-sulfur subunit, mitochondrial	D	-	-	80501	87	-	-	1(1)	-	-	1(1)	-	-	1%	-	-	KRY59439.1
	-	-	E		-	-	72	-	-	1(1)	-	-	1(1)	-	-	2%	
Succinate-semialdehyde dehydrogenase, mitochondrial	B	B	B	63166	152	163	251	5(5)	6(6)	7(7)	5(5)	6(6)	7(7)	10%	11%	14%	KRY54971.1
	D	D	-		79	76	-	1(1)	2(2)	-	1(1)	2(2)	-	2%	4%	-	
Sucrase-isomaltase, intestinal, partial	-	-	E	110992	-	-	165	-	-	5(4)	-	-	5(4)	-	-	6%	KRY53086.1
Sulfhydryl oxidase 1	C	C	C	109353	446	372	231	11(11)	9(9)	7(7)	8(8)	7(7)	6(6)	10%	10%	9%	KRY58937.1
Sulfide:quinone oxidoreductase, mitochondrial	A	A	A	54403	185	64	112	5(5)	1(1)	3(3)	5(5)	1(1)	3(3)	14%	2%	6%	KRY46292.1
	-	B	B		-	80	65	-	1(1)	1(1)	-	1(1)	1(1)	-	2%	2%	
	-	C	-		-	83	-	-	1(1)	-	-	1(1)	-	-	2%	-	
	D	D	-		64	81	-	1(1)	2(2)	-	1(1)	2(2)	-	2%	5%	-	
Talin-1	D	-	-	282559	71	-	-	1(1)	-	-	1(1)	-	-	0%	-	-	KRY54739.1
TBC1 domain family member 15, partial	-	C	-	78010	-	111	-	-	1(1)	-	-	1(1)	-	-	1%	-	KRY52268.1
T-complex protein 1 subunit alpha	-	A	-	79972	-	71	-	-	1(1)	-	-	1(1)	-	-	1%	-	KRY49588.1
	C	C	C		266	95	136	6(6)	2(2)	4(4)	6(6)	2(2)	4(4)	9%	2%	6%	
	-	D	-		-	73	-	-	1(1)	-	-	1(1)	-	-	1%	-	
	-	-	E		-	-	142	-	-	3(3)	-	-	3(3)	-	-	5%	
	-	-	G		-	-	74	-	-	1(1)	-	-	1(1)	-	-	1%	
T-complex protein 1 subunit beta	-	-	A	59807	-	-	62	-	-	1(1)	-	-	1(1)	-	-	2%	KRY52365.1
	B	B	B		138	116	113	2(2)	3(3)	2(2)	2(2)	3(3)	2(2)	5%	8%	4%	
	C	C	C		260	236	265	7(7)	5(5)	8(8)	7(7)	5(5)	8(8)	16%	13%	20%	
	D	D	D		76	84	94	1(1)	1(1)	1(1)	1(1)	1(1)	1(1)	2%	2%	2%	
	-	-	E		-	-	107	-	-	1(1)	-	-	1(1)	-	-	2%	
	-	-	F		-	-	102	-	-	1(1)	-	-	1(1)	-	-	2%	
T-complex protein 1 subunit delta, partial	-	-	C	25524	-	-	64	-	-	1(1)	-	-	1(1)	-	-	3%	KRY50590.1
	-	-	E		-	-	70	-	-	1(1)	-	-	1(1)	-	-	5%	
	-	-	G		-	-	69	-	-	1(1)	-	-	1(1)	-	-	5%	
T-complex protein 1 subunit epsilon	-	-	B	66843	-	-	74	-	-	1(1)	-	-	1(1)	-	-	1%	KRY50385.1
	C	C	C		595	503	322	17(17)	16(16)	11(11)	16(16)	14(14)	7(7)	29%	27%	13%	
	-	-	E		-	-	146	-	-	2(2)	-	-	2(2)	-	-	5%	
	-	-	G		-	-	77	-	-	2(2)	-	-	2(2)	-	-	5%	
T-complex protein 1 subunit eta	-	A	A	175156	-	99	109	-	1(1)	2(2)	-	1(1)	2(2)	-	0%	1%	KRY48081.1
	-	B	B		-	96	70	-	2(2)	1(1)	-	2(2)	1(1)	-	2%	0%	
	C	C	-		151	126	-	3(3)	2(2)	-	3(3)	2(2)	-	2%	1%	-	
	D	D	-		131	83	-	1(1)	1(1)	-	1(1)	1(1)	-	0%	0%	-	
	-	-	F		-	-	82	-	-	1(1)	-	-	1(1)	-	-	0%	
	-	-	G		-	-	83	-	-	1(1)	-	-	1(1)	-	-	0%	
T-complex protein 1 subunit gamma	-	B	B	60486	-	99	142	-	1(1)	2(2)	-	1(1)	2(2)	-	2%	5%	KRY47292.1
	C	C	C		647	427	436	16(16)	14(12)	9(9)	12(12)	10(9)	7(7)	32%	24%	19%	
	D	-	-		140	-	-	2(2)	-	-	2(2)	-	-	5%	-	-	
	-	-	E		-	-	64	-	-	1(1)	-	-	1(1)	-	-	1%	
T-complex protein 1 subunit theta	-	-	A	151283	-	-	119	-	-	1(1)	-	-	1(1)	-	-	1%	KRY50910.1
	B	B	B		149	128	75	4(4)	1(1)	2(2)	4(4)	1(1)	2(2)	3%	1%	1%	
	C	C	C		242	201	154	7(7)	7(7)	4(4)	7(7)	7(7)	4(4)	6%	6%	3%	
	D	D	D		242	214	86	6(6)	5(5)	1(1)	6(6)	5(5)	1(1)	5%	4%	1%	
	-	-	E		-	-	131	-	-	4(4)	-	-	4(4)	-	-	3%	
	-	-	F		-	-	173	-	-	4(4)	-	-	4(4)	-	-	3%	

	-	-	G		-	-	185	-	-	3(3)	-	-	3(3)	-	-	2%	
Tenascin-X	A	A	-	43639	78	74	-	2(2)	1(1)	-	2(2)	1(1)	-	6%	3%	-	KRY57379.1
Teneurin-a	D	-	-	161014	204	-	-	4(4)	-	-	4(4)	-	-	3%	-	-	KRY46006.1
Tetratricopeptide repeat protein 39B, partial	-	C	-	67183	-	76	-	-	1(1)	-	-	1(1)	-	-	1%	-	KRY50883.1
Thioredoxin reductase 1, cytoplasmic, partial	D	D	-	141583	67	68	-	1(1)	1(1)	-	1(1)	1(1)	-	0%	0%	-	KRY57019.1
	-	-	F		-	-	71	-	-	1(1)	-	-	1(1)	-	-	0%	
	-	-	G		-	-	87	-	-	1(1)	-	-	1(1)	-	-	0%	
TIP41-like protein, partial	D	-	-	251313	105	-	-	3(3)	-	-	3(3)	-	-	2%	-	-	KRY48002.1
Titin	-	-	B	1732268	-	-	71	-	-	1(1)	-	-	1(1)	-	-	0%	KRY55434.1
	-	-	C		-	-	69	-	-	1(1)	-	-	1(1)	-	-	0%	
	-	D	-		-	67	-	-	1(1)	-	-	1(1)	-	-	0%	-	
	-	-	E		-	-	123	-	-	5(5)	-	-	5(5)	-	-	0%	
	-	-	F		-	-	88	-	-	3(3)	-	-	3(3)	-	-	0%	
	-	-	G		-	-	92	-	-	2(2)	-	-	2(2)	-	-	0%	
TOM1-like protein 2	-	-	A	44804	-	-	103	-	-	1(1)	-	-	1(1)	-	-	3%	KRY50457.1
	-	B	B		-	112	89	-	1(1)	1(1)	-	1(1)	1(1)	-	3%	2%	
Transforming growth factor-beta-induced protein ig-h3, partial	-	C	-	82807	-	98	-	-	2(2)	-	-	2(2)	-	-	3%	-	KRY46587.1
	D	D	D		586	369	169	23(22)	19(16)	6(6)	18(17)	16(15)	6(6)	27%	23%	8%	
Transketolase	C	C	C	140272	685	486	530	19(19)	16(15)	19(18)	16(16)	13(12)	17(16)	13%	11%	14%	KRY48205.1
	D	D	-		97	79	-	2(2)	1(1)	-	2(2)	1(1)	-	2%	0%	-	
	-	-	E		-	-	120	-	-	2(2)	-	-	2(2)	-	-	2%	
	-	-	F		-	-	81	-	-	1(1)	-	-	1(1)	-	-	0%	
	-	-	G		-	-	79	-	-	1(1)	-	-	1(1)	-	-	0%	
Transketolase-like protein 2	-	-	E	140714	-	-	120	-	-	2(2)	-	-	2(2)	-	-	2%	KRY48207.1
	-	-	F		-	-	81	-	-	1(1)	-	-	1(1)	-	-	0%	
	-	-	G		-	-	79	-	-	1(1)	-	-	1(1)	-	-	0%	
Transmembrane 9 superfamily member 4, partial	B	-	-	75001	66	-	-	1(1)	-	-	1(1)	-	-	1%	-	-	KRY58234.1
Transmembrane protease serine 5	A	A	A	41206	148	148	116	2(2)	4(4)	3(3)	2(2)	4(4)	3(3)	3%	11%	9%	KRY50806.1
Transmembrane protease serine 9	-	A	A	73208	-	123	191	-	1(1)	5(5)	-	1(1)	5(5)	-	2%	9%	KRY59262.1
	B	B	B		65	185	156	1(1)	4(4)	3(3)	1(1)	3(3)	3(3)	1%	5%	5%	
	C	C	C		654	495	593	17(16)	13(13)	16(16)	15(14)	9(9)	11(11)	26%	14%	20%	
	D	D	D		156	149	126	6(5)	2(2)	2(2)	6(5)	2(2)	2(2)	11%	4%	4%	
	-	-	E		-	-	203	-	-	4(4)	-	-	4(4)	-	-	5%	
	-	-	F		-	-	150	-	-	3(3)	-	-	3(3)	-	-	5%	
	-	-	G		-	-	100	-	-	2(2)	-	-	2(2)	-	-	2%	
Trifunctional enzyme subunit alpha, mitochondrial, partial	-	D	-	87742	-	61	-	-	1(1)	-	-	1(1)	-	-	1%	-	KRY46110.1
	-	-	E		-	-	71	-	-	1(1)	-	-	1(1)	-	-	1%	
	-	-	F		-	-	69	-	-	1(1)	-	-	1(1)	-	-	1%	
	-	-	G	85474	-	-	72	-	-	2(2)	-	-	2(2)	-	-	2%	KRY50419.1
Trifunctional enzyme subunit beta, mitochondrial	A	A	A	47839	102	118	114	2(2)	3(3)	4(4)	2(2)	3(3)	4(4)	5%	6%	8%	KRY48050.1
	-	-	E		-	-	66	-	-	1(1)	-	-	1(1)	-	-	2%	
Tripeptidyl-peptidase 2	-	-	D	148415	-	-	76	-	-	1(1)	-	-	1(1)	-	-	0%	KRY49250.1
	-	-	E		-	-	85	-	-	2(2)	-	-	2(2)	-	-	1%	
	-	-	F		-	-	78	-	-	1(1)	-	-	1(1)	-	-	0%	
Tubulin alpha-3 chain	A	A	A	50924	169	130	114	3(3)	3(3)	3(3)	3(3)	3(3)	3(3)	9%	11%	10%	KRY50534.1
	B	B	B		286	280	429	6(6)	6(6)	10(10)	6(6)	5(5)	9(9)	20%	16%	28%	
	C	C	C		221	253	281	5(5)	7(7)	4(4)	5(5)	7(7)	4(4)	16%	20%	14%	
	D	D	D		324	208	73	6(6)	6(6)	1(1)	6(6)	6(6)	1(1)	23%	18%	4%	
	-	-	E		-	-	156	-	-	4(4)	-	-	4(4)	-	-	13%	

	-	-	F		-	-	82	-	-	2(2)	-	-	2(2)	-	-	6%	
	-	-	G		-	-	92	-	-	3(3)	-	-	3(3)	-	-	8%	
Twitchin	-	D	-	712509	-	60	-	-	1(1)	-	-	1(1)	-	-	0%	-	KRY50893.1
	-	-	E		-	-	218	-	-	8(8)	-	-	8(8)	-	-	1%	
	-	-	F		-	-	116	-	-	3(3)	-	-	3(3)	-	-	0%	
	-	-	G		-	-	379	-	-	15(15)	-	-	15(15)	-	-	2%	
Type I inositol 1,4,5-trisphosphate 5-phosphatase, partial	A	A	A	57850	210	86	99	6(6)	1(1)	2(2)	5(5)	1(1)	2(2)	14%	2%	4%	KRY50866.1
U3 small nucleolar RNA-associated protein 15 -like protein	-	C	C	103477	-	60	61	-	1(1)	1(1)	-	1(1)	1(1)	-	0%	0%	KRY56987.1
Ubiquitin carboxyl-terminal hydrolase 14, partial	C	C	C	57580	201	109	174	7(7)	3(2)	6(6)	4(4)	3(2)	6(6)	10%	7%	14%	KRY58645.1
	-	-	E		-	-	81	-	-	1(1)	-	-	1(1)	-	-	2%	
Ubiquitin carboxyl-terminal hydrolase 15	-	-	C	155087	-	-	107	-	-	1(1)	-	-	1(1)	-	-	1%	KRY54182.1
	-	-	D		-	-	161	-	-	4(4)	-	-	4(4)	-	-	4%	
Ubiquitin carboxyl-terminal hydrolase 4	-	-	C	163219	-	-	107	-	-	1(1)	-	-	1(1)	-	-	1%	KRY54180.1
	-	-	D		-	-	161	-	-	4(4)	-	-	4(4)	-	-	4%	
Ubiquitin carboxyl-terminal hydrolase 7, partial	C	-	-	127949	61	-	-	1(1)	-	-	1(1)	-	-	1%	-	-	KRY49807.1
	-	D	-		-	104	-	-	2(2)	-	-	2(2)	-	-	2%	-	
	-	-	E		-	-	258	-	-	9(8)	-	-	9(8)	-	-	9%	
Ubiquitin-like modifier-activating enzyme 1	D	D	D	128939	221	221	105	4(4)	5(5)	2(2)	4(4)	5(5)	2(2)	7%	6%	2%	KRY59617.1
	-	-	F		-	-	153	-	-	2(2)	-	-	2(2)	-	-	2%	
	-	-	G		-	-	166	-	-	4(4)	-	-	4(4)	-	-	4%	
Ubiquitin-like modifier-activating enzyme 5	A	A	A	41307	104	103	174	1(1)	1(1)	2(2)	1(1)	1(1)	2(2)	4%	3%	7%	KRY47667.1
UBX domain-containing protein 7, partial	-	-	B	53187	-	-	77	-	-	1(1)	-	-	1(1)	-	-	2%	KRY58943.1
UDP-glucose 4-epimerase	A	-	-	86965	100	-	-	1(1)	-	-	1(1)	-	-	1%	-	-	KRY59504.1
UDP-glucose:glycoprotein glucosyltransferase 1	D	-	-	407891	76	-	-	1(1)	-	-	1(1)	-	-	0%	-	-	KRY46767.1
	-	-	E		-	-	107	-	-	5(4)	-	-	5(4)	-	-	0%	
	-	-	F		-	-	147	-	-	3(3)	-	-	3(3)	-	-	1%	
UDP-N-acetylhexosamine pyrophosphorylase	-	-	A	84084	-	-	62	-	-	1(1)	-	-	1(1)	-	-	1%	KRY50795.1
	-	-	B		-	-	63	-	-	1(1)	-	-	1(1)	-	-	1%	
	-	-	C		-	-	213	-	-	4(4)	-	-	4(4)	-	-	7%	
Ufm1-specific protease 2, partial	C	C	C	72485	112	130	135	3(3)	4(4)	3(3)	3(3)	4(4)	3(3)	7%	8%	6%	KRY61155.1
Uncharacterized protein T03_12349, partial	B	B	B	51663	85	75	88	1(1)	1(1)	1(1)	1(1)	1(1)	1(1)	2%	2%	2%	KRY48646.1
Uncharacterized protein T03_14872	D	-	-	149786	61	-	-	1(1)	-	-	1(1)	-	-	0%	-	-	KRY46969.1
	-	-	E		-	-	165	-	-	5(5)	-	-	5(5)	-	-	4%	
	-	-	F		-	-	227	-	-	10(10)	-	-	10(10)	-	-	8%	
Uncharacterized protein T03_8348, partial	C	C	C	89811	58	76	96	1(1)	1(1)	1(1)	1(1)	1(1)	1(1)	1%	1%	1%	KRY58357.1
Uncharacterized protein T03_9851	A	A	A	42427	890	316	435	18(17)	6(6)	10(9)	6(5)	4(4)	6(5)	25%	14%	25%	KRY58607.1
	B	B	B		233	316	402	5(5)	5(5)	8(8)	3(3)	3(3)	5(5)	14%	14%	23%	
	-	-	D		-	-	70	-	-	1(1)	-	-	1(1)	-	-	3%	
	-	-	E		-	-	67	-	-	1(1)	-	-	1(1)	-	-	3%	
Uridine 5'-monophosphate synthase	B	-	B	108908	75	-	67	1(1)	-	1(1)	1(1)	-	1(1)	1%	-	1%	KRY46591.1
UTP--glucose-1-phosphate uridylyltransferase, partial	-	A	-	154987	-	88	-	-	1(1)	-	-	1(1)	-	-	0%	-	KRY53918.1
	B	-	B		69	-	75	1(1)	-	1(1)	1(1)	-	1(1)	0%	-	1%	
	-	-	E		-	-	83	-	-	1(1)	-	-	1(1)	-	-	0%	
	-	-	G		-	-	64	-	-	1(1)	-	-	1(1)	-	-	0%	
UV excision repair protein RAD23 -like protein B, partial	A	A	A	59386	64	67	66	1(1)	1(1)	1(1)	1(1)	1(1)	1(1)	3%	1%	1%	KRY52282.1
Vacuolar protein sorting-associated protein 13A	D	-	-	374278	65	-	-	1(1)	-	-	1(1)	-	-	0%	-	-	KRY49720.1
	-	-	E		-	-	129	-	-	2(2)	-	-	2(2)	-	-	0%	
	-	-	G		-	-	123	-	-	3(3)	-	-	3(3)	-	-	1%	
Vacuolar protein sorting-associated protein 13C	D	-	-	349964	65	-	-	1(1)	-	-	1(1)	-	-	0%	-	-	KRY49724.1

	-	-	E		-	-	129	-	-	2(2)	-	-	2(2)	-	-	0%	
Vacuolar protein sorting-associated protein 26B	-	B	B	215378	-	64	61	-	1(1)	1(1)	-	1(1)	1(1)	-	1%	0%	KRY53691.1
Vacuolar protein sorting-associated protein 45	C	C	C	152135	131	84	97	3(3)	1(1)	2(2)	3(3)	1(1)	2(2)	2%	1%	2%	KRY55299.1
Valine--tRNA ligase	-	D	-	120864	-	80	-	-	1(1)	-	-	1(1)	-	-	0%	-	KRY54004.1
Very long-chain specific acyl-CoA dehydrogenase, mitochondrial, partial	C	C	C	70153	368	152	159	10(10)	4(4)	3(3)	10(10)	4(4)	3(3)	21%	8%	6%	KRY52870.1
Vigilin, partial	D	-	-	156394	72	-	-	1(1)	-	-	1(1)	-	-	1%	-	-	KRY57752.1
	-	-	G		-	-	83	-	-	2(2)	-	-	2(2)	-	-	1%	
Vinculin	-	D	D	150968	-	269	150	-	7(7)	2(2)	-	7(7)	2(2)	-	6%	2%	KRY51468.1
	-	-	E		-	-	212	-	-	3(3)	-	-	3(3)	-	-	3%	
	-	-	G		-	-	77	-	-	1(1)	-	-	1(1)	-	-	0%	
Vitellogenin, partial	C	-	C	389675	81	-	74	1(1)	-	2(1)	1(1)	-	2(1)	0%	-	0%	KRY54946.1
	D	D	D		161	217	83	4(4)	5(5)	1(1)	4(4)	5(5)	1(1)	1%	1%	0%	
	-	-	E		-	-	217	-	-	10(10)	-	-	10(10)	-	-	3%	
	-	-	F		-	-	293	-	-	12(11)	-	-	11(10)	-	-	3%	
	-	-	G		-	-	365	-	-	15(14)	-	-	15(14)	-	-	4%	
Voltage-dependent calcium channel unc-36	-	-	F	87460	-	-	81	-	-	1(1)	-	-	1(1)	-	-	1%	KRY55466.1
WD repeat-containing protein 74	-	-	B	41222	-	-	65	-	-	1(1)	-	-	1(1)	-	-	1%	KRY47261.1
Xaa-Pro aminopeptidase 1, partial	C	C	-	74254	280	278	-	7(7)	9(9)	-	7(7)	8(8)	-	15%	16%	-	KRY57414.1
Y-box factor -like protein	B	B	B	34488	94	136	106	1(1)	2(2)	1(1)	1(1)	2(2)	1(1)	6%	11%	6%	KRY49695.1
	C	C	-		58	68	-	1(1)	1(1)	-	1(1)	1(1)	-	6%	6%	-	
Zygotic DNA replication licensing factor mcm6-B, partial	D	-	-	95134	80	-	-	1(1)	-	-	1(1)	-	-	1%	-	-	KRY61053.1
